# Extending Fibre Nonlinear Interference Power Modelling to Account for General Dual-Polarisation 4D Modulation Formats

**DOI:** 10.3390/e22111324

**Published:** 2020-11-20

**Authors:** Gabriele Liga, Astrid Barreiro, Hami Rabbani, Alex Alvarado

**Affiliations:** 1 Information and Communication Theory Lab, Signal Processing Systems Group, Department of Electrical Engineering, Eindhoven University of Technology, 5600 MB Eindhoven, The Netherlands; a.barreiro.berrio@tue.nl (A.B.); hami.rabbani@email.kntu.ac.ir (H.R.); a.alvarado@tue.nl (A.A.); 2 Department of Electrical Engineering, K. N. Toosi University of Technology, Tehran 1355-16315, Iran

**Keywords:** 4D modulation formats, optical communications, channel modelling

## Abstract

In optical communications, four-dimensional (4D) modulation formats encode information onto the quadrature components of two arbitrary orthogonal states of polarisation of the optical field. Many analytical models available in the optical communication literature allow, within a first-order perturbation framework, the computation of the average power of the nonlinear interference (NLI) accumulated in coherent fibre-optic transmission systems. However, all such models only operate under the assumption of transmitted polarisation-multiplexed two-dimensional (PM-2D) modulation formats, which only represent a limited subset of the possible dual-polarisation 4D (DP-4D) formats. Namely, only those where data transmitted on each polarisation channel are mutually independent and identically distributed. This paper presents a step-by-step mathematical derivation of the extension of existing NLI models to the class of arbitrary DP-4D modulation formats. In particular, the methodology adopted follows the one of the popular enhanced Gaussian noise model, albeit dropping most assumptions on the geometry and statistic of the transmitted 4D modulation format. The resulting expressions show that, whilst in the PM-2D case the NLI power depends only on different statistical high-order moments of each polarisation component, for a general DP-4D constellation, several other cross-polarisation correlations also need to be taken into account.

## 1. Introduction

With the resurgence of polarisation-diverse, optical coherent detection, transmission of information over an optical fibre is typically performed exploiting four degrees of freedom of the optical field: two quadrature components over two orthogonal states of polarisation. The standard approach consists in encoding data independently over the two polarisation channels using the same two-dimensional (2D) modulation format. The resulting four-dimensional (4D) constellation is often referred to as a polarisation-multiplexed 2D (PM-2D) modulation format. The strong point of PM-2D formats is their simplicity of generation and performance analysis: as the two polarisation channels are independent and under the assumption of data-independent cross-polarisation interference in the fibre channel, transmission performance can be evaluated using the 2D component format.

Despite the popularity of PM-2D formats, a substantial amount of research work in the literature has been devoted to more general 4D formats, i.e., 4D constellations which are not necessarily generated as Cartesian products of a component 2D constellation [1,2]. These formats have recently regained attention due to their potential power efficiency, nonlinearity tolerance, and ultimately their still unexplored shaping gains. The reason for that relies on the fact that, by exploiting the full 4D space, sensitivity and other relevant performance metrics such as mutual information or generalised mutual information can be improved compared to traditional PM-2D formats [3,4,5,6,7].

Previous works on optimised 4D modulation formats have either operated under an additive white Gaussian noise channel hypothesis [1,2,3] or exploited some heuristic approaches to derive nonlinearly tolerant formats in the fibre-optic channel [5,6,7]. However, accurately predicting the amount of nonlinear interference generated by transmission of a given constellation in an optical fibre is key to optimising its shape in *N* dimensions.

Modelling of nonlinear interference (NLI) in optical fibre transmission is quite a mature field of research where an impressive amount of progress was made in the first half of the 2010s, e.g., in [8,9,10,11]. In particular, [10,11] introduced for the first time the possibility of predicting the dependency of nonlinear interference power as a function of the modulation format features, i.e., geometrical shape and statistical properties. Among other assumptions, one underlying key point of all previous models is the transmission of PM-2D modulation formats, where data on the two polarisation channels are assumed to be independent and identically distributed. Under this constraint, one can predict the NLI power using the statistical properties of the 2D component modulation format. It is clear, however, that this approach ceases to be applicable to general DP-4D formats, where a single 2D component format might not even exist.

In this work, we extend the existing analytical expressions for NLI power to account for DP-4D constellations where the two 2D polarisation components are not identically distributed or when there is statistical dependency between them. The undertaken approach is the same as in [9], i.e., a frequency-domain, first-order perturbation study. Unlike [9], no assumptions are made on either the marginal or joint statistics of the two polarisation components of the transmitted 4D constellation (besides being zero-mean). The final expressions reveal the impact of several different (nontrivial) cross-polarisation statistics on the NLI power.

The formulas presented in this work enable an accurate computation of the NLI power for all possible dual-polarisation formats in optical fibre transmission. As a result, a reliable optimisation of both geometry and symbol probability of occurrence of such 4D formats is also enabled for the optical fibre channel.

## 2. Organisation of the Manuscript and Notation

**Figure 1:**
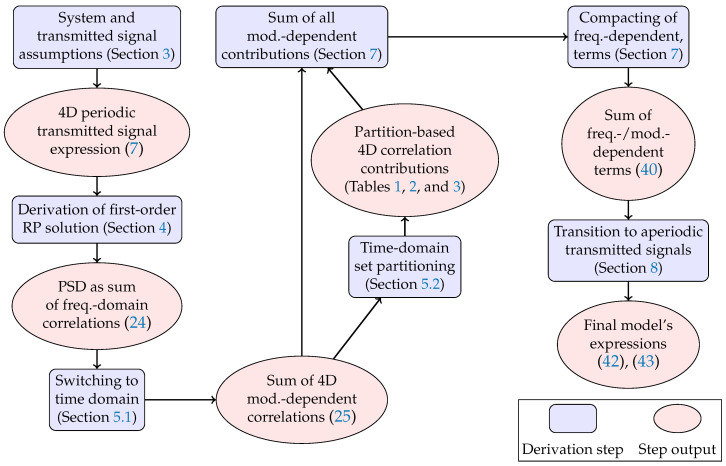
Flowchart of the analytical derivation in this work.

The manuscript is organised as follows: (i) in Section 3, the investigated system model is described and the model assumptions are presented; (ii) Section 4, Section 5, Section 6, Section 7 and Section 8 are devoted to a step-by-step analytical derivation of the model; and (iii) ultimately, the main model expression is presented in Section 8. In particular: in Section 4, the regular perturbation (RP) solution to the frequency domain Manakov equation is derived for a multi-span fibre system and its power spectral density (PSD) is evaluated in the case of a transmitted periodic signal; in Section 5, the contributions of the different high-order moments and cross-polarisation correlations of the transmitted 4D modulation format are highlighted; finally, Section 8 derives, via Theorem 2, an expression for the PSD as the signal period is extended into infinity. A flowchart of the main derivation steps performed in this work, with their corresponding references in the manuscript, is shown in Figure 1.

Throughout this manuscript, we denote 2D (column) vectors with boldface letters (e.g., a), whereas 2D column vector functions are indicated with boldface capital letters (e.g., $E\left(f,z\right),\tilde{E}\left(t,z\right)$, etc.). For indicating the optical field, the first variable of represents either the time or frequency variable whereas the second one represents the fibre propagation section. An exception is made for the multi-span system case, where second and third variables are assigned to the number of spans and span length, respectively. This highlights the joint dependence of the output optical field on these two variables, as shown later in the paper. F{·}, E{·}, and Re{·} indicate the Fourier transform, the statistical expectation, and the real part operators, respectively. The delta distribution is indicated by δ(·), whereas $\delta_{k}$ denotes the Kronecker delta defined as

$$\delta_{k}≜\left\{\begin{array}{c} 1\;\mathrm{for}\;k=0,\\ 0\;\mathrm{elsewhere}. \end{array}\right.$$

Finally, Z, R, and C denote the integer, real, and complex fields, respectively, and *j* is the imaginary unit.

## 3. Model Assumptions

### 3.1. System Model

**Figure 2:**
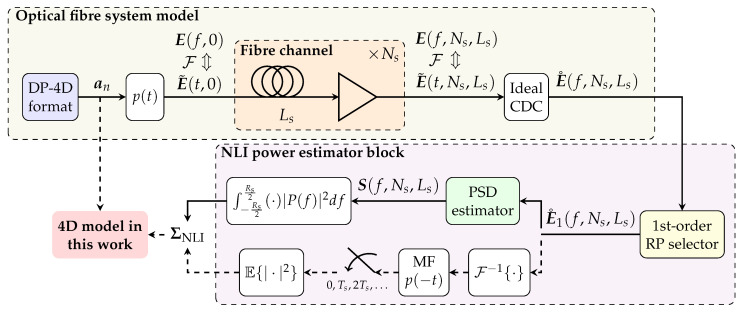
System model under investigation in this work which consists of an optical fibre system model and a nonlinear interference (NLI) variance estimation block: the two branches in the NLI variance estimator block indicate alternative ways of estimating the NLI variance $\Sigma_{\mathrm{NLI}}$.

The baseband equivalent model of the optical fibre system under investigation in this work is shown in Figure 2. The fibre channel is a multi-span fibre system using Erbium-doped fibre amplification (EDFA). In this manuscript, it is assumed that a single-channel signal is transmitted. The transmitter is assumed to generate for each symbol period *n* the 4D symbol $a_{n}={\left[a_{x,n},a_{y,n}\right]}^{T}$, where $a_{x,n},a_{y,n}\in C$ are complex symbols modulated on two arbitrary orthogonal polarisation states *x* and *y*, respectively. The sequence of symbols $a_{n}$ for n∈Z is assumed to be a cyclostationary process of period *W*. The set of random variables (RVs) within each period of such process are also assumed to be statistically independent. Linear modulation with a single, real pulse p(t) on *x* and *y* polarisation is adopted. The pulse p(t) with spectrum P(f) is assumed to be strictly band-limited within the range of frequencies $\left[-R_{s}/2,R_{s}/2\right]$. As discussed in Section 3.3, the transmitted signal $\tilde{E}\left(t,0\right)$ is assumed to be periodic with period *T*, such that

(1) $$\tilde{E}\left(t,0\right)=\sum_{n=-(W-1)/2}^{(W-1)/2}a_{n}p\left(t-nT_{s}\right),\;\mathrm{for}\;\;0\leq t\leq T,$$

**Figure 3:**
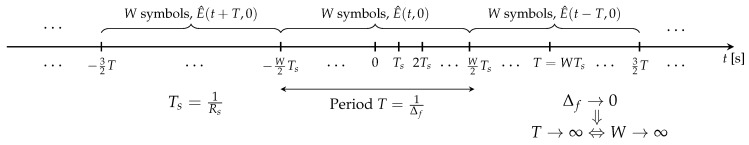
Schematic representation of the periodic signal assumption, where *W* symbols are transmitted every *T* [s], each symbol with a duration of $T_{s}$ [s]: the periodicity assumption will be lifted in Section 8 by letting $\Delta_{f}\to 0$.

$T_{s}=1/R_{s}=T/W$ represents the symbol period, and $R_{s}$ is the symbol rate. A schematic representation of the transmitted signal is shown in Figure 3.

The signal $\tilde{E}\left(t,0\right)$ is transmitted over $N_{s}$ (homogeneous) fibre spans, each of length $L_{s}$ and each followed by an ideal lumped optical amplifier for which the gain exactly recovers from the span losses. Since in this work we are only concerned about the prediction of NLI arising from the signal–signal nonlinear interactions along the fibre propagation, the optical noise added by the amplifier plays no role in the model and will be entirely neglected. The signal at the channel output $\tilde{E}\left(t,N_{s},L_{s}\right)$ is ideally compensated for accumulated chromatic dispersion in the link (see Section 4). In the frequency-domain output of the chromatic dispersion compensation (CDC) block $\overset{˚}{E}\left(f,N_{s},L_{s}\right)$ (Figure 2), we ideally isolate the first-order RP term $E_{1}\left(f,N_{s},L_{s}\right)$ (see Section 4) and we compute its PSD $S(f,N_{s},L_{s})$. The vector of the NLI powers $\Sigma_{\mathrm{NLI}}≜{\left[\sigma_{\mathrm{NLI},x}^{2},\sigma_{\mathrm{NLI},y}^{2}\right]}^{T}$ for both *x* and *y* polarisations is obtained by integrating over the frequency interval $\left[-R_{s}/2,\;R_{s}/2\right]$ the NLI PSD weighted by the function ${\left|P\left(f\right)\right|}^{2}$, where $P^{*}\left(f\right)$ is the frequency response of a matched filter (MF) for the system under consideration. As shown in Figure 2, this quantity is equivalent to the variance of the output of the MF followed by symbol-rate sampling, which more naturally arises when assessing the transmission performance of systems employing an MF at the receiver. The model in this manuscript provides an analytical relationship between the statistical features of the transmitted symbols $a_{n}$ and $\Sigma_{\mathrm{NLI}}$.

### 3.2. DP-4D vs. PM-2D Formats

The model presented in this paper allows for prediction of the NLI for generic 4D real modulation formats. A 4D format is defined as a set

(2) $$A≜\{a^{\left(i\right)}={\left[a_{x}^{\left(i\right)},a_{y}^{\left(i\right)}\right]}^{T}\in C^{2},\;\;i=1,2,\ldots ,M\},$$

where $a_{x}$ and $a_{y}$ are the symbols modulated on two orthogonal polarisation states *x* and *y*, respectively, and *M* is the modulation cardinality. It can be seen that the elements in A are 2D vectors in C as opposed to 4D. This is only due the to baseband-equivalent representation of signals used throughout this paper, while it is common to refer to a modulation format dimensionality based on the real signal dimensions, which justifies the 4D format label.

Two important particular cases of the formats in (2) are (i) the so-called polarisation-multiplexed 2D (PM-2D) modulation formats, which are characterised by $A=X^{2}$, X∈C, where X represents the 2D component constellation, and (ii) polarisation-hybrid 2D modulation formats characterized by A=X×Y, with X,Y∈C, X≠Y, where X and Y are two distinct component 2D formats in *x* and *y* polarisation, respectively. PM-2D formats are the most common ones in optical communications due to their generation’s simplicity. Both PM-2D and polarisation-hybrid 2D formats are often analysed in terms of their 2D polarisation components. This is because A can be factorised in two component formats which are independently encoded. Hence, if the generic transmitted constellation point is regarded as a random variable, in a conventional PM-2D format, the two polarisation components are statistically independent. In the remainder of this paper, no specific assumption on either the geometry or the statistic of the transmitted 4D symbols will be made, except the zero-mean feature $E\{a^{\left(i\right)}\}=0.$

### 3.3. Transmitted Signal Form

Let $\tilde{E}\left(t,z\right)={\tilde{E}}_{x}\left(t,z\right)i_{x}+{\tilde{E}}_{y}\left(t,z\right)i_{y}$ be the complex envelope of the optical field vector at time *t* and fibre section *z*, and let $i_{x}$, $i_{y}$ denote 2 orthonormal polarisations of the transversal plane of propagation. Let also $E\left(f,z\right)=E_{x}\left(f,z\right)i_{x}+E_{y}\left(f,z\right)i_{y}$ be the (vector) Fourier transform of $\tilde{E}\left(t,z\right)$ defined as

$$E\left(f,z\right)=F\left\{\tilde{E}\left(t,z\right)\right\}≜\int_{-\infty }^{\infty }\tilde{E}\left(t,z\right)e^{-j2\pi ft}dt.$$

Because of the periodicity assumption made in (1) (see Figure 3), we can write $\tilde{E}\left(t,0\right)$ as

(3) $$\tilde{E}\left(t,0\right)=\sum_{k=-\infty }^{\infty }C_{k}\;e^{j2\pi k\Delta_{f}t},$$

where $C_{k}={\left[C_{x,k},C_{y,k}\right]}^{T}$, $C_{x/y,k}$ are the Fourier series coefficients of $\tilde{E}\left(t,0\right)$ and $\Delta_{f}=1/T$ is the frequency spacing of the spectral lines in $E_{x/y}\left(f,z\right)$. Hence, E(f,0) can be then written as

(4) $$\begin{array}{c} E\left(f,0\right)=\sum_{k=-\infty }^{\infty }C_{k}\;\delta \left(f-k\Delta_{f}\right). \end{array}$$

Since each component of $\tilde{E}\left(t,0\right)$ is periodic with period *T*, we can write

$$\tilde{E}\left(t,0\right)=\sum_{n=-\infty }^{\infty }\hat{E}\left(t-nT,0\right),$$

where, as per assumption in (1), we have

$$\hat{E}\left(t,0\right)≜\left\{\begin{array}{cc} \sum_{n=-(W-1)/2}^{(W-1)/2}a_{n}p\left(t-nT_{s}\right), & \mathrm{for}\;\;-\frac{T}{2}\leq t\leq \frac{T}{2}\\ 0, & \mathrm{otherwise} \end{array}\right.,$$

and, *W* is assumed to be odd without loss of generality. Under the above assumptions, the Fourier coefficients in (3), for k∈Z, are given by

(5a) $$\begin{array}{cc} C_{k} & =\Delta_{f}\int_{-\frac{T}{2}}^{\frac{T}{2}}\hat{E}\left(t,0\right)e^{-j2\pi k\Delta_{f}t}dt \end{array}$$

(5b) $$\begin{array}{cc} \; & =\Delta_{f}\int_{-\frac{T}{2}}^{\frac{T}{2}}\sum_{n=-(W-1)/2}^{(W-1)/2}a_{n}p\left(t-nT_{s}\right)e^{-j2\pi k\Delta_{f}t}dt \end{array}$$

(5c) $$\begin{array}{cc} \; & =\Delta_{f}\sum_{n=-(W-1)/2}^{(W-1)/2}a_{n}\int_{-\frac{T}{2}}^{\frac{T}{2}}p\left(t-nT_{s}\right)e^{-j2\pi k\Delta_{f}t}dt \end{array}$$

(5d) $$\begin{array}{cc} \; & \approx \Delta_{f}\sum_{n=-(W-1)/2}^{(W-1)/2}a_{n}P\left(k\Delta_{f}\right)e^{-j2\pi k\Delta_{f}nT_{s}} \end{array}$$

(5e) $$\begin{array}{cc} \; & =\Delta_{f}P\left(k\Delta_{f}\right)\sum_{n=-(W-1)/2}^{(W-1)/2}a_{n}e^{-j2\pi \frac{kn}{W}} \end{array}$$

(5f) $$\begin{array}{cc} \; & =\sqrt{\Delta_{f}}P\left(k\Delta_{f}\right)\nu_{k}, \end{array}$$

where P(f)≜F{p(t)} and

(6) $$\nu_{k}={\left[\nu_{x,k},\nu_{y,k}\right]}^{T}=\sqrt{\Delta_{f}}\sum_{n=-(W-1)/2}^{(W-1)/2}a_{n}e^{-j2\pi \frac{kn}{W}},\;\;\forall \;k\in Z,$$

are the discrete Fourier transforms of the sequence $a_{n}$, n=0,1,…,W−1. Note that the approximation in (5c)–(5d) is justified only for large enough values of *T* as

$$\lim_{T\to \infty }\int_{-\frac{T}{2}}^{\frac{T}{2}}p\left(t-nT_{s}\right)e^{-j2\pi k\Delta_{f}t}dt=F\left\{p\left(t-nT_{s}\right)\right\}{|}_{f=k\Delta_{f}},\;\mathrm{for}\;\;n,k\in Z,$$

and letting T→∞ will be the approach taken at a later stage in this derivation.

Finally, combining (4) and (5f), we obtain

(7) $$E\left(f,0\right)=\sqrt{\Delta_{f}}\sum_{k=-\infty }^{\infty }P\left(k\Delta_{f}\right)\;\nu_{k}\;\delta \left(f-k\Delta_{f}\right)\approx \sum_{k=-(W-1)/2}^{(W-1)/2}P\left(k\Delta_{f}\right)\;\nu_{k}\;\delta \left(f-k\Delta_{f}\right),$$

where the approximate equality on the right-hand side of (7) stems from the fact that p(t) is assumed to be strictly or quasi-strictly band-limited (see Section 3.1). Hence, $P(k\Delta_{f})$ is effectively equal to zero for k=−W/2,−W/2+1,…,W/2.

## 4. PSD of the First-Order NLI for Periodic Transmitted Signals

To find an analytical expression for NLI power, first, a solution as explicit as possible to the Manakov equation [12]

(8) $$\begin{array}{cc} \frac{\partial \tilde{E}\left(t,z\right)}{\partial z} & =-\frac{\alpha }{2}\tilde{E}\left(t,z\right)-j\frac{\beta_{2}}{2}\frac{\partial^{2}\tilde{E}\left(t,z\right)}{\partial t^{2}}+j\frac{8}{9}\gamma {\left|\tilde{E}\left(t,z\right)\right|}^{2}\tilde{E}\left(t,z\right), \end{array}$$

must be found. Equation (8) describes the propagation of the optical field $\tilde{E}\left(t,z\right)$ in a single strand of fibre (e.g., a fibre span with no amplifier in the system in Figure 2). In this case, α, $\beta_{2}$, and γ representing the attenuation, group velocity dispersion, and nonlinearity coefficients, respectively, can be assumed to be spatially constant. As it is well-known, general closed-form solutions are not available for (8). Like most of the existing NLI power models in the literature, the model derived here operates within a first-order perturbative framework. In particular, a frequency-domain first-order regular perturbation (RP) approach in the γ coefficient is performed [13,14], i.e., the Fourier transform of the solution in (8) is expressed as

(9) $$E\left(f,z\right)=\sum_{n=0}^{\infty }\gamma^{n}A_{n}\left(f,z\right)\approx A_{0}\left(f,z\right)+\gamma A_{1}\left(f,z\right),$$

where

(10) $$E_{n}\left(f,z\right)=\gamma^{n}A_{n}\left(f,z\right)\;\mathrm{for}\;\;n=0,1,\ldots ,$$

represents the so-called *n*th order term of the expansion.

In the following theorem, we present the expressions for $E_{0}\left(f,z\right),$ and $E_{1}\left(f,z\right),$ when a multiple fibre span system like the one in Figure 2 is considered. These expressions are well-known in the literature (see, e.g., [13]). Nevertheless, we present the proof in Appendix A for completeness.

**Theorem** **1** (First-order frequency-domain RP solution for a multi-span fibre system)**.** *Let E(f,z) be the solution in the frequency domain of the Manakov equation for the system in Figure 2 with initial condition at distance z=0 given by the transmitted signal E(f,0). Then, the first-order RP solution after $N_{s}$ spans $E(f,N_{s},L_{s})$ is given by*

$$E\left(f,N_{s},L_{s}\right)\approx E_{0}\left(f,N_{s},L_{s}\right)+E_{1}\left(f,N_{s},L_{s}\right),$$

*where the zeroth-order term is given by*

$$E_{0}\left(f,N_{s},L_{s}\right)=E_{0}\left(f,0\right)e^{j2\pi^{2}f^{2}\beta_{2}N_{s}L_{s}},$$

*and the first-order term is*

(11) $$E_{1}\left(f,N_{s},L_{s}\right)=-j\frac{8}{9}\gamma e^{j2\pi^{2}f^{2}\beta_{2}N_{s}L_{s}}\int_{-\infty }^{\infty }\int_{-\infty }^{\infty }E^{T}\left(f_{1},0\right)E^{*}\left(f_{2},0\right)E\left(f-f_{1}+f_{2},0\right)\eta \left(f_{1},f_{2},f,N_{s},L_{s}\right)df_{1}df_{2},$$

*with*

(12) $$\eta \left(f_{1},f_{2},f,N_{s},L_{s}\right)≜\frac{1-e^{-\alpha L_{s}}e^{j4\pi^{2}\beta_{2}\left(f-f_{1}\right)\left(f_{2}-f_{1}\right)L_{s}}}{\alpha -j4\pi^{2}\beta_{2}\left(f-f_{1}\right)\left(f_{2}-f_{1}\right)}\sum_{l=1}^{N_{s}}e^{-j4\pi^{2}\beta_{2}\left(l-1\right)\left(f-f_{1}\right)\left(f_{2}-f_{1}\right)L_{s}},$$

*where $N_{s}$ and $L_{s}$ are the number of spans and the span length of each span, respectively.*

**Proof.**  See Appendix A. □

While Theorem 1 gives an approximation for the field at the output of the fibre, we are interested in the field after ideal CDC (see Figure 2). Ideal CDC ideally removes the exponential $e^{j2\beta_{2}\pi^{2}f^{2}N_{s}L_{s}}$ from (11), leading to a first-order term in the RP solution for the system in Figure 2 given by

(13) $$\begin{array}{c} {\overset{˚}{E}}_{1}\left(f,N_{s},L_{s}\right)={\left[{\overset{˚}{E}}_{1,x},{\overset{˚}{E}}_{1,y}\right]}^{T}=-j\frac{8}{9}\gamma \int_{-\infty }^{\infty }\int_{-\infty }^{\infty }E^{T}\left(f_{1},0\right)E^{*}\left(f_{2},0\right)E\left(f-f_{1}+f_{2},0\right)\\ \cdot \eta \left(f_{1},f_{2},f,N_{s},L_{s}\right)df_{1}df_{2}. \end{array}$$

Substituting the spectrum of the transmitted periodic signal (7) in (13), we obtain, for instance, for the *x* component in (13),

(14) $$\begin{array}{cc} {\overset{˚}{E}}_{1,x}\left(f,N_{s},L_{s}\right) & =-j\frac{8}{9}\gamma \Delta_{f}^{3/2}\sum_{k=-\infty }^{\infty }\sum_{m=-\infty }^{\infty }\sum_{n=-\infty }^{\infty }P\left(k\Delta_{f}\right)P^{*}\left(m\Delta_{f}\right)P\left(n\Delta_{f}\right)\left(\nu_{x,k}\nu_{x,m}^{*}\nu_{x,n}+\nu_{y,k}\nu_{y,m}^{*}\nu_{x,n}\right)\\ \; & \cdot \int_{-\infty }^{\infty }\int_{-\infty }^{\infty }\delta \left(f_{1}-k\Delta_{f}\right)\delta \left(f_{2}-m\Delta_{f}\right)\delta \left(f-f_{1}+f_{2}-n\Delta_{f}\right)\eta \left(f_{1},f_{2},f,N_{s},L_{s}\right)df_{1}df_{2}. \end{array}$$

Although the product of Dirac’s deltas in (14) is not well-defined in the standard distribution theory framework, in this case, such product can be dealt with in the same way as products between distributions and smooth functions. This approach was formalised by Colombeau in his theory of product between distributions [15]. Thus, integrating in $f_{1}$ and $f_{2}$, we obtain

(15) $$\begin{array}{cc} {\overset{˚}{E}}_{1,x} & \left(f,N_{s},L_{s}\right)=-j\frac{8}{9}\gamma \Delta_{f}^{3/2}\sum_{k=-\infty }^{\infty }\sum_{m=-\infty }^{\infty }\sum_{n=-\infty }^{\infty }P\left(k\Delta_{f}\right)P^{*}\left(m\Delta_{f}\right)P\left(n\Delta_{f}\right)\\ \; & \cdot \left(\nu_{x,k}\nu_{x,m}^{*}\nu_{x,n}+\nu_{y,k}\nu_{y,m}^{*}\nu_{x,n}\right)\eta \left(k\Delta_{f},m\Delta_{f},\left(k-m+n\right)\Delta_{f},N_{s},L_{s}\right)\delta \left(f-\left(k-m+n\right)\Delta_{f}\right). \end{array}$$

Setting i=k−m+n and defining

(16) $$\begin{array}{cc} \eta_{k,m,n} & ≜\eta (k\Delta_{f},m\Delta_{f},\left(k-m+n\right)\Delta_{f},N_{s},L_{s})\\ \; & =\frac{1-e^{-\alpha L_{s}}e^{j4\pi^{2}\Delta_{f}^{2}\beta_{2}\left(n-m\right)\left(m-k\right)L_{s}}}{\alpha -j4\pi^{2}\Delta_{f}^{2}\beta_{2}\left(n-m\right)\left(m-k\right)}\sum_{l=1}^{N_{s}}e^{-j4\pi^{2}\Delta_{f}^{2}\beta_{2}\left(l-1\right)\left(n-m\right)\left(m-k\right)L_{s}} \end{array}$$

(15) can be rewritten as

(17) $${\overset{˚}{E}}_{1,x}\left(f,N_{s},L_{s}\right)=\sum_{i=-\infty }^{\infty }c_{i}\delta \left(f-i\Delta_{f}\right),$$

where

(18) $$c_{i}≜-j\frac{8}{9}\gamma \Delta_{f}^{3/2}\sum_{\begin{array}{c} \left(k,m,n\right)\in S_{i} \end{array}}P\left(k\Delta_{f}\right)P^{*}\left(m\Delta_{f}\right)P\left(n\Delta_{f}\right)\left(\nu_{x,k}\nu_{x,m}^{*}\nu_{x,n}+\nu_{y,k}\nu_{y,m}^{*}\nu_{x,n}\right)\eta_{k,m,n},$$

and

(19) $$S_{i}≜\left\{\left(k,m,n\right)\in Z^{3}:k-m+n=i\right\}.$$

The PSD of the received nonlinear interference (to the 1st-order) is defined as

$$S\left(f,N_{s},L_{s}\right)={\left[S_{x}\left(f,N_{s},L_{s}\right),S_{y}\left(f,N_{s},L_{s}\right)\right]}^{T}≜{\left[E\left\{|{\overset{˚}{E}}_{1,x}\left(f,N_{s},L_{s}\right){|}^{2}\right\},E\left\{|{\overset{˚}{E}}_{1,y}\left(f,N_{s},L_{s}\right){|}^{2}\right\}\right]}^{T}.$$

For periodic signals, which in the frequency domain can be expressed as in (17), the PSD can be expressed as [16] (Section 4.1.2)

(20) $$S_{x}\left(f,N_{s},L_{s}\right)=\sum_{i=-\infty }^{\infty }E\{|c_{i}{|}^{2}\}\delta \left(f-i\Delta_{f}\right).$$

Substituting the expression (18) for $c_{i}$ in (20), we obtain

$$\begin{array}{cc} S_{x}\left(f,N_{s},L_{s}\right) & ={\left(\frac{8}{9}\right)}^{2}\gamma^{2}\Delta_{f}^{3}\sum_{i=-\infty }^{\infty }\delta \left(f-i\Delta_{f}\right)E\{\sum_{\left(k,m,n\right)\in S_{i}}P\left(k\Delta_{f}\right)P^{*}\left(m\Delta_{f}\right)P\left(n\Delta_{f}\right)\left(\nu_{x,k}\nu_{x,m}^{*}\nu_{x,n}\right. \end{array}$$

(21a) $$\begin{array}{cc} \left.+\nu_{y,k}\nu_{y,m}^{*}\nu_{x,n}\right) & \eta_{k,m,n}\sum_{\left(k^{\prime },m^{\prime },n^{\prime }\right)\in S_{i}}P^{*}\left(k^{\prime }\Delta_{f}\right)P\left(m^{\prime }\Delta_{f}\right)P^{*}\left(n^{\prime }\Delta_{f}\right)\left(\nu_{x,k^{\prime }}^{*}\nu_{x,m^{\prime }}\nu_{x,n^{\prime }}^{*}+\nu_{y,k^{\prime }}^{*}\nu_{y,m^{\prime }}\nu_{x,n^{\prime }}^{*}\right)\eta_{k^{\prime },m^{\prime },n^{\prime }}^{*}\}\\ \; & ={\left(\frac{8}{9}\right)}^{2}\gamma^{2}\Delta_{f}^{3}\sum_{i=-\infty }^{\infty }\delta \left(f-i\Delta_{f}\right)E\{\sum_{\begin{array}{c} \left(k,m,n\right)\in S_{i}\\ \left(k^{\prime },m^{\prime },n^{\prime }\right)\in S_{i} \end{array}}P_{k,m,n,k^{\prime },m^{\prime },n^{\prime }}\left(\nu_{x,k}\nu_{x,m}^{*}\nu_{x,n}\nu_{x,k^{\prime }}^{*}\nu_{x,m^{\prime }}\nu_{x,n^{\prime }}^{*}\right. \end{array}$$

(21b) $$+\nu_{x,k}\nu_{x,m}^{*}\nu_{x,n}\nu_{y,k^{\prime }}^{*}\nu_{y,m^{\prime }}\nu_{x,n^{\prime }}^{*}+\nu_{y,k}\nu_{y,m}^{*}\nu_{x,n}\nu_{x,k^{\prime }}^{*}\nu_{x,m^{\prime }}\nu_{x,n^{\prime }}^{*}+\left.\nu_{y,k}\nu_{y,m}^{*}\nu_{x,n}\nu_{y,k^{\prime }}^{*}\nu_{y,m^{\prime }}\nu_{x,n^{\prime }}^{*}\right)\eta_{k,m,n}\eta_{k^{\prime },m^{\prime },n^{\prime }}^{*}\},$$

where we have defined

(22) $$P_{k,m,n,k^{\prime },m^{\prime },n^{\prime }}≜P\left(k\Delta_{f}\right)P^{*}\left(m\Delta_{f}\right)P\left(n\Delta_{f}\right)P^{*}\left(k^{\prime }\Delta_{f}\right)P\left(m^{\prime }\Delta_{f}\right)P^{*}\left(n^{\prime }\Delta_{f}\right).$$

The following proposition can be used to make (21b) more compact, and in particular, it will be used to group the two inner correlation terms in (21b) ($\nu_{x,k}\nu_{x,m}^{*}\nu_{x,n}\nu_{y,k^{\prime }}^{*}\nu_{y,m^{\prime }}\nu_{x,n^{\prime }}^{*}$ and $\nu_{y,k}\nu_{y,m}^{*}\nu_{x,n}\nu_{x,k^{\prime }}^{*}\nu_{x,m^{\prime }}\nu_{x,n^{\prime }}^{*}$).

**Proposition** **1.** *For $P_{k,m,n,k^{\prime },m^{\prime },n^{\prime }}$ in (22), we have*

(23) $$\begin{array}{cc} \; & \sum_{\begin{array}{c} \left(k,m,n\right)\in S_{i}\\ \left(k^{\prime },m^{\prime },n^{\prime }\right)\in S_{i} \end{array}}P_{k,m,n,k^{\prime },m^{\prime },n^{\prime }}\nu_{x,k}\nu_{x,m}^{*}\nu_{x,n}\nu_{y,k^{\prime }}^{*}\nu_{y,m^{\prime }}\nu_{x,n^{\prime }}^{*}\eta_{k,m,n}\eta_{k^{\prime },m^{\prime },n^{\prime }}^{*}\\ \; & =(\sum_{\begin{array}{c} \left(k,m,n\right)\in S_{i}\\ \left(k^{\prime },m^{\prime },n^{\prime }\right)\in S_{i} \end{array}}P_{k,m,n,k^{\prime },m^{\prime },n^{\prime }}\nu_{y,k}\nu_{y,m}^{*}\nu_{x,n}\nu_{x,k^{\prime }}^{*}\nu_{x,m^{\prime }}\nu_{x,n^{\prime }}^{*}\eta_{k,m,n}\eta_{k^{\prime },m^{\prime },n^{\prime }}^{*}{)}^{*}. \end{array}$$

**Proof.**  See Appendix B. □

Using (23) and (21b) can be written as

(24) Sx(f,Ns,Ls)=892γ2Δf3∑i=−∞∞δ(f−iΔf)E{∑(k,m,n)∈Si(k′,m′,n′)∈SiPk,m,n,k′,m′,n′·νx,kνx,m∗νx,nνx,k′∗νx,m′νx,n′∗+νy,kνy,m∗νx,nνy,k′∗νy,m′νx,n′∗ηk,m,nηk′,m′,n′∗+2Re{Pk,m,n,k′,m′,n′νx,kνx,m∗νx,nνy,k′∗νy,m′νx,n′∗ηk,n,mηk′,n′,m′∗}}=892γ2Δf3∑i=−∞∞δ(f−iΔf)∑(k,m,n)∈Si(k′,m′,n′)∈SiPk,m,n,k′,m′,n′Eνx,kνx,m∗νx,nνx,k′∗νx,m′νx,n′∗+Eνy,kνy,m∗νx,nνy,k′∗νy,m′νx,n′∗ηk,m,nηk′,m′,n′∗+2Re{Pk,m,n,k′,m′,n′Eνx,kνx,m∗νx,nνy,k′∗νy,m′νx,n′∗ηk,n,mηk′,n′,m′∗},

where the real part operator arises from the sum of the complex conjugate terms discussed in the Proposition section (Section 1).

According to (24), calculation of the PSD of the NLI reduces to the computation of a four-dimensional summation (per frequency component $i\Delta_{f}$) of three sixth-order correlations of the sequence of random variables $\nu_{x/y,n},\;n=0,1,\ldots ,W-1$. The *y*-component $S_{y}\left(f,N_{s},L_{s}\right)$ of the PSD can be calculated once $S_{x}\left(f,N_{s},L_{s}\right)$ is obtained by simply swapping the polarisation labels x→y and y→x. This is due to the invariance of the Manakov equation in (8) to such a transformation.

## 5. Classification of the Modulation-Dependent Contributions in the 6th-Order Frequency-Domain Correlation

In this section, we will break down the frequency-domain sixth-order correlation terms in (24) to highlight different contributions in terms of 4D modulation-dependent cross-polarisation correlations.

### 5.1. Expansion in Terms of the Stochastic Moments of the Transmitted Modulation Format

To relate the PSD in (24) to the statistical properties of the transmitted modulation format, we replace (6) into (24), obtaining

(25) Sx(f,Ns,Ls)=892γ2Δf3∑i=−∞∞δ(f−iΔf)∑(k,m,n)∈Si(k′,m′,n′)∈Si[Pk,m,n,k′,m′,n′ηk,m,nηk′,m′,n′∗·∑i∈{0,1,…,W−1}6Si(k,m,n,k′,m′,n′)+2Re{Pk,m,n,k′,m′,n′ηk,m,nηk′,m′,n′∗·∑i∈{0,1,…,W−1}6Ti(k,m,n,k′,m′,n′)}],

where $i≜(i_{1},i_{2},\ldots ,i_{6})$,

(26) $$\begin{array}{c} \begin{array}{cc} S_{i}\left(k,m,n,k^{\prime },m^{\prime },n^{\prime }\right) & ≜\Delta_{f}^{3}\left[E\left\{a_{x,i_{1}}a_{x,i_{2}}^{*}a_{x,i_{3}}a_{x,i_{4}}^{*}a_{x,i_{5}}a_{x,i_{6}}^{*}\right\}+E\left\{a_{y,i_{1}}a_{y,i_{2}}^{*}a_{x,i_{3}}a_{y,i_{4}}^{*}a_{y,i_{5}}a_{x,i_{6}}^{*}\right\}\right]\\ \; & \cdot e^{-j\frac{2\pi }{W}\left(ki_{1}-mi_{2}+ni_{3}-k^{\prime }i_{4}+m^{\prime }i_{5}-n^{\prime }i_{6}\right)}, \end{array} \end{array}$$

and

(27) $$\begin{array}{c} \begin{array}{cc} \; & T_{i}\left(k,m,n,k^{\prime },m^{\prime },n^{\prime }\right)≜\Delta_{f}^{3}E\left\{a_{x,i_{1}}a_{x,i_{2}}^{*}a_{x,i_{3}}a_{y,i_{4}}^{*}a_{y,i_{5}}a_{x,i_{6}}^{*}\right\}e^{-j\frac{2\pi }{W}\left(ki_{1}-mi_{2}+ni_{3}-k^{\prime }i_{4}+m^{\prime }i_{5}-n^{\prime }i_{6}\right)}. \end{array} \end{array}$$

The terms $S_{i}\left(k,m,n,k^{\prime },m^{\prime },n^{\prime }\right)$ and $T_{i}\left(k,m,n,k^{\prime },m^{\prime },n^{\prime }\right)$ give rise to several correlations among the transmitted symbols $a_{x,i}$ and $a_{y,j}$ at different time-slots i,j, each weighted by a complex exponential. As discussed in Section 3, in this work, we operate under the assumption that the sequence of vector RVs $a_{i}$ for i=0,1,…,W−1 are independent, identically distributed (i.i.d.), and with $E\left\{a_{i}\right\}=E\left\{a\right\}=0$. As shown in the following example, this assumption allows us to discard the $S_{i}\left(k,m,n,k^{\prime },m^{\prime },n^{\prime }\right)$ and $T_{i}\left(k,m,n,k^{\prime },m^{\prime },n^{\prime }\right)$ terms which are identically zero for some values of i. Moreover, as it will be shown in Example 2 for all other values of i, $S_{i}\left(k,m,n,k^{\prime },m^{\prime },n^{\prime }\right)$, and $T_{i}\left(k,m,n,k^{\prime },m^{\prime },n^{\prime }\right)$ can be expressed as a product of high-order statistical moments of the RVs $a_{x}$ and $a_{y}$, which enables a more compact expression for (25).

**Example** **1.** *Under the i.i.d. assumption for the sequence of vector RVs $a_{i}$, i=0,1,…W−1 made in this work, in any of the cases where*

(28) $$i_{\kappa_{1}}\neq i_{\kappa_{2}}=i_{\kappa_{3}}=\ldots =i_{\kappa_{6}}\;\mathrm{for}\;\kappa_{1},\kappa_{2},\ldots ,\kappa_{6}=1,2,\ldots ,6;\;\;\kappa_{1}\neq \kappa_{2}\neq \ldots \neq \kappa_{6},$$

*any of the sixth-order correlations in (26) and (27) degenerate into a product between a first-order moment and a fifth-order correlation. Such a product is equal to zero under our assumption $E\left\{a_{x,i}\right\}=E\left\{a_{x}\right\}=0$. For example, for $i_{1}\neq i_{2}=i_{3}=\ldots =i_{6}$, we have*

$$\begin{array}{c} \begin{array}{cc} \; & E\left\{a_{x,i_{1}}a_{x,i_{2}}^{*}a_{x,i_{3}}a_{x,i_{4}}^{*}a_{x,i_{5}}a_{x,i_{6}}^{*}\right\}=E\left\{a_{x,i_{1}}\right\}E\{|a_{x,i_{2}}{|}^{4}a_{x,i_{2}}^{*}\}=E\left\{a_{x}\right\}E\{|a_{x}{|}^{4}a_{x}^{*}\}=0. \end{array} \end{array}$$

**Figure 4:**
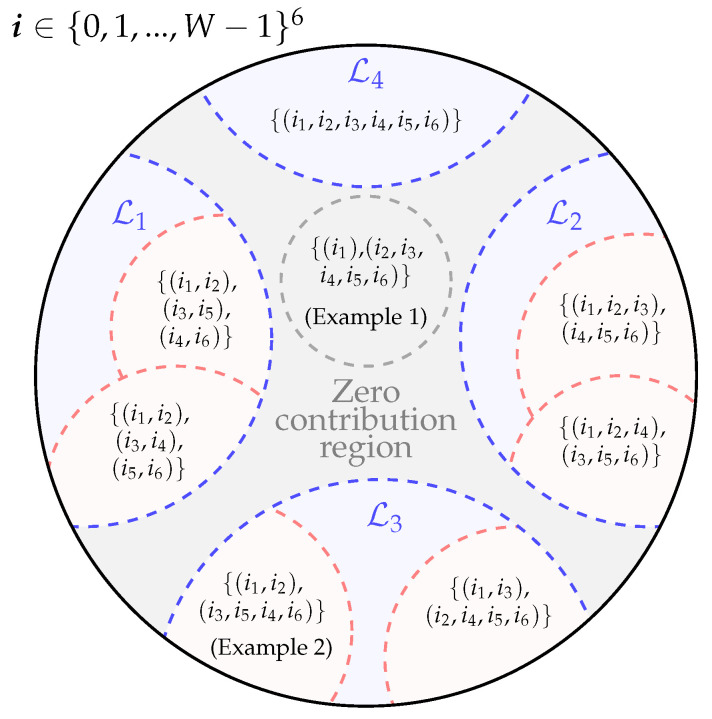
Venn diagram of the partition on the 6D space $i=\left(i_{1},i_{2},i_{3},i_{4},i_{5},i_{6}\right)\in {\left\{0,1,\ldots ,W-1\right\}}^{6}$ discussed in Section 5.2

*From this follows that, for all elements in the set defined in (28), $S_{i}\left(k,m,n,k^{\prime },m^{\prime },n^{\prime }\right)=0$ and $T_{i}\left(k,m,n,k^{\prime },m^{\prime },n^{\prime }\right)=0$. This example highlights a* zero-contribution *region in the 6D space* ${\left\{0,1,\ldots ,W-1\right\}}^{6}$*, as illustrated in Figure 4.*

The $S_{i}\left(k,m,n,k^{\prime },m^{\prime },n^{\prime }\right)$ and $T_{i}\left(k,m,n,k^{\prime },m^{\prime },n^{\prime }\right)$ contributions for the set in (28) are identically zero regardless of the values taken by $k,m,n,k^{\prime },m^{\prime }$, and $n^{\prime }$. However, as it will be shown in Section 6, for a specific subset of values $k,m,n,k^{\prime },m^{\prime }$, and $n^{\prime }$, such contributions cancel each other in the inner sums in (25) due to the complex exponential weights.

**Example** **2.** *Under the i.i.d. assumption for the sequence of vector RVs $a_{i}$, i=0,1,…W−1 made in this work, we have that, for all elements in the set $\left\{i\in {\left\{0,1,\ldots ,W-1\right\}}^{6},i_{1}=i_{2},i_{3}=i_{4}=i_{5}=i_{6},i_{1}\neq i_{3}\right\}$*

$$\begin{array}{cc} S_{i}\left(k,m,n,k^{\prime },m^{\prime },n^{\prime }\right) & =\Delta_{f}^{3}\left[E\{\right|a_{x,i_{1}}{|}^{2}\}E\{|a_{x,i_{3}}{|}^{4}\}+E\{|a_{y,i_{1}}{|}^{2}\}E\{|a_{x,i_{3}}{|}^{2}|a_{y,i_{3}}{|}^{2}\}]\\ \; & \cdot e^{-j\frac{2\pi }{W}\left(\left(k-m\right)i_{1}+\left(n-k^{\prime }+m^{\prime }-n^{\prime }\right)i_{3}\right)}\\ \; & =\Delta_{f}^{3}\left[E\{\right|a_{x}{|}^{2}\}E\{|a_{x}{|}^{4}\}+E\{|a_{y}{|}^{2}\}E\{|a_{x}{|}^{2}|a_{y}{|}^{2}\}]e^{-j\frac{2\pi }{W}\left(\left(k-m\right)i_{1}+\left(n-k^{\prime }+m^{\prime }-n^{\prime }\right)i_{3}\right)},\\ T_{i}\left(k,m,n,k^{\prime },m^{\prime },n^{\prime }\right) & =\Delta_{f}^{3}E\{|a_{x,i_{1}}{|}^{2}\}E\{|a_{x,i_{3}}{|}^{2}|a_{y,i_{3}}{|}^{2}\}e^{-j\frac{2\pi }{W}\left(ki_{1}-mi_{2}+ni_{3}-k^{\prime }i_{4}+m^{\prime }i_{5}-n^{\prime }i_{6}\right)}\\ \; & =\Delta_{f}^{3}E\{|a_{x}{|}^{2}\}E\{|a_{x}{|}^{2}|a_{y}{|}^{2}\}e^{-j\frac{2\pi }{W}\left(ki_{1}-mi_{2}+ni_{3}-k^{\prime }i_{4}+m^{\prime }i_{5}-n^{\prime }i_{6}\right)}. \end{array}$$

*It can be noted that (i) the sixth-order correlation degenerates into a product of marginal high-order moments of $a_{x}$ and $a_{y}$ and into the cross-polarisation correlation $E\{|a_{x}{|}^{2}|a_{y}{|}^{2}\}$ and (ii) all elements within the set in this example contribute to the inner summation in (25) with the same set of moments, cross-polarisation correlations, and products thereof (i.e., $E\{|a_{x}{|}^{2}\},E\{|a_{x}{|}^{4}\},E\{|a_{y}{|}^{2}\},andE\{|a_{x}{|}^{2}|a_{y}{|}^{2}\}$). This example illustrates how to break down each instance of the contributions $S_{i}\left(k,m,n,k^{\prime },m^{\prime },n^{\prime }\right)$ and $T_{i}\left(k,m,n,k^{\prime },m^{\prime },n^{\prime }\right)$, which will be then added up in Section 6.*

In the remainder of this section, we first partition the six-dimensional space $i\in {\left\{0,1,\ldots ,W-1\right\}}^{6}$ and list all sets corresponding to nonzero elements of $S_{i}\left(k,m,n,k^{\prime },m^{\prime },n^{\prime }\right)$ and $T_{i}\left(k,m,n,k^{\prime },m^{\prime },n^{\prime }\right)$. As shown in Example 2, this will help highlight the contribution of a specific set in terms of high-order moments of the transmitted symbols a in (25). Then, we proceed to list all such contributions.

### 5.2. Set Partitioning

The six-dimensional space $i\in {\left\{0,1,\ldots ,W-1\right\}}^{6}$ can be partitioned in different *subsets*, each one uniquely defined by a partition on the set of indices ($i_{1}$, $i_{2}$, $i_{3}$, $i_{4}$, $i_{5}$, and $i_{6}$). Each partition defines its corresponding subset in ${\left\{0,1,\ldots ,W-1\right\}}^{6}$ as follows: for each index partition, the indices belonging to the same subset all take the same value, whilst the indices belonging to different subsets have distinct values. This is schematically illustrated in Figure 4. For example, the subset of ${\left\{0,1,\ldots ,W-1\right\}}^{6}$ labelled by the index partition $\left\{\left(i_{1},i_{2}\right),\left(i_{3},i_{4}\right),\left(i_{5},i_{6}\right)\right\}$ is defined as $\left\{i\in {\left\{0,1,\ldots ,W-1\right\}}^{6}:i_{1}=i_{2},i_{3}=i_{4},i_{5}=i_{6},i_{1}\neq i_{3}\neq i_{5}\right\}$. This subset is shown in Figure 4 as part of $L_{1}$.

In Figure 4, the *families of subsets* of ${\left\{0,1,\ldots ,W-1\right\}}^{6}$ labelled $L_{i}$, i=1,2,…,4, are also highlighted. These families are characterised by subsets sharing the same cardinality of elements associated to their corresponding index partition. For example, in $L_{1}$, all index partitions are characterised by 3 subsets, each one containing 2 indices. As shown in Example 2, this way of partitioning the set ${\left\{0,1,\ldots ,W-1\right\}}^{6}$ is useful as it separates out the different contributions of (25) based on the high-order moments of a, as it is highlighted in region $L_{3}$ of Figure 4.

Since we have 6 different indices, the number of subsets in a partition can vary from 1 to 6. Each of these subsets can contain a number of elements also ranging from 1 to 6. However, the subsets of ${\left\{0,1,\ldots ,W-1\right\}}^{6}$, where the corresponding index partition has one or more index subsets with only one element, bring no contribution to (25) and thus can be discarded. This is illustrated in Example 1. The above class of index partitions then forms a *zero contribution* region, as shown in Figure 4. Such a region also includes all subsets where the corresponding index partitions contain 4 or more index subsets, as at least one of these subsets will have to contain only one element.

As shown in Figure 4, by removing the *zero contribution* region from ${\left\{0,1,\ldots ,W-1\right\}}^{6}$, only 4 different families of subsets are left:

(i) $L_{1}=\left\{i\in {\left\{0,1,\ldots ,W-1\right\}}^{6}:i_{\kappa_{1}}=i_{\kappa_{2}},\;i_{\kappa_{3}}=i_{\kappa_{4}},\;i_{\kappa_{5}}=i_{\kappa_{6}};\;\kappa_{1},\kappa_{2},\ldots \kappa_{6}=1,2,\ldots ,6;\;\kappa_{1}\neq \kappa_{2}\neq \kappa_{3}\neq \kappa_{4}\neq \kappa_{5}\neq \kappa_{6}\right\}$. This set contains all sets of elements where the indices $i_{1},i_{2},\ldots ,i_{6}$, can be grouped in 3 pairs. The indices take up the same value within each pair but different values across different pairs. It can be found that this set can be partitioned in 15 different subsets $C_{1}^{\left(i\right)},i=1,2,\ldots ,15$, representing all possible distinct ways of pairing the $i_{k}$ indices for k=1,2,…6. These sets are listed in Table A1 in Appendix C, where each column shows a subgroup of indices taking the same value;
(ii) $L_{2}:\left\{i\in {\left\{0,1,\ldots ,W-1\right\}}^{6},\;i_{\kappa_{1}}=i_{\kappa_{2}}=i_{\kappa_{3}},\;i_{\kappa_{4}}=i_{\kappa_{5}}=i_{\kappa_{6}};\;\kappa_{1},\kappa_{2},\ldots ,\kappa_{6}=1,2,\ldots ,6;\;\kappa_{1}\neq \kappa_{2}\neq \kappa_{3}\neq \kappa_{4}\neq \kappa_{5}\neq \kappa_{6}\right\}$ which can be broken down in 10 subsets $C_{2}^{\left(i\right)},\;i=1,2,\ldots ,10$, listed in Table A2 in Appendix C. Each index subgroup identifies a triplet of indices assuming the same value;
(iii) $L_{3}=\left\{i\in {\left\{0,1,\ldots ,W-1\right\}}^{6}:i_{\kappa_{1}}=i_{\kappa_{2}},\;i_{\kappa_{3}}=i_{\kappa_{4}}=i_{\kappa_{5}}=i_{\kappa_{6}};\;\;\kappa_{1},\kappa_{2},\ldots ,\kappa_{6}=1,2,\ldots ,6,\kappa_{1}\neq \kappa_{2}\neq \kappa_{3}\neq \kappa_{4}\neq \kappa_{5}\neq \kappa_{6}\right\}$ which can be partitioned in 15 subsets $C_{3}^{\left(i\right)},\;i=1,2,\ldots ,15$, listed in Table A3 in Appendix C. Each of the two index subgroups identifies the pair and the quadruple of indices assuming the same value;
(iv) $L_{4}:\left\{i\in {\left\{0,1,\ldots ,W-1\right\}}^{6}:i_{1}=i_{2}=i_{3}=i_{4}=i_{5}=i_{6}\right\}$.

## 6. Evaluation of the L-Based Contributions

In this section, we provide three examples for the computation of the contributions of a generic element in $L_{1}$, $L_{2}$, and $L_{3}$. The full list of contributions in these three sets and the ones in $L_{4}$ are given in Section 6.1, Section 6.2, Section 6.3, Section 6.4.

We label each contribution as $M_{g}^{\left(h\right)}\left(k,m,n,k^{\prime },m^{\prime },n^{\prime }\right)$ and $N_{g}^{\left(h\right)}\left(k,m,n,k^{\prime },m^{\prime },n^{\prime }\right)$, where

(29) $$\begin{array}{cc} M_{g}^{\left(h\right)}\left(k,m,n,k^{\prime },m^{\prime },n^{\prime }\right) & ≜\sum_{i\in C_{g}^{\left(h\right)}}S_{i}\left(k,m,n,k^{\prime },m^{\prime },n^{\prime }\right),\\ N_{g}^{\left(h\right)}\left(k,m,n,k^{\prime },m^{\prime },n^{\prime }\right) & ≜\sum_{i\in C_{g}^{\left(h\right)}}T_{i}\left(k,m,n,k^{\prime },m^{\prime },n^{\prime }\right), \end{array}$$

and the subsets $C_{g}^{\left(h\right)}$ are taken from Table A1, Table A2, Table A3 in Appendix C.

**Example** **3** (Contributions in $L_{1}$)**.** *$M_{1}^{\left(1\right)}$, i.e., one of the 2 contributions for the set $C_{1}^{\left(1\right)}=\left\{i\in {\left\{0,1,\ldots ,W-1\right\}}^{6}:i_{1}=i_{2},\;i_{3}=i_{4},\;i_{5}=i_{6},i_{1}\neq i_{3},\;i_{1}\neq i_{5},\;i_{3}\neq i_{5}\right\}$ is given by*

(30) $$\begin{array}{c} \begin{array}{cc} M_{1}^{\left(1\right)} & ≜\sum_{i\in C_{1}^{\left(1\right)}}S_{i}\left(k,m,n,k^{\prime },m^{\prime },n^{\prime }\right)\\ \; & =\Delta_{f}^{3}\left[E^{3}\left\{|a_{x}{|}^{2}\right\}+E\left\{|a_{y}{|}^{2}\right\}{\left|E\left\{a_{x}a_{y}^{*}\right\}\right|}^{2}\right]\sum_{i_{1}=0}^{W-1}e^{-j\frac{2\pi }{W}\left(k-m\right)i_{1}}\sum_{i_{3}\neq i_{1}}e^{-j\frac{2\pi }{W}\left(n-k^{\prime }\right)i_{3}}\\ \; & \cdot \sum_{\begin{array}{c} i_{5}\neq i_{1},\\ i_{5}\neq i_{3} \end{array}}e^{-j\frac{2\pi }{W}\left(m^{\prime }-n^{\prime }\right)i_{5}}. \end{array} \end{array}$$

*Since*

(31) $$\sum_{k=0}^{W-1}e^{jnk\frac{2\pi }{W}}=\left\{\begin{array}{cc} W, & \mathrm{for}\;\;n=pW,\;p\in Z\\ 0, & \mathrm{elsewhere} \end{array}\right.,$$

*we can compute (30) using the following approach:*

1. we add up the terms for all $i_{1},i_{3},i_{5}$ values including all cases when $i_{1}$, $i_{3}$, and $i_{5}$ are equal among each other. Because of (31), these terms sum up to $W^{3}$ only when $k=m+pW,\;n=k^{\prime }+pW,\;andm^{\prime }=n^{\prime }+pW$, p∈Z; otherwise, they sum to 0;
2. we subtract the terms corresponding to the cases: $i_{1}=i_{3},\;i_{1}\neq i_{5}$; $i_{1}=i_{5},\;i_{1}\neq i_{3}$; and $i_{3}=i_{5},\;i_{1}\neq i_{3}$. As an example, the number of terms defined by $i_{1}=i_{3},\;i_{1}\neq i_{5}$ is given by the difference between the number of all pairs $i_{1},i_{5}\in \left\{0,1,2,\ldots ,W-1\right\}$ and the number of terms for $i_{1}=i_{5}$. According to (31), the former terms sum to $W^{2}$ only for $k-m+n-k^{\prime }=pW,\;m^{\prime }-n^{\prime }=pW$, whereas the latter sum to W only for $k-m+n-k^{\prime }+m^{\prime }-n^{\prime }=pW$, with p∈Z. In all other cases, they all bring zero contribution. Similar results are obtained for $i_{1}=i_{5},\;i_{1}\neq i_{3}$ and $i_{3}=i_{5},\;i_{1}\neq i_{3}$;
3. we finally subtract the terms $i_{1}=i_{3}=i_{5}$, which sum to W only for $k-m+n-k^{\prime }+m^{\prime }-n^{\prime }=pW$, p∈Z; otherwise, they sum to 0 (see (31)).

*Hence, we obtain*

$$\begin{array}{cc} M_{1}^{\left(1\right)} & =\Delta_{f}^{3}[E^{3}\left\{\right|a_{x}{|}^{2}\}+2E^{2}\left\{\right|a_{x}{|}^{2}\}|E\left\{a_{x}a_{y}^{*}\right\}{|}^{2}+E\{|a_{y}{|}^{2}\}|E\left\{a_{x}a_{y}^{*}\right\}{|}^{2}][W^{3}\delta_{k-m-pW}\delta_{n-k^{\prime }-pW}\delta_{m^{\prime }-n^{\prime }-pW}\\ & -[W^{2}\left(\delta_{k-m+n-k^{\prime }-pW}\delta_{m^{\prime }-n^{\prime }-pW}+\delta_{k-m+m^{\prime }-n^{\prime }-pW}\delta_{n-k^{\prime }-pW}+\delta_{m^{\prime }-n^{\prime }+n-k^{\prime }-pW}\delta_{k-m-pW}\right)\\ \; & -3W\delta_{k-m+m^{\prime }-n^{\prime }+n-k^{\prime }-pW}]-W\delta_{k-m+m^{\prime }-n^{\prime }+n-k^{\prime }-pW}]\\ \; & =[E^{3}\left\{\right|a_{x}{|}^{2}\}+2E^{2}\left\{\right|a_{x}{|}^{2}\}|E\left\{a_{x}a_{y}^{*}\right\}{|}^{2}+E\{|a_{y}{|}^{2}\}|E\left\{a_{x}a_{y}^{*}\right\}{|}^{2}][R_{s}^{3}\delta_{k-m+pW}\delta_{n-k^{\prime }-pW}\delta_{m^{\prime }-n^{\prime }-pW}\\ \; & -R_{s}^{2}\Delta_{f}\left(\delta_{k-m+n-k^{\prime }-pW}\delta_{m^{\prime }-n^{\prime }-pW}+\delta_{k-m+m^{\prime }-n^{\prime }-pW}\delta_{n-k^{\prime }-pW}+\delta_{m^{\prime }-n^{\prime }+n-k^{\prime }-pW}\delta_{k-m-pW}\right)\\ \; & +2R_{s}\Delta_{f}^{2}\delta_{k-m+m^{\prime }-n^{\prime }+n-k^{\prime }-pW}], \end{array}$$

*where we have used $R_{s}=W\Delta_{f}$.*

*The same approach can be followed to compute $N_{1}^{\left(1\right)}$, which is, thus, given by*

$$\begin{array}{cc} N_{1}^{\left(1\right)} & ≜\sum_{i\in C_{1}^{\left(1\right)}}T_{i}\left(k,m,n,k^{\prime },m^{\prime },n^{\prime }\right)=E^{2}\left\{\right|a_{x}{|}^{2}\}|E\left\{a_{x}a_{y}^{*}\right\}{|}^{2}[R_{s}^{3}\delta_{k-m-pW}\delta_{n-k^{\prime }-pW}\delta_{m^{\prime }-n^{\prime }-pW}\\ & -R_{s}^{2}\Delta_{f}\left(\delta_{k-m+n-k^{\prime }-pW}\delta_{m^{\prime }-n^{\prime }-pW}+\delta_{k-m+m^{\prime }-n^{\prime }-pW}\delta_{n-k^{\prime }-pW}+\delta_{m^{\prime }-n^{\prime }+n-k^{\prime }-pW}\delta_{k-m-pW}\right)\\ \; & +2R_{s}\Delta_{f}^{2}\delta_{k-m+m^{\prime }-n^{\prime }+n-k^{\prime }-pW}]. \end{array}$$

*All other contributions in $L_{1}$ can be computed using the approach used in this example.*

**Example** **4** (Contributions in $L_{2}$)**.** *$M_{2}^{\left(1\right)}$, i.e., the contribution for the set $C_{2}^{\left(1\right)}=\left\{i\in {\left\{0,1,\ldots ,W-1\right\}}^{6}:i_{1}=i_{2}=i_{3},\;i_{4}=i_{5}=i_{6},\;i_{1}\neq i_{4}\right\}$ is given by*

(32) $$\begin{array}{c} \begin{array}{cc} M_{2}^{\left(1\right)} & =\sum_{i\in C_{2}^{\left(1\right)}}S_{i}\left(k,m,n,k^{\prime },m^{\prime },n^{\prime }\right)\\ \; & =\Delta_{f}^{3}[E\{a_{x,i_{1}}|a_{x,i_{1}}{|}^{2}\}E^{*}\{a_{x,i_{4}}|a_{x,i_{4}}{|}^{2}\}+E\{a_{x,i_{1}}|a_{y,i_{1}}{|}^{2}\}E^{*}\{a_{x,i_{4}}|a_{y,i_{4}}{|}^{2}\}]\\ \; & \cdot \sum_{i_{1}=0}^{W-1}e^{-j\frac{2\pi }{W}\left(k-m+n\right)i_{1}}\sum_{i_{4}\neq i_{1}}e^{-j\frac{2\pi }{W}\left(-k^{\prime }+m^{\prime }-n^{\prime }\right)i_{4}}\\ \; & =\Delta_{f}^{3}[|E\{a_{x}|a_{x}{|}^{2}{\}|}^{2}+|E\{a_{x}|a_{y}{|}^{2}{\}|}^{2}]\sum_{i_{1}=0}^{W-1}e^{-j\frac{2\pi }{W}\left(k-m+n\right)i_{1}}\sum_{i_{4}\neq i_{1}}e^{-j\frac{2\pi }{W}\left(-k^{\prime }+m^{\prime }-n^{\prime }\right)i_{4}}. \end{array} \end{array}$$

*Following a similar approach as in Example 3, we compute (32) by*

1. adding up the terms for all $i_{1}$ and $i_{4}$ values including all cases when $i_{1}$ and $i_{4}$ are equal to each other. These terms sum up to $W^{2}$ only when k−m+n=pW and $-k^{\prime }+m^{\prime }-n^{\prime }=pW$, with p∈Z; otherwise, they sum to 0;
2. subtracting the terms corresponding to the cases $i_{1}=i_{4}$. These terms sum to W only for $k-m+n-k^{\prime }+m^{\prime }-n^{\prime }=pW$, p∈Z; otherwise, they sum to zero.

*We, thus, obtain*

$$M_{2}^{\left(1\right)}=[|E\{a_{x}|a_{x}{|}^{2}{\}|}^{2}+|E\{a_{x}|a_{y}{|}^{2}{\}|}^{2}]\left[R_{s}^{2}\Delta_{f}\delta_{k-m+n-pW}\delta_{k^{\prime }-m^{\prime }+n^{\prime }-pW}-R_{s}\Delta_{f}^{2}\delta_{k-m+n-k^{\prime }+m^{\prime }-n^{\prime }-pW}\right],$$

*Following the same approach for $N_{2}^{\left(1\right)}$, we have*

$$\begin{array}{cc} N_{2}^{\left(1\right)} & ≜\sum_{i\in C_{1}^{\left(1\right)}}T_{i}\left(k,m,n,k^{\prime },m^{\prime },n^{\prime }\right)\\ \; & =E\{a_{x}|a_{x}{|}^{2}\}E\{a_{x}|a_{y}{|}^{2}\}\left[R_{s}^{2}\Delta_{f}\delta_{k-m+n}\delta_{k^{\prime }-m^{\prime }+n^{\prime }-pW}-R_{s}\Delta_{f}^{2}\delta_{k-m+n-k^{\prime }+m^{\prime }-n^{\prime }-pW}\right]. \end{array}$$

*All other contributions in $L_{2}$ can be computed using the approach used in this example.*

**Example** **5** (Contributions in $L_{3}$)**.** *$M_{3}^{\left(3\right)}$, i.e., the contribution for the values in the set $C_{3}^{\left(3\right)}=\left\{i\in {\left\{0,1,\ldots ,W-1\right\}}^{6}:i_{1}=i_{4},\;i_{2}=i_{3}=i_{5}=i_{6},\;i_{1}\neq i_{4},\;i_{2}\neq i_{3},\;i_{5}\neq i_{6}\right\}$ is given by*

(33) $$\begin{array}{cc} M_{3}^{\left(3\right)} & =\sum_{i\in C_{3}^{\left(3\right)}}S_{i}\left(k,m,n,k^{\prime },m^{\prime },n^{\prime }\right)\\ = & \Delta_{f}^{3}\left[E\{\right|a_{x,i_{1}}{|}^{2}\}E\{|a_{x,i_{2}}{|}^{4}\}+E\{|a_{y,i_{1}}{|}^{2}\}E\{|a_{x,i_{2}}{|}^{2}|a_{y,i_{2}}{|}^{2}\}]\sum_{i_{1}=1}^{W-1}e^{-j\frac{2\pi }{W}\left(k-k^{\prime }\right)i_{1}}\sum_{i_{2}\neq i_{1}}e^{-j\frac{2\pi }{W}\left(-m+n+m^{\prime }-n^{\prime }\right)i_{2}}\\ = & \Delta_{f}^{3}\left[E\{\right|a_{x}{|}^{2}\}E\{|a_{x}{|}^{4}\}+E\{|a_{y}{|}^{2}\}E\{|a_{x}{|}^{2}|a_{y}{|}^{2}\}]\sum_{i_{1}=1}^{W-1}e^{-j\frac{2\pi }{W}\left(k-k^{\prime }\right)i_{1}}\sum_{i_{2}\neq i_{1}}e^{-j\frac{2\pi }{W}\left(-m+n+m^{\prime }-n^{\prime }\right)i_{2}}. \end{array}$$

*As in the $L_{2}$ case described in Example 4, in $L_{3}$, each subset is characterized by 2 subgroups of indices. Hence, the approach followed to compute (33) is identical to (32) and gives*

$$\begin{array}{cc} M_{3}^{\left(3\right)} & =\left[E\{\right|a_{x}{|}^{4}\}E\{|a_{x}{|}^{2}\}+E\{|a_{x}{|}^{2}|a_{y}{|}^{2}\}E\{|a_{y}{|}^{2}\}][R_{s}^{2}\Delta_{f}\delta_{k-k^{\prime }-pW}\delta_{m-n-m^{\prime }+n^{\prime }-pW}\\ \; & -R_{s}\Delta_{f}^{2}\delta_{k-m+n-k^{\prime }+m^{\prime }-n^{\prime }-pW}]. \end{array}$$

*Similarly,*

$$\begin{array}{cc} N_{3}^{\left(3\right)} & =E\left\{a_{x}a_{y}^{*}\right\}E\{a_{x}^{*}a_{y}|a_{x}{|}^{2}\}\left[R_{s}^{2}\Delta_{f}\delta_{k-k^{\prime }-pW}\delta_{m-n-m^{\prime }+n^{\prime }-pW}-R_{s}\Delta_{f}^{2}\delta_{k-m+n-k^{\prime }+m^{\prime }-n^{\prime }-pW}\right]. \end{array}$$

*All other contributions in $L_{3}$ can be computed using the approach used in this example.*

As shown in the above examples, each contribution $M_{g}^{\left(h\right)}$ and $N_{g}^{\left(h\right)}$ is nonzero only for a specific set of $\left(k,m,n,k^{\prime },m^{\prime },n^{\prime }\right)$ values which is spanned by p∈Z. However, the terms $\left(k,m,n,k^{\prime },m^{\prime },n^{\prime }\right)$ arising for all p≠0 bring a total contribution to (25) that can be considered negligible. This is due to our assumption on P(f) being strictly band-limited (see Section 3.1) and to the magnitude of the functions product $\eta_{k,m,n}\eta_{k^{\prime },m^{\prime },n^{\prime }}^{*}$ (see definitions (16) and (22)). Thus, in the computations performed in the following subsections, we will restrict ourselves to the case p=0.

### 6.1. Contributions in $L_{1}$

In this section, the contributions $M_{1}^{\left(i\right)}$, $N_{1}^{\left(i\right)}$ for i=1,2,…,15 are computed following Example 3. These contributions are listed in Table 1.

### 6.2. Contributions in $L_{2}$

Following Example 4, the contributions $M_{2}^{\left(h\right)},N_{2}^{\left(h\right)},h=1,2,\ldots ,10$, are computed and listed in Table 2.

### 6.3. Contributions in $L_{3}$

Following Example 5, the contributions $M_{3}^{\left(h\right)},N_{3}^{\left(h\right)},h=1,2,\ldots ,15$, are computed and listed in Table 3.

### 6.4. Contributions in $L_{4}$

Since $L_{4}$ comprises a single subset characterised by the single subgroup of all 6 indices (see Section 5.2), only one pair of contributions $M_{4}^{\left(1\right)}$, $N_{4}^{\left(1\right)}$ exists, and it is given by

$$\begin{array}{cc} M_{4}^{\left(1\right)} & ≜\sum_{i\in C_{4}^{\left(1\right)}}S_{i}\left(k,m,n,k^{\prime },m^{\prime },n^{\prime }\right)\\ \; & =\sum_{i_{1}=0}^{W-1}\left[E\{\right|a_{x}{|}^{6}\}+E\{|a_{x}{|}^{2}|a_{y}{|}^{4}\}]e^{-j\frac{2\pi }{W}\left(k-m\right)i_{1}}\\ \; & =\left[E\{\right|a_{x}{|}^{6}\}+E\{|a_{x}{|}^{2}|a_{y}{|}^{4}\}]R_{s}\Delta_{f}^{2}\delta_{k-m+n-k^{\prime }+m^{\prime }-n^{\prime }},\\ N_{4}^{\left(1\right)} & ≜\sum_{i\in C_{4}^{\left(1\right)}}S_{i}\left(k,m,n,k^{\prime },m^{\prime },n^{\prime }\right)=E\{|a_{x}{|}^{4}|a_{y}{|}^{2}\}R_{s}\Delta_{f}^{2}\delta_{k-m+n-k^{\prime }+m^{\prime }-n^{\prime }}. \end{array}$$

## 7. Sum of All Contributions

In Section 5, we evaluated all contributions $M_{g}^{\left(h\right)}$ and $N_{g}^{\left(h\right)}$ to the PSD in (25). In particular, from (25)–(27), and (29), we have

(34) Sx(f,Ns,Ls)=892γ2Δf3∑i=−∞∞δ(f−iΔf)∑(k,m,n)∈Si(k′,m′,n′)∈SiP∑g=14∑h=1H(g)Mg(h)+2ReP∑g=14∑h=1H(g)Ng(h),

where H(g) is the number of subsets in the partitions of $L_{g}$, g=1,2,3,4, described in Section 5.2 (H(g)=15,10,15,1 for g=1,2,3,4, resp.) and

(35) $$P≜P_{k,m,n,k^{\prime },m^{\prime },n^{\prime }}\eta_{k,m,n}\eta_{k^{\prime },m^{\prime },n^{\prime }}^{*}.$$

In this section, we evaluate $\sum_{g=1}^{4}\sum_{h=1}^{H(g)}M_{g}^{\left(h\right)}$ and $\sum_{g=1}^{4}\sum_{h=1}^{H(g)}N_{g}^{\left(h\right)}$ as well as compact the resulting expression as much as possible.

Before we proceed with computing the abovementioned summation, we remove the Kronecker deltas in $M_{g}^{\left(h\right)}$ and $N_{g}^{\left(h\right)}$ corresponding to contributions in the following subspaces: (i) k=m; (ii) n=m; (iii) $k^{\prime }=m^{\prime }$; and (iv) $n^{\prime }=m^{\prime }$. These contributions correspond to so-called *bias terms*, i.e., they arise from a component of the field $E_{1}\left(f,z\right)$ which is fully correlated with the transmitted field $E_{0}\left(t,0\right)$. This component, after CDC and MF, only results in a deterministic and static complex scaling of the received constellation, which is typically compensated at the receiver even in the presence of other noise sources in the system. Thus, it does not contribute to the power of the additive zero-mean interference component that we observe at the output of the MF + sampling stage once the received constellation is synchronised (in phase and amplitude) with the transmitted one. A more detailed discussion on these bias terms can be found in [8] (Appendix A), [14] (Appendix C). Moreover, the component $\delta_{k-m+n}\delta_{k^{\prime }-m^{\prime }+n^{\prime }}$ in $M_{g}^{\left(h\right)}$ and $N_{g}^{\left(h\right)}$ is also removed as it only gives nonzero contribution to the PSD for frequency $f=i\Delta_{f}=0$; hence, its effect on the total NLI variance vanishes as we let $\Delta_{f}\to 0$ (see Section 8). A total of 23 terms from the last columns of Table 1, Table 2 and Table 3 are thus removed. The remaining contributions are given in Table A4 in Appendix D.

We now compact the contributions in Table A4 by grouping the Kronecker delta products based on each correlation term they multiply. We use three pairs of curly brackets {·} to denote the terms multiplying $R_{s}^{3}$, $R_{s}^{2}$, and $R_{s}$. The list of all Kronecker delta products multiplying each correlation term is shown in Table 4. The correlation terms are divided into intra-polarisation (expectations containing only $a_{x}$) and cross-polarisation terms (expectations containing both $a_{x}$ and $a_{y}$). Moreover, the correlations are categorised based on the specific contribution (either M or N) in (34) to which they belong.

As it can be observed in Table 4, each correlation term is associated with different delta functions. To compact these terms, we exploit a property introduced in the following proposition.

**Proposition** **2.** *Let $D_{1}\left(k,m,n,k^{\prime },m^{\prime },n^{\prime }\right)$ and $D_{2}\left(k,m,n,k^{\prime },m^{\prime },n^{\prime }\right)$ be two Kronecker delta products of the kind shown in Table 4. If*

(36) $$D_{1}\left(k,m,n,k^{\prime },m^{\prime },n^{\prime }\right)=D_{2}\left(n,m,k,n^{\prime },m^{\prime },k^{\prime }\right),$$

*then*

(37) $$\begin{array}{c} \begin{array}{cc} \; & \sum_{\begin{array}{c} \left(k,m,n\right)\in S_{i}\\ \left(k^{\prime },m^{\prime },n^{\prime }\right)\in S_{i} \end{array}}P_{k,m,n,k^{\prime },m^{\prime },n^{\prime }}\eta_{k,m,n}\eta_{k^{\prime },m^{\prime },n^{\prime }}^{*}D_{1}\left(k,m,n,k^{\prime },m^{\prime },n^{\prime }\right)\\ \; & =\sum_{\begin{array}{c} \left(k,m,n\right)\in S_{i}\\ \left(k^{\prime },m^{\prime },n^{\prime }\right)\in S_{i} \end{array}}P_{k,m,n,k^{\prime },m^{\prime },n^{\prime }}\eta_{k,m,n}\eta_{k^{\prime },m^{\prime },n^{\prime }}^{*}D_{2}\left(k,m,n,k^{\prime },m^{\prime },n^{\prime }\right). \end{array} \end{array}$$

*This property also holds when applying the transformations k=n, n=k, $k^{\prime }=n^{\prime }$, and $n^{\prime }=k^{\prime }$, individually.*

**Proof.**  See Appendix E. □

The property in (37) allows us to group many of the Kronecker function products in Table 4 under a single term. Namely, the Kronecker delta products in Table 4 can be grouped in subsets that are closed to property (36), since they all result in the same value of summations in (37). In particular, 14 distinct subsets can be identified for the list of Kronecker delta products in Table 4. We label these subsets, which are shown in Table 5, as $D_{l}$ for l=1,2,…,14.

Summing all the contributions in Table 4, using Proposition 2 for the elements in the subsets listed in Table 4, and finally ordering by Kronecker delta product, we obtain from (34)

(38) Sx(f,Ns,Ls)=892γ2Δf∑i=−∞∞δ(f−iΔf)·∑(k,m,n)∈Si(k′,m′,n′)∈Si[Rs3Δf2[(a1P+2Re{a1′P})δk−k′δm−m′δn−n′+(a2P+2Re{a2′P})δk−k′δm+n′δn+m′+(a3P+2Re{a3′P})δk+nδm−m′δk′+n′]+Rs2Δf3[(b1P+2Re{b1′P})δk−m−k′δn+m′−n′+(b2P+2Re{b2′P})δk−m+m′δn−k′−n′+(b2∗P+2Re{b3′P})δk+n−k′δm−m′+n′+(b4P+2Re{b4′P})δk+n+m′δm+k′+n′+(c1P+2Re{c1′P})δk+nδm+k′−m′+n′+(c2P+2Re{c2′P})δk−k′δm−n−m′+n′+(c3P+2Re{c3′P})δk+m′δm−n+k′+n′+(c4P+2Re{c4′P})δm−m′δk+n−k′−n′+(c3∗P+2Re{c5′P})δm+k′δk+n+m′−n′+(c1∗P+2Re{c6′P})δk′+n′δk−m+n+m′]+RsΔf4(d1P+2Re{d1′P})δk−m+n−k′+m′−n′],

where the coefficients multiplying P are listed in Table A5 in Appendix F and where coset leaders in Table 5 have been used.

**Table 4:** List of Kronecker delta contributions ordered by the corresponding high-order moment or correlation of the transmitted modulation format.

Correlation Terms	Kronecker Delta Products
**Intra-Polarisation Terms**	
**In $M_{g}^{\left(h\right)}$**	
$E^{3}\left\{\right|a_{x}{|}^{2}\}$	{$\delta_{k-k^{\prime }}\delta_{m-m^{\prime }}\delta_{n-n^{\prime }},\delta_{k-n^{\prime }}\delta_{m-m^{\prime }}\delta_{n-k^{\prime }}$},{$-2\delta_{n-k^{\prime }}\delta_{k-m+m^{\prime }-n^{\prime }},-2\delta_{n-n^{\prime }}\delta_{k-m-k^{\prime }+m^{\prime }},$
	$-2\delta_{k-k^{\prime }}\delta_{m-n-m^{\prime }+n^{\prime }},-2\delta_{m-m^{\prime }}\delta_{k+n-k^{\prime }-n^{\prime }},-2\delta_{k-n^{\prime }}\delta_{m-n+k^{\prime }-m^{\prime }}$},{$12\delta_{k-m+n-k^{\prime }+m^{\prime }-n^{\prime }}$}
$E\{|a_{x}{|}^{2}\}|E\left\{a_{x}^{2}\right\}{|}^{2}$	{$\delta_{k+n}\delta_{m-m^{\prime }}\delta_{k^{\prime }+n^{\prime }},\delta_{k-k^{\prime }}\delta_{m+n^{\prime }}\delta_{n+m^{\prime }},\delta_{k+m^{\prime }}\delta_{m+k^{\prime }}\delta_{n-n^{\prime }},\delta_{k+m^{\prime }}\delta_{m+n^{\prime }}\delta_{n-k^{\prime }},$
	$\delta_{k-n^{\prime }}\delta_{m+k^{\prime }}\delta_{n+m^{\prime }}$},{$-\delta_{n-k^{\prime }}\delta_{k-m+m^{\prime }-n^{\prime }},-3\delta_{m+k^{\prime }}\delta_{k+n+m^{\prime }-n^{\prime }},-3\delta_{k+n}\delta_{m+k^{\prime }-m^{\prime }+n^{\prime }}$
	$-3\delta_{k^{\prime }+n^{\prime }}\delta_{k+n-m+m^{\prime }},-\delta_{m-m^{\prime }}\delta_{k+n-k^{\prime }-n^{\prime }},-3\delta_{m+n^{\prime }}\delta_{k+n-k^{\prime }+m^{\prime }},-3\delta_{n+m^{\prime }}\delta_{k-m-k^{\prime }-n^{\prime }},$
	$-\delta_{k-k^{\prime }}\delta_{m-n-m^{\prime }+n^{\prime }},-3\delta_{k+m^{\prime }}\delta_{m-n+k^{\prime }+n^{\prime }},-\delta_{n-n^{\prime }}\delta_{k-m-k^{\prime }+m^{\prime }},-\delta_{k-n^{\prime }}\delta_{m-n+k^{\prime }-m^{\prime }},$}
	{$18\delta_{k-m+n-k^{\prime }+m^{\prime }-n^{\prime }}$}
$|E\{a_{x}|a_{x}{|}^{2}{\}|}^{2}$	{}, {$\delta_{k-m-k^{\prime }}\delta_{n+m^{\prime }-n^{\prime }},\delta_{k-m+m^{\prime }}\delta_{n-k^{\prime }-n^{\prime }},\delta_{k-m-n^{\prime }}\delta_{n-k^{\prime }+m^{\prime }},\delta_{k+n-k^{\prime }}\delta_{m-m^{\prime }+n^{\prime }},$
	$\delta_{k+n-n^{\prime }}\delta_{m+k^{\prime }-m^{\prime }},\delta_{k-k^{\prime }+m^{\prime }}\delta_{m-n+n^{\prime }},\delta_{k-k^{\prime }-n^{\prime }}\delta_{m-n+m^{\prime }},\delta_{k+m^{\prime }-n^{\prime }}\delta_{m-n+k^{\prime }}$},
	{$-9\delta_{k-m+n-k^{\prime }+m^{\prime }-n^{\prime }}$}
$|E\left\{a_{x}^{3}\right\}{|}^{2}$	{}, $\left\{\delta_{k+n+m^{\prime }}\delta_{m+k^{\prime }+n^{\prime }}\right\},\left\{-\delta_{k-m+n-k^{\prime }+m^{\prime }-n^{\prime }}\right\}$
$E\{|a_{x}{|}^{4}\}E\{|a_{x}{|}^{2}\}$	{}, $\{\delta_{k-k^{\prime }}\delta_{m-n-m^{\prime }+n^{\prime }},\delta_{k-n^{\prime }}\delta_{m-n+k^{\prime }-m^{\prime }},\delta_{m-m^{\prime }}\delta_{k+n-k^{\prime }-n^{\prime }},\delta_{n-k^{\prime }}\delta_{k-m+m^{\prime }-n^{\prime }},$
	$\delta_{n-n^{\prime }}\delta_{k-m-k^{\prime }+m^{\prime }}\},\left\{-9\delta_{k-m+n-k^{\prime }+m^{\prime }-n^{\prime }}\right\}$
$E^{*}\{a_{x}^{2}|a_{x}{|}^{2}\}E\left\{a_{x}^{2}\right\}$	{}, $\left\{\delta_{k+n}\delta_{m+k^{\prime }-m^{\prime }+n^{\prime }},\delta_{k+m^{\prime }}\delta_{m-n+k^{\prime }+n^{\prime }},\delta_{n+m^{\prime }}\delta_{k-m-k^{\prime }-n^{\prime }}\right\}$,
	$\left\{-3\delta_{k-m+n-k^{\prime }+m^{\prime }-n^{\prime }}\right\}$
$E\{a_{x}^{2}|a_{x}{|}^{2}\}E^{*}\left\{a_{x}^{2}\right\}$	{}, $\left\{\delta_{m+k^{\prime }}\delta_{k-n+m^{\prime }-n^{\prime }},\delta_{m+n^{\prime }}\delta_{k+n-k^{\prime }+m^{\prime }},\delta_{k^{\prime }+n^{\prime }}\delta_{k-m+n+m^{\prime }}\right\},\left\{-3\delta_{k-m+n-k^{\prime }+m^{\prime }-n^{\prime }}\right\}$
$E\{|a_{x}{|}^{6}\}$	{}, {}, $\left\{\delta_{k-m+n-k^{\prime }+m^{\prime }-n^{\prime }}\right\}$
**Cross-polarisation terms**	
**In $M_{g}^{\left(h\right)}$**	
$E\{|a_{x}{|}^{2}\}E^{2}\left\{\right|a_{y}{|}^{2}\}$	$\left\{\delta_{k-k^{\prime }}\delta_{m-m^{\prime }}\delta_{n-n^{\prime }}\right\},\left\{-2\delta_{n-n^{\prime }}\delta_{k-m-k^{\prime }+m^{\prime }},-\delta_{m-m^{\prime }}\delta_{k+n-k^{\prime }-n^{\prime }},-\delta_{k-k^{\prime }}\delta_{m-n-m^{\prime }+n^{\prime }}\right\},$
	$\left\{4\delta_{k-m+n-k^{\prime }+m^{\prime }-n^{\prime }}\right\}$
$E\{|a_{x}{|}^{2}\}|E\left\{a_{y}^{2}\right\}{|}^{2}$	$\left\{\delta_{k+m^{\prime }}\delta_{m+k^{\prime }}\delta_{n-n^{\prime }}\right\},\left\{-\delta_{n-n^{\prime }}\delta_{k-m-k^{\prime }+m^{\prime }},-\delta_{m+k^{\prime }}\delta_{k+n+m^{\prime }-n^{\prime }},-\delta_{k+m^{\prime }}\delta_{m-n+k^{\prime }+n^{\prime }}\right\},$
	$\left\{2\delta_{k-m+n-k^{\prime }+m^{\prime }-n^{\prime }}\right\}$
$E\{|a_{x}{|}^{2}\}E\{|a_{y}{|}^{4}\}$	{}, {$\delta_{n-n^{\prime }}\delta_{k-m-k^{\prime }+m^{\prime }}\},\left\{-\delta_{k-m+n-k^{\prime }+m^{\prime }-n^{\prime }}\right\}$
$E\{|a_{x}{|}^{2}a_{y}^{*}\}E\{a_{y}|a_{y}{|}^{2}\}$	{}, $\left\{\delta_{k-m+m^{\prime }}\delta_{n-k^{\prime }-n^{\prime }},\delta_{k-k^{\prime }+m^{\prime }}\delta_{m-n+n^{\prime }}\right\},\left\{-2\delta_{k-m+n-k^{\prime }+m^{\prime }-n^{\prime }}\right\}$
$E\{|a_{x}{|}^{2}|a_{y}{|}^{2}\}E\{|a_{y}{|}^{2}\}$	{}, $\left\{\delta_{k-k^{\prime }}\delta_{m-n-m^{\prime }+n^{\prime }},\delta_{m-m^{\prime }}\delta_{k+n-k^{\prime }-n^{\prime }}\right\},\left\{-4\delta_{k-m+n-k^{\prime }+m^{\prime }-n^{\prime }}\right\}$
$E\left\{a_{x}a_{y}\right\}E^{*}\{a_{x}a_{y}|a_{x}{|}^{2}\}$	{}, $\{\delta_{k+m^{\prime }}\delta_{m-n+k^{\prime }+n^{\prime }},\delta_{m+k^{\prime }}\delta_{k-n+m^{\prime }-n^{\prime }},\delta_{n+m^{\prime }}\delta_{k-m-k^{\prime }-n^{\prime }},\delta_{k^{\prime }+n^{\prime }}\delta_{k-m+n+m^{\prime }}\},$
	$\left\{-4\delta_{k-m+n-k^{\prime }+m^{\prime }-n^{\prime }}\right\}$
$E^{*}\left\{\right|a_{x}{|}^{2}a_{y}^{2}\}E\left\{a_{y}^{2}\right\}$	{}, $\left\{\delta_{k+m^{\prime }}\delta_{m-n+k^{\prime }+n^{\prime }}\right\},\left\{-\delta_{k-m+n-k^{\prime }+m^{\prime }-n^{\prime }}\right\}$
$E\{|a_{x}{|}^{2}a_{y}^{2}\}E^{*}\left\{a_{y}^{2}\right\}$	{}, $\left\{\delta_{m+k^{\prime }}\delta_{k-n+m^{\prime }-n^{\prime }}\right\},\left\{-\delta_{k-m+n-k^{\prime }+m^{\prime }-n^{\prime }}\right\}$
$E\{|a_{x}{|}^{2}|a_{y}{|}^{4}\}$	{}, {}, $\left\{\delta_{k-m+n-k^{\prime }+m^{\prime }-n^{\prime }}\right\}$
$|E\left\{a_{x}a_{y}^{*}\right\}{|}^{2}E\{|a_{y}{|}^{2}\}$	$\left\{\delta_{k-n^{\prime }}\delta_{m-m^{\prime }}\delta_{n-k^{\prime }}\right\},\{-2\delta_{n-k^{\prime }}\delta_{k-m+m^{\prime }-n^{\prime }},-2\delta_{k-n^{\prime }}\delta_{m-n+k^{\prime }-m^{\prime }},-\delta_{k-k^{\prime }}\delta_{m-n-m^{\prime }+n^{\prime }},$
	$-\delta_{m-m^{\prime }}\delta_{k+n-k^{\prime }-n^{\prime }}\},\left\{8\delta_{k-m+n-k^{\prime }+m^{\prime }-n^{\prime }}\right\}$
$E\left\{a_{x}a_{y}\right\}E\left\{a_{x}^{*}a_{y}\right\}E^{*}\left\{a_{y}^{2}\right\}$	$\left\{\delta_{k-n^{\prime }}\delta_{m+k^{\prime }}\delta_{n+m^{\prime }}\right\},\{-2\delta_{m+k^{\prime }}\delta_{k+n+m^{\prime }-n^{\prime }},-\delta_{k+n}\delta_{m+k^{\prime }-m^{\prime }+n^{\prime }},-\delta_{n+m^{\prime }}\delta_{k-m-k^{\prime }-n^{\prime }},$
	$-\delta_{k-n^{\prime }}\delta_{m-n+k^{\prime }-m^{\prime }}\},\left\{4\delta_{k-m+n-k^{\prime }+m^{\prime }-n^{\prime }}\right\}$
$E^{*}\left\{a_{x}a_{y}\right\}E\left\{a_{x}a_{y}^{*}\right\}E\left\{a_{y}^{2}\right\}$	$\left\{\delta_{k+m^{\prime }}\delta_{m+n^{\prime }}\delta_{n-k^{\prime }}\right\},\{-2\delta_{k+m^{\prime }}\delta_{m-n+k^{\prime }+n^{\prime }},-\delta_{k^{\prime }+n^{\prime }}\delta_{k-m+n+m^{\prime }},-\delta_{n-k^{\prime }}\delta_{k-m+m^{\prime }-n^{\prime }},$
	$-\delta_{m+n^{\prime }}\delta_{k+n-k^{\prime }+m^{\prime }}\},\left\{4\delta_{k-m+n-k^{\prime }+m^{\prime }-n^{\prime }}\right\}$
$|E\left\{a_{x}a_{y}\right\}{|}^{2}E\{|a_{y}{|}^{2}\}$	{$\delta_{k+n}\delta_{m-m^{\prime }}\delta_{k^{\prime }+n^{\prime }},\delta_{k-k^{\prime }}\delta_{m+n^{\prime }}\delta_{n+m^{\prime }}\},\{-2\delta_{k^{\prime }+n^{\prime }}\delta_{k-m+n+m^{\prime }},-2\delta_{k+n}\delta_{m-m^{\prime }+k^{\prime }+n^{\prime }},$
	$-2\delta_{n+m^{\prime }}\delta_{k-m-k^{\prime }-n^{\prime }},-2\delta_{m+n^{\prime }}\delta_{k+n-k^{\prime }+m^{\prime }},-\delta_{m-m^{\prime }}\delta_{k+n-k^{\prime }-n^{\prime }},-\delta_{k-k^{\prime }}\delta_{m-n-m^{\prime }+n^{\prime }}\}$,
	$\left\{8\delta_{k-m+n-k^{\prime }+m^{\prime }-n^{\prime }}\right\}$
$|E\{a_{x}|a_{y}{|}^{2}{\}|}^{2}$	{}, $\left\{\delta_{k-m-n^{\prime }}\delta_{n-k^{\prime }+m^{\prime }},\delta_{k+n-k^{\prime }}\delta_{m-m^{\prime }+n^{\prime }},\delta_{k-k^{\prime }-n^{\prime }}\delta_{m-n+m^{\prime }}\right\},\left\{-4\delta_{k-m+n-k^{\prime }+m^{\prime }-n^{\prime }}\right\}$
$|E\left\{a_{x}a_{y}^{2}\right\}{|}^{2}$	{}, $\left\{\delta_{k+n+m^{\prime }}\delta_{m+k^{\prime }+n^{\prime }}\right\},\left\{-\delta_{k-m+n-k^{\prime }+m^{\prime }-n^{\prime }}\right\}$
$|E\left\{a_{x}^{*}a_{y}^{2}\right\}{|}^{2}$	{}, $\left\{\delta_{k+m^{\prime }-n^{\prime }}\delta_{m-n+k^{\prime }}\right\},\left\{-\delta_{k-m+n-k^{\prime }+m^{\prime }-n^{\prime }}\right\}$
**In $N_{g}^{\left(h\right)}$**	
$E\{|a_{x}{|}^{2}\}|E\left\{a_{x}a_{y}^{*}\right\}{|}^{2}$	{$\delta_{k-k^{\prime }}\delta_{m-m^{\prime }}\delta_{n-n^{\prime }},\delta_{k-n^{\prime }}\delta_{m-m^{\prime }}\delta_{n-k^{\prime }}\},\{-2\delta_{n-k^{\prime }}\delta_{k-m+m^{\prime }-n^{\prime }},-2\delta_{k-k^{\prime }}\delta_{m-n-m^{\prime }+n^{\prime }},$
	$-2\delta_{m-m^{\prime }}\delta_{k+n-k^{\prime }-n^{\prime }},-\delta_{n-n^{\prime }}\delta_{k-m-k^{\prime }+m^{\prime }},-\delta_{k-n^{\prime }}\delta_{m-n+k^{\prime }-m^{\prime }}\},\left\{8\delta_{k-m+n-k^{\prime }+m^{\prime }-n^{\prime }}\right\}$
$E\{|a_{x}{|}^{2}\}|E\left\{a_{x}a_{y}\right\}{|}^{2}$	$\left\{\delta_{k+m^{\prime }}\delta_{m+k^{\prime }}\delta_{n-n^{\prime }},\delta_{k-n^{\prime }}\delta_{m+k^{\prime }}\delta_{n+m^{\prime }}\right\},\{-2\delta_{k^{\prime }+n^{\prime }}\delta_{k-m+n+m^{\prime }},-2\delta_{n+m^{\prime }}\delta_{k-m-k^{\prime }-n^{\prime }},$
	$-2\delta_{k+m^{\prime }}\delta_{m-n+k^{\prime }+n^{\prime }},-2\delta_{m+k^{\prime }}\delta_{k+n+m^{\prime }-n^{\prime }},-\delta_{n-n^{\prime }}\delta_{k-m-k^{\prime }+m^{\prime }},-\delta_{k-n^{\prime }}\delta_{m-n+k^{\prime }-m^{\prime }}\}$,
	$\{8\delta_{k-m+n-k^{\prime }+m^{\prime }-n^{\prime }}\}.$
$E^{2}\left\{\right|a_{x}{|}^{2}\}E\{|a_{y}{|}^{2}\}$	{}, $\left\{-\delta_{n-n^{\prime }}\delta_{k-m-k^{\prime }+m^{\prime }},-\delta_{k-n^{\prime }}\delta_{m-n+k^{\prime }-m^{\prime }}\right\},\left\{4\delta_{k-m+n-k^{\prime }+m^{\prime }-n^{\prime }}\right\}$
$E\{|a_{x}{|}^{2}\}E\{|a_{x}{|}^{2}|a_{y}{|}^{2}\}$	{}, $\left\{\delta_{k-n^{\prime }}\delta_{m-n+k^{\prime }-m^{\prime }},\delta_{n-n^{\prime }}\delta_{k-m-k^{\prime }+m^{\prime }}\right\},\{-4\delta_{k-m+n-k^{\prime }+m^{\prime }-n^{\prime }}$}
$E\{|a_{x}{|}^{4}\}E\{|a_{y}{|}^{2}\}$	{}, {}, $\left\{-\delta_{k-m+n-k^{\prime }+m^{\prime }-n^{\prime }}\right\}$
$E\{a_{x}|a_{x}{|}^{2}\}E\{a_{x}|a_{y}{|}^{2}\}$	{}, {}, $\left\{-\delta_{k-m+n-k^{\prime }+m^{\prime }-n^{\prime }}\right\}$
$E\{a_{y}^{*}|a_{y}{|}^{2}\}E\{|a_{x}{|}^{2}a_{y}\}$	{}, $\left\{\delta_{k-m-k^{\prime }}\delta_{n+m^{\prime }-n^{\prime }},\delta_{k+n-n^{\prime }}\delta_{m+k^{\prime }-m^{\prime }}\right\},\left\{-2\delta_{k-m+n-k^{\prime }+m^{\prime }-n^{\prime }}\right\}$
$E\{a_{x}^{*}|a_{x}{|}^{2}\}E\{a_{x}|a_{y}{|}^{2}\}$	{}, $\left\{\delta_{k-m-n^{\prime }}\delta_{n-k^{\prime }+m^{\prime }},\delta_{k+n-n^{\prime }}\delta_{m+k^{\prime }-m^{\prime }}\right\},\left\{-2\delta_{k-m+n-k^{\prime }+m^{\prime }-n^{\prime }}\right\}$
$\left|E\{\right|a_{x}{|}^{2}a_{y}{\}|}^{2}$	{}, $\{\delta_{k-m-k^{\prime }}\delta_{n+m^{\prime }-n^{\prime }},\delta_{k-m+m^{\prime }}\delta_{n-k^{\prime }-n^{\prime }},\delta_{k-k^{\prime }-n^{\prime }}\delta_{m-n+m^{\prime }},\delta_{k+m^{\prime }-n^{\prime }}\delta_{m-n+k^{\prime }}\},$
	$\left\{-4\delta_{k-m+n-k^{\prime }+m^{\prime }-n^{\prime }}\right\}$
$E\left\{a_{x}a_{y}^{*}\right\}E\{a_{x}^{*}a_{y}|a_{x}{|}^{2}\}$	{}, $\left\{\delta_{k-k^{\prime }}\delta_{m-n-m^{\prime }+n^{\prime }},\delta_{m-m^{\prime }}\delta_{k+n-k^{\prime }-n^{\prime }},\delta_{n-k^{\prime }}\delta_{k-m+m^{\prime }-n^{\prime }}\right\},\left\{-4\delta_{k-m+n-k^{\prime }+m^{\prime }-n^{\prime }}\right\}$
$E\{|a_{x}{|}^{4}|a_{y}{|}^{2}\}$	{}, {}, $\left\{\delta_{k-m+n-k^{\prime }+m^{\prime }-n^{\prime }}\right\}$
$E\left\{a_{x}^{2}\right\}E^{*}\left\{a_{x}a_{y}\right\}E\left\{a_{x}^{*}a_{y}\right\}$	$\left\{\delta_{k+n}\delta_{m-m^{\prime }}\delta_{k^{\prime }+n^{\prime }}\right\},\{-2\delta_{k+n}\delta_{m+k^{\prime }-m^{\prime }+n^{\prime }},-\delta_{m+k^{\prime }}\delta_{k+n+m^{\prime }-n^{\prime }},-\delta_{k^{\prime }+n^{\prime }}\delta_{k+n-m+m^{\prime }},$
	$-\delta_{m-m^{\prime }}\delta_{k+n-k^{\prime }-n^{\prime }}\},\left\{4\delta_{k-m+n-k^{\prime }+m^{\prime }-n^{\prime }}\right\}$
$E^{*}\left\{a_{x}^{2}\right\}E\left\{a_{x}a_{y}\right\}E\left\{a_{x}a_{y}^{*}\right\}$	$\left\{\delta_{k-k^{\prime }}\delta_{m+n^{\prime }}\delta_{n+m^{\prime }},\delta_{k+m^{\prime }}\delta_{m+n^{\prime }}\delta_{n-k^{\prime }}\right\},\{-2\delta_{m+n^{\prime }}\delta_{k+n-k^{\prime }+m^{\prime }},-\delta_{n+m^{\prime }}\delta_{k-m-k^{\prime }-n^{\prime }},$
	$-\delta_{k-k^{\prime }}\delta_{m-n-m^{\prime }+n^{\prime }},-\delta_{n-k^{\prime }}\delta_{k-m+m^{\prime }-n^{\prime }},-\delta_{k+m^{\prime }}\delta_{m-n+k^{\prime }+n^{\prime }}\},\left\{4\delta_{k-m+n-k^{\prime }+m^{\prime }-n^{\prime }}\right\}$
$|E\left\{a_{x}^{2}\right\}{|}^{2}E\{|a_{y}{|}^{2}\}$	{}, $\left\{-\delta_{m+n^{\prime }}\delta_{k+n-k^{\prime }+m^{\prime }},-\delta_{k+n}\delta_{m+k^{\prime }-m^{\prime }+n^{\prime }}\right\},\left\{2\delta_{k-m+n-k^{\prime }+m^{\prime }-n^{\prime }}\right\}$
$|E\left\{a_{x}^{2}a_{y}^{*}\right\}{|}^{2}$	{}, $\left\{\delta_{k+n-k^{\prime }}\delta_{m-m^{\prime }+n^{\prime }}\right\},\left\{-\delta_{k-m+n-k^{\prime }+m^{\prime }-n^{\prime }}\right\}$
$|E\left\{a_{x}^{2}a_{y}\right\}{|}^{2}$	{}, $\left\{\delta_{k+n+m^{\prime }}\delta_{m+k^{\prime }+n^{\prime }}\right\},\left\{-\delta_{k-m+n-k^{\prime }+m^{\prime }-n^{\prime }}\right\}$
$E\left\{a_{x}^{2}\right\}E^{*}\{a_{x}^{2}|a_{y}{|}^{2}\}$	{}, $\left\{\delta_{k+n}\delta_{m+k^{\prime }-m^{\prime }+n^{\prime }},\delta_{m+n^{\prime }}\delta_{k+n-k^{\prime }+m^{\prime }}\right\},\left\{-2\delta_{k-m+n-k^{\prime }+m^{\prime }-n^{\prime }}\right\}$
$E\left\{a_{x}a_{y}\right\}E^{*}\{a_{x}a_{y}|a_{y}{|}^{2}\}$	{}, $\left\{\delta_{k+n}\delta_{m+k^{\prime }-m^{\prime }+n^{\prime }},\delta_{n+m^{\prime }}\delta_{k-m-k^{\prime }-n^{\prime }}\right\},\left\{-2\delta_{k-m+n-k^{\prime }+m^{\prime }-n^{\prime }}\right\}$
$E^{*}\left\{a_{x}a_{y}\right\}E\{a_{x}a_{y}|a_{y}{|}^{2}\}$	{}, $\left\{\delta_{m+n^{\prime }}\delta_{k+n-k^{\prime }+m^{\prime }},\delta_{k^{\prime }+n^{\prime }}\delta_{k-m+n+m^{\prime }}\right\},\left\{-2\delta_{k-m+n-k^{\prime }+m^{\prime }-n^{\prime }}\right\}$
$E\left\{a_{x}^{*}a_{y}\right\}E\{a_{x}a_{y}^{*}|a_{y}{|}^{2}\}$	{}, $\left\{\delta_{k-n^{\prime }}\delta_{m-n+k^{\prime }-m^{\prime }}\right\},\left\{-\delta_{k-m+n-k^{\prime }+m^{\prime }-n^{\prime }}\right\}$
$E\left\{a_{x}a_{y}^{*}\right\}E\{a_{x}^{*}a_{y}|a_{y}{|}^{2}\}$	{}, $\left\{\delta_{n-k^{\prime }}\delta_{k-m+m^{\prime }-n^{\prime }}\right\},\left\{-\delta_{k-m+n-k^{\prime }+m^{\prime }-n^{\prime }}\right\}$

**Table 5:** Subsets of Kronecker delta products which are closed to property (36). The terms in boldface are the ones used to group all other elements within each set.

Set Name	Set Elements
$D_{1}$	$\delta_{k-k^{\prime }}\delta_{m-m^{\prime }}\delta_{n-n^{\prime }},\delta_{k-n^{\prime }}\delta_{m-m^{\prime }}\delta_{n-k^{\prime }}$
$D_{2}$	$\delta_{k-k^{\prime }}\delta_{m+n^{\prime }}\delta_{n+m^{\prime }},\delta_{k+m^{\prime }}\delta_{m+k^{\prime }}\delta_{n-n^{\prime }},\delta_{k+m^{\prime }}\delta_{m+n^{\prime }}\delta_{n-k^{\prime }},\delta_{k-n^{\prime }}\delta_{m+k^{\prime }}\delta_{n+m^{\prime }}$
$D_{3}$	$\delta_{k+n}\delta_{m-m^{\prime }}\delta_{k^{\prime }+n^{\prime }}$
$D_{4}$	$\delta_{k-m-k^{\prime }}\delta_{n+m^{\prime }-n^{\prime }},\delta_{k-m-n^{\prime }}\delta_{n-k^{\prime }+m^{\prime }},\delta_{k+m^{\prime }-n^{\prime }}\delta_{m-n+k^{\prime }},\delta_{k-k^{\prime }+m^{\prime }}\delta_{m-n+n^{\prime }}$
$D_{5}$	$\delta_{k-m+m^{\prime }}\delta_{n-k^{\prime }-n^{\prime }},\delta_{k-k^{\prime }-n^{\prime }}\delta_{m-n-m^{\prime }}$
$D_{6}$	$\delta_{k+n-k^{\prime }}\delta_{m-m^{\prime }+n^{\prime }},\delta_{k+n-n^{\prime }}\delta_{m+k^{\prime }-m^{\prime }}$
$D_{7}$	$\delta_{k+n+m^{\prime }}\delta_{m+k^{\prime }+n^{\prime }}$
$D_{8}$	$\delta_{k+n}\delta_{m+k^{\prime }-m^{\prime }+n^{\prime }}$
$D_{9}$	$\delta_{k-k^{\prime }}\delta_{m-n-m^{\prime }+n^{\prime }},\delta_{k-n^{\prime }}\delta_{m-n+k^{\prime }-m^{\prime }},\delta_{n-k^{\prime }}\delta_{k-m+m^{\prime }-n^{\prime }},\delta_{n-n^{\prime }}\delta_{k-m-k^{\prime }+m^{\prime }}$
$D_{10}$	$\delta_{k+m^{\prime }}\delta_{m-n+k^{\prime }+n^{\prime }},\delta_{n+m^{\prime }}\delta_{k-m-k^{\prime }-n^{\prime }}$
$D_{11}$	$\delta_{m-m^{\prime }}\delta_{k+n-k^{\prime }-n^{\prime }}$
$D_{12}$	$\delta_{m+k^{\prime }}\delta_{k+n+m^{\prime }-n^{\prime }},\delta_{m+n^{\prime }}\delta_{k+n-k^{\prime }+m^{\prime }}$
$D_{13}$	$\delta_{k^{\prime }+n^{\prime }}\delta_{k-m+n+m^{\prime }}$
$D_{14}$	$\delta_{k-m+n-k^{\prime }+m^{\prime }-n^{\prime }}$

Equation (38) can be further manipulated using the following proposition.

**Proposition** **3.** *Let $D_{1}\left(k,m,n,k^{\prime },m^{\prime },n^{\prime }\right)$ and $D_{2}\left(k,m,n,k^{\prime },m^{\prime },n^{\prime }\right)$ be two Kronecker delta products of the kind shown in the second column of Table 4. If $D_{1}\left(k,m,n,k^{\prime },m^{\prime },n^{\prime }\right)=D_{2}\left(k^{\prime },m^{\prime },n^{\prime },k,m,n\right)$, then*

(39) $$\begin{array}{cc} \; & \sum_{\begin{array}{c} \left(k,m,n\right)\in S_{i}\\ \left(k^{\prime },m^{\prime },n^{\prime }\right)\in S_{i} \end{array}}P_{k,m,n,k^{\prime },m^{\prime },n^{\prime }}\eta_{k,m,n}\eta_{k^{\prime },m^{\prime },n^{\prime }}^{*}D_{1}\left(k,m,n,k^{\prime },m^{\prime },n^{\prime }\right)\\ \; & ={\left(\sum_{\begin{array}{c} \left(k,m,n\right)\in S_{i}\\ \left(k^{\prime },m^{\prime },n^{\prime }\right)\in S_{i} \end{array}}P_{k,m,n,k^{\prime },m^{\prime },n^{\prime }}\eta_{k,m,n}\eta_{k^{\prime },m^{\prime },n^{\prime }}^{*}D_{2}\left(k,m,n,k^{\prime },m^{\prime },n^{\prime }\right)\right)}^{*}. \end{array}$$

**Proof.**  See Appendix G. □

**Corollary** **1.** *Let $D(k,m,n,k^{\prime },m^{\prime },n^{\prime })$ be a Kronecker delta product for which the following property holds*

$$D\left(k,m,n,k^{\prime },m^{\prime },n^{\prime }\right)=D\left(k^{\prime },m^{\prime },n^{\prime },k,m,n\right),$$

*then $\sum_{\begin{array}{c} \left(k,m,n\right)\in S_{i}\\ \left(k^{\prime },m^{\prime },n^{\prime }\right)\in S_{i} \end{array}}P_{k,m,n,k^{\prime },m^{\prime },n^{\prime }}\eta_{k,m,n}\eta_{k^{\prime },m^{\prime },n^{\prime }}^{*}D\left(k,m,n,k^{\prime },m^{\prime },n^{\prime }\right)\in R.$*

**Proof.**  This corollary directly follows from Proposition 3 when $D_{1}=D_{2}=D$. □

Based on Corollary 1, we obtain from (38)

Sx(f,Ns,Ls)=892γ2Δf∑i=−∞∞δ(f−iΔf)·∑(k,m,n)∈Si(k′,m′,n′)∈Si[Rs3Δf2[(a1+2Re{a1′})Pδk−k′δm−m′δn−n′+(a2+2Re{a2′})Pδk−k′δm+n′δn+m′+(a3+2Re{a3′})Pδk+nδm−m′δk′+n′]+Rs2Δf3[(b1+2Re{b1′})Pδk−m−k′δn+m′−n′+(b2P+2Re{b2′P})δk−m+m′δn−k′−n′+(b2∗P+2Re{b3′P})δk+n−k′δm−m′+n′+(b4+2Re{b4′})Pδk+n+m′δm+k′+n′+(c1P+2Re{c1′P})δk+nδm+k′−m′+n′+(c2+2Re{c2′})Pδk−k′δm−n−m′+n′+(c3P+2Re{c3′P})δk+m′δm−n+k′+n′+(c4+2Re{c4′})Pδm−m′δk+n−k′−n′+(c5P+2Re{c5′P})δm+k′δk+n+m′−n′+(c6P+2Re{c6′P})δk′+n′δk−m+n+m′]+RsΔf4(d1+2Re{d1′})Pδk−m+n−k′+m′−n′],

where we have used the fact that the sets $D_{i}$ for i=1,2,3,4,7,9,11,14, are closed to the transformation in Proposition 3. Furthermore, we notice that the set pairs $\left(D_{5},D_{6}\right)$, $\left(D_{8},D_{13}\right)$, and $\left(D_{10},D_{12}\right)$ represent pairs of complementary sets under the transformation in Corollary 1; hence, their elements can be grouped. Consequently,

(40) Sx(f,Ns,Ls)=892γ2Δf∑i=−∞∞δ(f−iΔf)·Rs3Δf2Φ1Q1+Φ2Q2+Φ3Q3+Rs2Δf3Ψ1Q4+2Re{Ψ2Q5+Ψ3Q5∗}+Ψ4Q6+2Re{Λ1Q7+Λ2Q7∗}+Λ3Q8+2Re{Λ4Q9+Λ5Q9∗}+Λ6Q10+RsΔf4Ξ1Q11,

where

(41) $$Q_{l}≜\sum_{\begin{array}{c} \left(k,m,n\right)\in S_{i}\\ \left(k^{\prime },m^{\prime },n^{\prime }\right)\in S_{i} \end{array}}PD^{\left(l\right)}=\sum_{T_{l,i}}P\;l=1,2,\ldots ,11,$$

the coefficients $\Phi_{i}$, i=1,2,3, $\Psi_{i}$, i=1,…,4, $\Lambda_{i}$, i=1,…,6, and $\Xi_{1}$ in (40) are given in Table 6; the sets $S_{i}$ are defined in (19); and $D^{\left(l\right)}$ is the coset leaders highlighted in boldface in Table 5 and listed in Table 7 with their corresponding set D. Finally, the sets $T_{l,i}$ are defined as

$$T_{l,i}≜\left\{\left(k,m,n,k^{\prime },m^{\prime },n^{\prime }\right)\in {\left\{0,1,\ldots ,W-1\right\}}^{6}:\left(k,m,n\right)\in S_{i},\;\left(k^{\prime },m^{\prime },n^{\prime }\right)\in S_{i},\;D^{\left(l\right)}=1\right\}.$$

Note how, in the second equality of (41), we have accounted for the multiplication by $D^{\left(l\right)}$ by restricting the summation set to $T_{l,i}$.

## 8. Final Result

Equation (40) expresses the NLI PSD for a periodic signal of period $T=1/\Delta_{f}$ as a function of the statistical moments and cross-polarisation correlations of a generic 4D modulation format. To generalise this result to aperiodic signals, we take the same approach in [8,17], i.e., we let the period *T* go to infinity or, equivalently, $\Delta_{f}\to 0$ (see Figure 3).

The limit of (40) for $\Delta_{f}\to 0$ is a limit of a distribution (a Dirac’s delta comb) which is parametric in $\Delta_{f}$. To rigorously evaluate such a limit, we use Lemma 1 and Theorem 2 presented in the following. In particular, Theorem 2 presents the *key result* of this work.

**Lemma** **1** (Dimensionality of the sets $T_{l,i}$)**.** *The sets $T_{l,i}$, for l=1,2,3, for l=4,…,10, and for l=11 have dimensionalities 2–4, respectively, ∀i∈Z.*

**Proof.**  See Appendix H. □

**Theorem** **2** (Limit of the distribution $S_{x}\left(f,N_{s},L_{s}\right)$)**.** *For a generic aperiodic transmitted signal and a fibre transmission system like the one in Figure 2 and under the following assumptions:*

- i.i.d. sequence of zero-mean input DP-4D symbols $a_{n}$ for n∈Z (see Section 3.1)
- rectangular (or quasi-rectangular) spectrum of the transmitted pulse p(t) (see Section 3.1)
- first-order RP framework for the solution of the Manakov equation in (8)

*the NLI PSD ${\bar{S}}_{x}\left(f,N_{s},L_{s}\right)≜\lim_{\Delta_{f}\to 0}S_{x}\left(f,N_{s},L_{s}\right)$, where $S_{x}\left(f,N_{s},L_{s}\right)$ is given in (40), is*

(42) S¯x(f,Ns,Ls)=892γ2Rs3Φ1χ1(f)+Φ2χ2(f)+Φ3χ3(f)+Rs2Ψ1χ4(f)+2Re{Ψ2χ5(f)+Ψ3χ5∗(f)}+Ψ4χ6(f)+2Re{Λ1χ7(f)+Λ2χ7∗(f)}+Λ3χ8(f)+2Re{Λ4χ9(f)+Λ5χ9∗(f)}+Λ6χ10(f)+RsΞ1χ11(f),

*where the coefficients $\Phi_{i}$, i=1,2,3, $\Psi_{i}$, i=1,2,…,4, $\Lambda_{i}$, i=1,2,…,6, and $\Xi_{1}$ as well as the integrals $\chi_{i}\left(f\right)$, i=1,2,…,11, are given in Table 8. As discussed at the end of Section 4, ${\bar{S}}_{y}\left(f\right)$ can be obtained applying the transformation x→y, y→x to (42).*

*The NLI power vector $\Sigma_{\mathrm{NLI}}$ can be obtained from the PSDs in x and y as*

(43) $$\Sigma_{\mathrm{NLI}}≜{\left[\sigma_{\mathrm{NLI},x}^{2},\sigma_{\mathrm{NLI},y}^{2}\right]}^{T}={\left[\int_{-\infty }^{\infty }{\bar{S}}_{x}\left(f,N_{s},L_{s}\right)|P\left(f\right){|}^{2}df,\int_{-\infty }^{\infty }{\bar{S}}_{y}\left(f,N_{s},L_{s}\right){\left|P\left(f\right)\right|}^{2}df\right]}^{T},$$

*where P(f) is the transmitted pulse spectrum.*

**Proof.**  See Appendix I. □

The results in (42) and (43) generalise the formulas presented in [9] for PM-2D modulation formats. In particular, assuming that

1. $a_{x}$ and $a_{y}$ are statistically independent, which leads to, e.g., $E\left\{a_{x}a_{y}\right\}=E\left\{a_{x}\right\}E\left\{a_{y}\right\}=0$,
2. $a_{x}$ and $a_{y}$ are identically distributed, which, for example, leads to ${E\{|a|}^{2}\}≜E\{|a_{x}{|}^{2}\}=E\{|a_{y}{|}^{2}\}$,
3. $E\left\{a_{x}^{2}\right\}=E\left\{a_{x}^{3}\right\}=E\left\{a_{y}^{2}\right\}=E\left\{a_{y}^{3}\right\}=0$, which applies, for instance, to distributions with a certain degree of symmetry,

it can be seen that (42) reduces to

$${\bar{S}}_{x}\left(f,N_{s},L_{s}\right)={\left(\frac{8}{9}\right)}^{2}\gamma^{2}\left[R_{s}^{3}\Phi_{1}\chi_{1}\left(f\right)+R_{s}^{2}\left(\Lambda_{3}\chi_{8}\left(f\right)+\Lambda_{6}\chi_{10}\left(f\right)\right)+R_{s}\Xi_{1}\chi_{11}\left(f\right)\right],$$

with

$$\begin{array}{cc} \Phi_{1} & =3E^{3}{\left\{|a\right|}^{2}\},\\ \Lambda_{3} & ={5E\{|a|}^{4}{\}E\{|a|}^{2}\}-10E^{3}{\left\{|a\right|}^{2}\},\\ \Lambda_{6} & ={E\{|a|}^{4}{\}E\{|a|}^{2}\}-2E^{3}{\left\{|a\right|}^{2}\},\\ \Xi_{1} & ={E\{|a|}^{6}{\}-9E\{|a|}^{4}{\}E\{|a|}^{2}\}+12E^{3}{\left\{|a\right|}^{2}\}, \end{array}$$

which matches the formulation given in [9] (Equation (41)).

**Table 8:** Table of high-order moments, correlation coefficients, and integrals appearing in (42). The function $\eta (f_{1},f_{2},f)$ is defined in (12).

Name	Value
**Correlation coefficients**	
$\Phi_{1}$	$2E^{3}\left\{\right|a_{x}{|}^{2}\}+4E\{|a_{x}{|}^{2}\}|E\left\{a_{x}a_{y}^{*}\right\}{|}^{2}+E\{|a_{x}{|}^{2}\}E^{2}\left\{\right|a_{y}{|}^{2}\}+|E\left\{a_{x}a_{y}^{*}\right\}{|}^{2}E\{|a_{y}{|}^{2}\}$
$\Phi_{2}$	$4E\{|a_{x}{|}^{2}\}|E\left\{a_{x}^{2}\right\}{|}^{2}+E\{|a_{x}{|}^{2}\}|E\left\{a_{y}^{2}\right\}{|}^{2}+4E\{|a_{x}{|}^{2}\}|E\left\{a_{x}a_{y}\right\}{|}^{2}+|E\left\{a_{x}a_{y}\right\}{|}^{2}E\{|a_{y}{|}^{2}\}$ +2Re{E{axay}E{ax∗ay}E∗{ay2}+2E∗{ax2}E{axay}E{axay∗}}
$\Phi_{3}$	E{|ax|2}|E{ax2}|2+|E{axay}|2E{|ay|2}+2Re{E{ax2}E∗{axay}E{ax∗ay}}
$\Psi_{1}$	$4|E\{a_{x}|a_{x}{|}^{2}{\}|}^{2}+4|E\{|a_{x}{|}^{2}a_{y}{\}|}^{2}+E\{|a_{x}{|}^{2}a_{y}\}E\{a_{y}^{*}|a_{y}{|}^{2}\}+E\{|a_{x}{|}^{2}a_{y}^{*}\}E\{a_{y}|a_{y}{|}^{2}\}+|E\{a_{x}|a_{y}{|}^{2}{\}|}^{2}$ +|E{ax∗ay2}|2+2Re{E{ax∗|ax|2}E{ax|ay|2}}
$\Psi_{2}$	$2|E\{a_{x}|a_{x}{|}^{2}{\}|}^{2}+2|E\{|a_{x}{|}^{2}a_{y}{\}|}^{2}+E\{|a_{x}{|}^{2}a_{y}^{*}\}E\{a_{y}|a_{y}{|}^{2}\}+|E\{a_{x}|a_{y}{|}^{2}{\}|}^{2}$
$\Psi_{3}$	$E\{a_{x}^{*}|a_{x}{|}^{2}\}E\{a_{x}|a_{y}{|}^{2}\}+|E\left\{a_{x}^{2}a_{y}^{*}\right\}{|}^{2}$
$\Psi_{4}$	$|E\left\{a_{x}^{3}\right\}{|}^{2}+2|E\left\{a_{x}^{2}a_{y}\right\}{|}^{2}+{\left|E\left\{a_{x}a_{y}^{2}\right\}\right|}^{2}$
$\Lambda_{1}$	$-3E\{|a_{x}{|}^{2}\}|E\left\{a_{x}^{2}\right\}{|}^{2}+E^{*}\{a_{x}^{2}|a_{x}{|}^{2}\}E\left\{a_{x}^{2}\right\}-|E\left\{a_{x}^{2}\right\}{|}^{2}E\{|a_{y}{|}^{2}\}-2|E\left\{a_{x}a_{y}\right\}{|}^{2}E\{|a_{y}{|}^{2}\}$ $+E\left\{a_{x}^{2}\right\}E^{*}\{a_{x}^{2}|a_{y}{|}^{2}\}-2E\left\{a_{x}^{2}\right\}E^{*}\left\{a_{x}a_{y}\right\}E\left\{a_{x}^{*}a_{y}\right\}+E\left\{a_{x}a_{y}\right\}E^{*}\{a_{x}a_{y}|a_{y}{|}^{2}\}-E\left\{a_{x}a_{y}\right\}E\left\{a_{x}^{*}a_{y}\right\}E^{*}\left\{a_{y}^{2}\right\}$
$\Lambda_{2}$	$-2E\{|a_{x}{|}^{2}\}|E\left\{a_{x}a_{y}\right\}{|}^{2}+E\left\{a_{x}a_{y}\right\}E^{*}\{a_{x}a_{y}|a_{x}{|}^{2}\}-E\left\{a_{x}^{2}\right\}E^{*}\left\{a_{x}a_{y}\right\}E\left\{a_{x}^{*}a_{y}\right\}$
$\Lambda_{3}$	$4E\{|a_{x}{|}^{4}\}E\{|a_{x}{|}^{2}\}-4E\{|a_{x}{|}^{2}\}|E\left\{a_{x}^{2}\right\}{|}^{2}-8E^{3}\left\{\right|a_{x}{|}^{2}\}+4E\{|a_{x}{|}^{2}\}E\{|a_{x}{|}^{2}|a_{y}{|}^{2}\}$ $-12E\{|a_{x}{|}^{2}\}|E\left\{a_{x}a_{y}^{*}\right\}{|}^{2}-4E\{|a_{x}{|}^{2}\}|E\left\{a_{x}a_{y}\right\}{|}^{2}-4E^{2}\left\{\right|a_{x}{|}^{2}\}E\{|a_{y}{|}^{2}\}-3E\{|a_{x}{|}^{2}\}E^{2}\left\{\right|a_{y}{|}^{2}\}$ $-E\{|a_{x}{|}^{2}\}|E\left\{a_{y}^{2}\right\}{|}^{2}+E\{|a_{x}{|}^{2}|a_{y}{|}^{2}\}E\{|a_{y}{|}^{2}\}+E\{|a_{x}{|}^{2}\}E\{|a_{y}{|}^{4}\}-5|E\left\{a_{x}a_{y}^{*}\right\}{|}^{2}E\{|a_{y}{|}^{2}\}$ −|E{axay}|2E{|ay|2}+2Re{2E{axay∗}E{ax∗ay|ax|2}−E{axay}E{ax∗ay}E∗{ay2} $+E\left\{a_{x}^{*}a_{y}\right\}E\{a_{x}a_{y}^{*}|a_{y}{|}^{2}\}-2E^{*}\left\{a_{x}^{2}\right\}E\left\{a_{x}a_{y}\right\}E\left\{a_{x}a_{y}^{*}\right\}\}$
$\Lambda_{4}$	$-6E\{|a_{x}{|}^{2}\}|E\left\{a_{x}^{2}\right\}{|}^{2}+2E^{*}\{a_{x}^{2}|a_{x}{|}^{2}\}E\left\{a_{x}^{2}\right\}+4E\{|a_{x}{|}^{2}\}|E\left\{a_{x}a_{y}\right\}{|}^{2}-E\{|a_{x}{|}^{2}\}|E\left\{a_{y}^{2}\right\}{|}^{2}$ $+E^{*}\left\{\right|a_{x}{|}^{2}a_{y}^{2}\}E\left\{a_{y}^{2}\right\}+2E\left\{a_{x}a_{y}\right\}E^{*}\{a_{x}|a_{x}{|}^{2}a_{y}\}-2|E\left\{a_{x}a_{y}\right\}{|}^{2}E\{|a_{y}{|}^{2}\}-2E^{*}\left\{a_{x}^{2}\right\}E\left\{a_{x}a_{y}\right\}E\left\{a_{x}a_{y}^{*}\right\}$ +E{axay}E∗{axay|ay|2}−E∗{axay}E{axay∗}E{ay2}−2Re{E∗{axay}E{axay∗}E{ay2}}
$\Lambda_{5}$	$-2E\{|a_{x}{|}^{2}\}|E\left\{a_{x}a_{y}\right\}{|}^{2}+E\left\{a_{x}a_{y}\right\}E^{*}\{a_{x}a_{y}|a_{x}{|}^{2}\}-|E\left\{a_{x}^{2}\right\}{|}^{2}E\{|a_{y}{|}^{2}\}-E^{*}\left\{a_{x}^{2}\right\}E\left\{a_{x}a_{y}\right\}E\left\{a_{x}a_{y}^{*}\right\}$ −2Re{E{ax2}E∗{axay}E{ax∗ay}}
$\Lambda_{6}$	$-2E^{3}\left\{\right|a_{x}{|}^{2}\}+E\{|a_{x}{|}^{4}\}E\{|a_{x}{|}^{2}\}-E\{|a_{x}{|}^{2}\}|E\left\{a_{x}^{2}\right\}{|}^{2}-4E\{|a_{x}{|}^{2}\}|E\left\{a_{x}a_{y}^{*}\right\}{|}^{2}-E\{|a_{x}{|}^{2}\}E^{2}\left\{\right|a_{y}{|}^{2}\}$ $+E\{|a_{x}{|}^{2}|a_{y}{|}^{2}\}E\{|a_{y}{|}^{2}\}-|E\left\{a_{x}a_{y}^{*}\right\}{|}^{2}E\{|a_{y}{|}^{2}\}-|E\left\{a_{x}a_{y}\right\}{|}^{2}E\{|a_{y}{|}^{2}\}$ +2Re{E{axay∗}E{ax∗ay|ax|2}−E{ax2}E∗{axay}E{ax∗ay}}
$\Xi_{1}$	$E\{|a_{x}{|}^{6}\}-9E\{|a_{x}{|}^{4}\}E\{|a_{x}{|}^{2}\}+12E^{3}\left\{\right|a_{x}{|}^{2}\}-2E\{|a_{x}{|}^{4}\}E\{|a_{y}{|}^{2}\}+E\{|a_{x}{|}^{2}|a_{y}{|}^{4}\}$ $-8E\{|a_{x}{|}^{2}\}E\{|a_{x}{|}^{2}|a_{y}{|}^{2}\}-4E\{|a_{x}{|}^{2}|a_{y}{|}^{2}\}E\{|a_{y}{|}^{2}\}+2E\{|a_{x}{|}^{4}|a_{y}{|}^{2}\}-E\{|a_{x}{|}^{2}\}E\{|a_{y}{|}^{4}\}$ $+4E\{|a_{x}{|}^{2}\}E^{2}\left\{\right|a_{y}{|}^{2}\}+8E^{2}\left\{\right|a_{x}{|}^{2}\}E\{|a_{y}{|}^{2}\}+18E\{|a_{x}{|}^{2}\}|E\left\{a_{x}^{2}\right\}{|}^{2}-|E\left\{a_{x}^{3}\right\}{|}^{2}-9|E\{a_{x}|a_{x}{|}^{2}{\}|}^{2}$ $+2E\{|a_{x}{|}^{2}\}|E\left\{a_{y}^{2}\right\}{|}^{2}-4|E\{a_{x}|a_{y}{|}^{2}{\}|}^{2}-8|E\{|a_{x}{|}^{2}a_{y}{\}|}^{2}+8|E\left\{a_{x}a_{y}^{*}\right\}{|}^{2}E\{|a_{y}{|}^{2}\}+8|E\left\{a_{x}a_{y}\right\}{|}^{2}E\{|a_{y}{|}^{2}\}$ $-|E\left\{a_{x}a_{y}^{2}\right\}{|}^{2}-|E\left\{a_{x}^{*}a_{y}^{2}\right\}{|}^{2}+16E\{|a_{x}{|}^{2}\}|E\left\{a_{x}a_{y}^{*}\right\}{|}^{2}-2|E\left\{a_{x}^{2}a_{y}^{*}\right\}{|}^{2}+16E\{|a_{x}{|}^{2}\}|E\left\{a_{x}a_{y}\right\}{|}^{2}$ +4|E{ax2}|2E{|ay|2}−2|E{ax2ay}|2+2Re{4E{axay}E{ax∗ay}E∗{ay2}−3E{ax2|ax|2}E∗{ax2} $-2E\{|a_{x}{|}^{2}a_{y}\}E\{a_{y}^{*}|a_{y}{|}^{2}\}-E\{|a_{x}{|}^{2}a_{y}^{2}\}E^{*}\left\{a_{y}^{2}\right\}-2E\left\{a_{x}a_{y}\right\}E^{*}\{a_{x}a_{y}|a_{y}{|}^{2}\}-E\left\{a_{x}a_{y}^{*}\right\}E\{a_{x}^{*}a_{y}|a_{y}{|}^{2}\}$ $-2E\{a_{x}^{*}|a_{x}{|}^{2}\}E\{a_{x}|a_{y}{|}^{2}\}-2E\left\{a_{x}^{2}\right\}E^{*}\{a_{x}^{2}|a_{y}{|}^{2}\}-E\{a_{x}|a_{x}{|}^{2}\}E\{a_{x}|a_{y}{|}^{2}\}-4E\left\{a_{x}a_{y}^{*}\right\}E\{a_{x}^{*}a_{y}|a_{x}{|}^{2}\}$ $-4E\left\{a_{x}a_{y}\right\}E^{*}\{a_{x}a_{y}|a_{x}{|}^{2}\}+8E\left\{a_{x}^{2}\right\}E^{*}\left\{a_{x}a_{y}\right\}E\left\{a_{x}^{*}a_{y}\right\}\}$
**Integrals**	
$\chi_{1}\left(f\right)$	$\int_{-R_{s}/2}^{R_{s}/2}\int_{-R_{s}/2}^{R_{s}/2}|P\left(f_{1}\right){|}^{2}|P\left(f_{2}\right){|}^{2}|P\left(f-f_{1}+f_{2}\right){|}^{2}{\left|\eta \left(f_{1},f_{2},f\right)\right|}^{2}df_{1}\;df_{2}$
$\chi_{2}\left(f\right)$	$\int_{-R_{s}/2}^{R_{s}/2}\int_{-R_{s}/2}^{R_{s}/2}|P\left(f_{1}\right){|}^{2}|P\left(f_{2}\right){|}^{2}{\left|P\left(f-f_{1}+f_{2}\right)\right|}^{2}\eta \left(f_{1},f_{2},f\right)\eta^{*}\left(f_{1},f_{1}-f_{2}-f,f\right)df_{1}\;df_{2}$
$\chi_{3}\left(f\right)$	${\left|P\left(f\right)\right|}^{2}\int_{-R_{s}/2}^{R_{s}/2}\int_{-R_{s}/2}^{R_{s}/2}|P\left(f_{1}\right){|}^{2}{\left|P\left(f_{2}\right)\right|}^{2}\eta \left(f_{1},-f,f\right)\eta^{*}\left(f_{2},-f,f\right)df_{1}\;df_{2}$
$\chi_{4}\left(f\right)$	$\int_{-R_{s}/2}^{R_{s}/2}\int_{-R_{s}/2}^{R_{s}/2}\int_{-R_{s}/2}^{R_{s}/2}P\left(f_{1}\right)P^{*}\left(f_{2}\right)P\left(f-f_{1}+f_{2}\right)P^{*}\left(f_{1}-f_{2}\right)P\left(f_{3}\right)P^{*}\left(f-f_{1}+f_{2}+f_{3}\right)\eta \left(f_{1},f_{2},f\right)$ $\cdot \eta^{*}\left(f_{1}-f_{2},f_{3},f\right)df_{1}\;df_{2}\;df_{3}$
$\chi_{5}\left(f\right)$	$\int_{-R_{s}/2}^{R_{s}/2}\int_{-R_{s}/2}^{R_{s}/2}\int_{-R_{s}/2}^{R_{s}/2}P\left(f_{1}\right)P^{*}\left(f_{2}\right)P\left(f-f_{1}+f_{2}\right)P^{*}\left(f_{3}\right)P\left(f_{2}-f_{1}\right)P^{*}\left(f-f_{1}+f_{2}-f_{3}\right)\eta \left(f_{1},f_{2},f\right)$ $\cdot \eta^{*}\left(f_{3},f_{2}-f_{1},f\right)df_{1}\;df_{2}\;df_{3}$
$\chi_{6}\left(f\right)$	$\int_{-R_{s}/2}^{R_{s}/2}\int_{-R_{s}/2}^{R_{s}/2}\int_{-R_{s}/2}^{R_{s}/2}P\left(f_{1}\right)P^{*}\left(f_{2}\right)P\left(f-f_{1}+f_{2}\right)P^{*}\left(f_{3}\right)P^{*}\left(f+f_{2}\right)P\left(f_{2}+f_{3}\right)\eta \left(f_{1},f_{2},f\right)$ $\cdot \eta^{*}\left(f_{3},-f-f_{2},f\right)df_{1}\;df_{2}\;df_{3}$
$\chi_{7}\left(f\right)$	$P\left(f\right)\int_{-R_{s}/2}^{R_{s}/2}\int_{-R_{s}/2}^{R_{s}/2}\int_{-R_{s}/2}^{R_{s}/2}{\left|P\left(f_{1}\right)\right|}^{2}P^{*}\left(f_{2}\right)P\left(f_{3}\right)P^{*}\left(f-f_{2}+f_{3}\right)\eta \left(f_{1},-f,f\right)\eta^{*}\left(f_{2},f_{3},f\right)df_{1}\;df_{2}\;df_{3}$
$\chi_{8}\left(f\right)$	$\int_{-R_{s}/2}^{R_{s}/2}\int_{-R_{s}/2}^{R_{s}/2}\int_{-R_{s}/2}^{R_{s}/2}{\left|P\left(f_{1}\right)\right|}^{2}P^{*}\left(f_{2}\right)P\left(f-f_{1}+f_{2}\right)P\left(f_{3}\right)P^{*}\left(f-f_{1}+f_{3}\right)\eta \left(f_{1},f_{2},f\right)\eta^{*}\left(f_{1},f_{3},f\right)df_{1}\;df_{2}\;df_{3}$
$\chi_{9}\left(f\right)$	$\int_{-R_{s}/2}^{R_{s}/2}\int_{-R_{s}/2}^{R_{s}/2}\int_{-R_{s}/2}^{R_{s}/2}{\left|P\left(f_{1}\right)\right|}^{2}P^{*}\left(f_{2}\right)P\left(f-f_{1}+f_{2}\right)P^{*}\left(f_{3}\right)P\left(f-f_{1}-f_{3}\right)\eta \left(f_{1},f_{2},f\right)\eta^{*}\left(f_{3},-f_{1},f\right)df_{1}\;df_{2}\;df_{3}$
$\chi_{10}\left(f\right)$	$\int_{-R_{s}/2}^{R_{s}/2}\int_{-R_{s}/2}^{R_{s}/2}\int_{-R_{s}/2}^{R_{s}/2}P\left(f_{1}\right){\left|P\left(f_{2}\right)\right|}^{2}P\left(f-f_{1}+f_{2}\right)P^{*}\left(f_{3}\right)P^{*}\left(f+f_{2}-f_{3}\right)\eta \left(f_{1},f_{2},f\right)\eta^{*}\left(f_{3},f_{2},f\right)df_{1}\;df_{2}\;df_{3}$
$\chi_{11}\left(f\right)$	$\int_{-R_{s}/2}^{R_{s}/2}\int_{-R_{s}/2}^{R_{s}/2}\int_{-R_{s}/2}^{R_{s}/2}\int_{-R_{s}/2}^{R_{s}/2}P\left(f_{1}\right)P^{*}\left(f_{2}\right)P\left(f-f_{1}+f_{2}\right)P^{*}\left(f_{3}\right)P\left(f_{4}\right)P^{*}\left(f-f_{3}+f_{4}\right)\eta \left(f_{1},f_{2},f\right)$ $\cdot \eta^{*}\left(f_{3},f_{4},f\right)df_{1}\;df_{2}\;df_{3}\;df_{4}$

## 9. Discussion and Conclusions

In this paper, we have derived a comprehensive analytical expression for NLI power when a general DP-4D modulation format is transmitted. The transmitted format is only assumed to be zero-mean. The reported result extends the model in [9] by accounting for any constellation geometry and statistic in four dimensions. This extension is performed by lifting two underlying assumptions in [9] (and other existing models): (i) the transmitted formats are PM versions of a 2D format and (ii) some high-order moments of the 2D components of the transmitted modulation format, such as $E\{a_{x}^{2}\}$ and $E\{a_{x}^{3}\}$, are implicitly assumed to be equal to zero.

The presented results are derived in a single-channel transmission scenario. However, as it can be inferred from previous works, extending the expressions to the wavelength-division multiplexing (WDM) case does not lead to a different set of modulation-related statistical quantities in the NLI power expression. An extension of this work to the WDM transmission scenario will be addressed in a future publication.

Future work will also focus on comparing the presented model with possible heuristic extensions of existing PM-2D models to the general DP-4D case, for instance by using the 4D constellation standardised fourth-order moment (or so-called kurtosis). For such a study, a numerical validation of the model via the split-step Fourier method will certainly play a key role. Lastly, 4D constellation shaping in the optical fibre channel arguably represents the most attractive application and future research direction for the model derived in this manuscript. 

## Figures and Tables

**Table 1:** List of contributions $M_{1}^{\left(h\right)}$ and $N_{1}^{\left(h\right)}$ for i=1,2,…,15.

*h*	Corr. Terms in $M_{1}^{\left(h\right)}$	Corr. Terms in $N_{1}^{\left(h\right)}$	Delta Products
1	$E^{3}\left\{\right|a_{x}{|}^{2}\}$ $+|E\left\{a_{x}a_{y}^{*}\right\}{|}^{2}E\{|a_{y}{|}^{2}\}$	$E\{|a_{x}{|}^{2}\}|E\left\{a_{x}a_{y}^{*}\right\}{|}^{2}$	$R_{s}^{3}\delta_{k-m}\delta_{n-k^{\prime }}\delta_{m^{\prime }-n^{\prime }}-R_{s}^{2}\Delta_{f}(\delta_{m^{\prime }-n^{\prime }}\delta_{k-m+n-k^{\prime }}$ $+\delta_{n-k^{\prime }}\delta_{k-m+m^{\prime }-n^{\prime }}+\delta_{k-m}\delta_{m^{\prime }-n^{\prime }+n-k^{\prime }})$ $+2R_{s}\Delta_{f}^{2}\delta_{k-m+n-k^{\prime }+m^{\prime }-n^{\prime }}$
2	$E\{|a_{x}{|}^{2}\}|E\left\{a_{x}^{2}\right\}{|}^{2}$ $+|E\left\{a_{x}a_{y}\right\}{|}^{2}E\{|a_{y}{|}^{2}\}$	$E\{|a_{x}{|}^{2}\}|E\left\{a_{x}a_{y}\right\}{|}^{2}$	$R_{s}^{3}\delta_{k-m}\delta_{n+m^{\prime }}\delta_{k^{\prime }+n^{\prime }}-R_{s}^{2}\Delta_{f}(\delta_{k^{\prime }+n^{\prime }}\delta_{k-m+n+m^{\prime }}$ $+\delta_{n+m^{\prime }}\delta_{k-m-k^{\prime }-n^{\prime }}+\delta_{k-m}\delta_{n-k^{\prime }+m^{\prime }-n^{\prime }})$ $+2R_{s}\Delta_{f}^{2}\delta_{k-m+n-k^{\prime }+m^{\prime }-n^{\prime }}$
3	$E^{3}\left\{\right|a_{x}{|}^{2}\}$ $+E\{|a_{x}{|}^{2}\}E^{2}\left\{\right|a_{y}{|}^{2}\}$	$E^{2}\left\{\right|a_{x}{|}^{2}\}E\{|a_{y}{|}^{2}\}$	$R_{s}^{3}\delta_{k-m}\delta_{n-n^{\prime }}\delta_{k^{\prime }-m^{\prime }}-R_{s}^{2}\Delta_{f}(\delta_{k^{\prime }-m^{\prime }}\delta_{k-m+n-n^{\prime }}$ $+\delta_{n-n^{\prime }}\delta_{k-m-k^{\prime }+m^{\prime }}+\delta_{k-m}\delta_{n-n^{\prime }-k^{\prime }+m^{\prime }})$ $+2R_{s}\Delta_{f}^{2}\delta_{k-m+n-k^{\prime }+m^{\prime }-n^{\prime }}$
4	$E\{|a_{x}{|}^{2}\}|E\left\{a_{x}^{2}\right\}{|}^{2}$ $+E\left\{a_{x}a_{y}\right\}E\left\{a_{x}^{*}a_{y}\right\}E^{*}\left\{a_{y}^{2}\right\}$	$E\left\{a_{x}^{2}\right\}E^{*}\left\{a_{x}a_{y}\right\}E\left\{a_{x}^{*}a_{y}\right\}$	$R_{s}^{3}\delta_{k+n}\delta_{m+k^{\prime }}\delta_{m^{\prime }-n^{\prime }}-R_{s}^{2}\Delta_{f}(\delta_{m^{\prime }-n^{\prime }}\delta_{k+n-m-k^{\prime }}$ $+\delta_{m+k^{\prime }}\delta_{k+n+m^{\prime }-n^{\prime }}+\delta_{k+n}\delta_{m+k^{\prime }-m^{\prime }+n^{\prime }})$ $+2R_{s}\Delta_{f}^{2}\delta_{k-m+n-k^{\prime }+m^{\prime }-n^{\prime }}$
5	$E\{|a_{x}{|}^{2}\}|E\left\{a_{x}^{2}\right\}{|}^{2}$ $\cdot |E\left\{a_{x}a_{y}\right\}{|}^{2}E\{|a_{y}{|}^{2}\}$	$E\left\{a_{x}^{2}\right\}E^{*}\left\{a_{x}a_{y}\right\}E\left\{a_{x}^{*}a_{y}\right\}$	$R_{s}^{3}\delta_{k+n}\delta_{m-m^{\prime }}\delta_{k^{\prime }+n^{\prime }}-R_{s}^{2}\Delta_{f}(\delta_{k^{\prime }+n^{\prime }}\delta_{k+n-m+m^{\prime }}$ $+\delta_{m-m^{\prime }}\delta_{k+n-k^{\prime }-n^{\prime }}+\delta_{k+n}\delta_{m-m^{\prime }+k^{\prime }+n^{\prime }})$ $+2R_{s}\Delta_{f}^{2}\delta_{k-m+n-k^{\prime }+m^{\prime }-n^{\prime }}$
6	$E\{|a_{x}{|}^{2}\}|E\left\{a_{x}^{2}\right\}{|}^{2}$ $+|E\left\{a_{x}a_{y}\right\}{|}^{2}E\{|a_{y}{|}^{2}\}$	$|E\left\{a_{x}^{2}\right\}{|}^{2}E\{|a_{y}{|}^{2}\}$	$R_{s}^{3}\delta_{k+n}\delta_{m+n^{\prime }}\delta_{k^{\prime }-m^{\prime }}-R_{s}^{2}\Delta_{f}(\delta_{k^{\prime }-m^{\prime }}\delta_{k+n-m-n^{\prime }}$ $+\delta_{m+n^{\prime }}\delta_{k+n-k^{\prime }+m^{\prime }}+\delta_{k+n}\delta_{m+k^{\prime }-m^{\prime }+n^{\prime }})$ $+2R_{s}\Delta_{f}^{2}\delta_{k-m+n-k^{\prime }+m^{\prime }-n^{\prime }}$
7	$E^{3}\left\{\right|a_{x}{|}^{2}\}$ $+|E\left\{a_{x}a_{y}^{*}\right\}{|}^{2}E\{|a_{y}{|}^{2}\}$	$E\{|a_{x}{|}^{2}\}|E\left\{a_{x}a_{y}^{*}\right\}{|}^{2}$	$R_{s}^{3}\delta_{k-k^{\prime }}\delta_{m-n}\delta_{m^{\prime }-n^{\prime }}-R_{s}^{2}\Delta_{f}(\delta_{m^{\prime }-n^{\prime }}\delta_{k-k^{\prime }-m+n}$ $+\delta_{m-n}\delta_{k-k^{\prime }+m^{\prime }-n^{\prime }}+\delta_{k-k^{\prime }}\delta_{m-n-m^{\prime }+n^{\prime }})$ $+2R_{s}\Delta_{f}^{2}\delta_{k-m+n-k^{\prime }+m^{\prime }-n^{\prime }}$
8	$E^{3}\left\{\right|a_{x}{|}^{2}\}$ $+E\{|a_{x}{|}^{2}\}E^{2}\left\{\right|a_{y}{|}^{2}\}$	$E\{|a_{x}{|}^{2}\}|E\left\{a_{x}a_{y}^{*}\right\}{|}^{2}$	$R_{s}^{3}\delta_{k-k^{\prime }}\delta_{m-m^{\prime }}\delta_{n-n^{\prime }}-R_{s}^{2}\Delta_{f}(\delta_{n-n^{\prime }}\delta_{k-m-k^{\prime }+m^{\prime }}$ $+\delta_{m-m^{\prime }}\delta_{k+n-k^{\prime }-n^{\prime }}+\delta_{k-k^{\prime }}\delta_{m-n-m^{\prime }+n^{\prime }})$ $+2R_{s}\Delta_{f}^{2}\delta_{k-m+n-k^{\prime }+m^{\prime }-n^{\prime }}$
9	$E\{|a_{x}{|}^{2}\}|E\left\{a_{x}^{2}\right\}{|}^{2}$ $+|E\left\{a_{x}a_{y}\right\}{|}^{2}E\{|a_{y}{|}^{2}\}$	$E^{*}\left\{a_{x}^{2}\right\}E\left\{a_{x}a_{y}\right\}E\left\{a_{x}a_{y}^{*}\right\}$	$R_{s}^{3}\delta_{k-k^{\prime }}\delta_{m+n^{\prime }}\delta_{n+m^{\prime }}-R_{s}^{2}\Delta_{f}(\delta_{n+m^{\prime }}\delta_{k-m-k^{\prime }-n^{\prime }}$ $+\delta_{m+n^{\prime }}\delta_{k+n-k^{\prime }+m^{\prime }}+\delta_{k-k^{\prime }}\delta_{m-n-m^{\prime }+n^{\prime }})$ $+2R_{s}\Delta_{f}^{2}\delta_{k-m+n-k^{\prime }+m^{\prime }-n^{\prime }}$
10	$E\{|a_{x}{|}^{2}\}|E\left\{a_{x}^{2}\right\}{|}^{2}$ $+E^{*}\left\{a_{x}a_{y}\right\}E\left\{a_{x}a_{y}^{*}\right\}E\left\{a_{y}^{2}\right\}$	$E\{|a_{x}{|}^{2}\}|E\left\{a_{x}a_{y}\right\}{|}^{2}$	$R_{s}^{3}\delta_{k+m^{\prime }}\delta_{m-n}\delta_{k^{\prime }+n^{\prime }}-R_{s}^{2}\Delta_{f}(\delta_{k^{\prime }+n^{\prime }}\delta_{k-m+n+m^{\prime }}$ $+\delta_{m-n}\delta_{k-k^{\prime }+m^{\prime }-n^{\prime }}+\delta_{k+m^{\prime }}\delta_{m-n+k^{\prime }+n^{\prime }})$ $+2R_{s}\Delta_{f}^{2}\delta_{k-m+n-k^{\prime }+m^{\prime }-n^{\prime }}$
11	$E\{|a_{x}{|}^{2}\}|E\left\{a_{x}^{2}\right\}{|}^{2}$ $+E\{|a_{x}{|}^{2}\}|E\left\{a_{y}^{2}\right\}{|}^{2}$	$E\{|a_{x}{|}^{2}\}|E\left\{a_{x}a_{y}\right\}{|}^{2}$	$R_{s}^{3}\delta_{k+m^{\prime }}\delta_{m+k^{\prime }}\delta_{n-n^{\prime }}-R_{s}^{2}\Delta_{f}(\delta_{n-n^{\prime }}\delta_{k-m-k^{\prime }+m^{\prime }}$ $+\delta_{m+k^{\prime }}\delta_{k+n+m^{\prime }-n^{\prime }}+\delta_{k+m^{\prime }}\delta_{m-n+k^{\prime }+n^{\prime }})$ $+2R_{s}\Delta_{f}^{2}\delta_{k-m+n-k^{\prime }+m^{\prime }-n^{\prime }}$
12	$E\{|a_{x}{|}^{2}\}|E\left\{a_{x}^{2}\right\}{|}^{2}$ $+E^{*}\left\{a_{x}a_{y}\right\}E\left\{a_{x}a_{y}^{*}\right\}E\left\{a_{y}^{2}\right\}$	$E^{*}\left\{a_{x}^{2}\right\}E\left\{a_{x}a_{y}\right\}E\left\{a_{x}a_{y}^{*}\right\}$	$R_{s}^{3}\delta_{k+m^{\prime }}\delta_{m+n^{\prime }}\delta_{n-k^{\prime }}-R_{s}^{2}\Delta_{f}(\delta_{n-k^{\prime }}\delta_{k-m+m^{\prime }-n^{\prime }}$ $+\delta_{m+n^{\prime }}\delta_{k+n-k^{\prime }+m^{\prime }}+\delta_{k+m^{\prime }}\delta_{m-n+k^{\prime }+n^{\prime }})$ $+2R_{s}\Delta_{f}^{2}\delta_{k-m+n-k^{\prime }+m^{\prime }-n^{\prime }}$
13	$E^{3}\left\{\right|a_{x}{|}^{2}\}$ $+|E\left\{a_{x}a_{y}^{*}\right\}{|}^{2}E\{|a_{y}{|}^{2}\}$	$E^{2}\left\{\right|a_{x}{|}^{2}\}E\{|a_{y}{|}^{2}\}$	$R_{s}^{3}\delta_{k-n^{\prime }}\delta_{m-n}\delta_{k^{\prime }-m^{\prime }}-R_{s}^{2}\Delta_{f}(\delta_{k^{\prime }-m^{\prime }}\delta_{k-m+n-n^{\prime }}$ $+\delta_{m-n}\delta_{k-k^{\prime }+m^{\prime }-n^{\prime }}+\delta_{k-n^{\prime }}\delta_{m-n+k^{\prime }-m^{\prime }})$ $+2R_{s}\Delta_{f}^{2}\delta_{k-m+n-k^{\prime }+m^{\prime }-n^{\prime }}$
14	$E\{|a_{x}{|}^{2}\}|E\left\{a_{x}^{2}\right\}{|}^{2}$ $+E\left\{a_{x}a_{y}\right\}E{\{}_{x}a^{*}a_{y}\}E^{*}\left\{a_{y}^{2}\right\}$	$E\{|a_{x}{|}^{2}\}|E\left\{a_{x}a_{y}\right\}{|}^{2}$	$R_{s}^{3}\delta_{k-n^{\prime }}\delta_{m+k^{\prime }}\delta_{n+m^{\prime }}-R_{s}^{2}\Delta_{f}(\delta_{n+m^{\prime }}\delta_{k-m-k^{\prime }-n^{\prime }}$ $+\delta_{m+k^{\prime }}\delta_{k+n+m^{\prime }-n^{\prime }}+\delta_{k-n^{\prime }}\delta_{m-n+k^{\prime }-m^{\prime }})$ $+2R_{s}\Delta_{f}^{2}\delta_{k-m+n-k^{\prime }+m^{\prime }-n^{\prime }}$
15	$E^{3}\left\{\right|a_{x}{|}^{2}\}$ $+|E\left\{a_{x}a_{y}^{*}\right\}{|}^{2}E\{|a_{y}{|}^{2}\}$	$E\{|a_{x}{|}^{2}\}|E\left\{a_{x}a_{y}^{*}\right\}{|}^{2}$	$R_{s}^{3}\delta_{k-n^{\prime }}\delta_{m-m^{\prime }}\delta_{n-k^{\prime }}-R_{s}^{2}\Delta_{f}(\delta_{n-k^{\prime }}\delta_{k-m+m^{\prime }-n^{\prime }}$ $+\delta_{m-m^{\prime }}\delta_{k+n-k^{\prime }-n^{\prime }}+\delta_{k-n^{\prime }}\delta_{m-n+k^{\prime }-m^{\prime }})$ $+2R_{s}\Delta_{f}^{2}\delta_{k-m+n-k^{\prime }+m^{\prime }-n^{\prime }}$

**Table 2:** List of contributions $M_{2}^{\left(h\right)}$ and $N_{2}^{\left(h\right)}$ for i=1,2,…,10.

*h*	Corr. Terms in $M_{2}^{\left(h\right)}$	Corr. Terms in $N_{2}^{\left(h\right)}$	Delta Products
1	$|E\{a_{x}|a_{x}{|}^{2}{\}|}^{2}+|E\{a_{x}|a_{y}{|}^{2}{\}|}^{2}$	$E\{a_{x}|a_{x}{|}^{2}\}E\{a_{x}|a_{y}{|}^{2}\}$	$R_{s}^{2}\Delta_{f}\delta_{k-m+n}\delta_{k^{\prime }-m^{\prime }+n^{\prime }}$ $-R_{s}\Delta_{f}^{2}\delta_{k-m+n-k^{\prime }+m^{\prime }-n^{\prime }}$
2	$|E\{a_{x}|a_{x}{|}^{2}{\}|}^{2}+E\{a_{y}^{*}|a_{y}{|}^{2}\}E\{|a_{x}{|}^{2}a_{y}\}$	$\left|E\{\right|a_{x}{|}^{2}a_{y}{\}|}^{2}$	$R_{s}^{2}\Delta_{f}\delta_{k-m-k^{\prime }}\delta_{n+m^{\prime }-n^{\prime }}$ $-R_{s}\Delta_{f}^{2}\delta_{k-m+n-k^{\prime }+m^{\prime }-n^{\prime }}$
3	$|E\{a_{x}|a_{x}{|}^{2}{\}|}^{2}+E\{|a_{x}{|}^{2}a_{y}^{*}\}E\{a_{y}|a_{y}{|}^{2}\}$	$\left|E\{\right|a_{x}{|}^{2}a_{y}{\}|}^{2}$	$R_{s}^{2}\Delta_{f}\delta_{k-m+m^{\prime }}\delta_{n-k^{\prime }-n^{\prime }}$ $-R_{s}\Delta_{f}^{2}\delta_{k-m+n-k^{\prime }+m^{\prime }-n^{\prime }}$
4	$|E\{a_{x}|a_{x}{|}^{2}{\}|}^{2}+|E\{a_{x}|a_{y}{|}^{2}{\}|}^{2}$	$E\{a_{x}^{*}|a_{x}{|}^{2}\}E\{a_{x}|a_{y}{|}^{2}\}$	$R_{s}^{2}\Delta_{f}\delta_{k-m-n^{\prime }}\delta_{n-k^{\prime }+m^{\prime }}$ $-R_{s}\Delta_{f}^{2}\delta_{k-m+n-k^{\prime }+m^{\prime }-n^{\prime }}$
5	$|E\{a_{x}|a_{x}{|}^{2}{\}|}^{2}+|E\{a_{x}|a_{y}{|}^{2}{\}|}^{2}$	$|E\left\{a_{x}^{2}a_{y}^{*}\right\}{|}^{2}$	$R_{s}^{2}\Delta_{f}\delta_{k+n-k^{\prime }}\delta_{m-m^{\prime }+n^{\prime }}$ $-R_{s}\Delta_{f}^{2}\delta_{k-m+n-k^{\prime }+m^{\prime }-n^{\prime }}$
6	$|E\left\{a_{x}^{3}\right\}{|}^{2}+{\left|E\left\{a_{x}a_{y}^{2}\right\}\right|}^{2}$	$|E\left\{a_{x}^{2}a_{y}\right\}{|}^{2}$	$R_{s}^{2}\Delta_{f}\delta_{k+n+m^{\prime }}\delta_{m+k^{\prime }+n^{\prime }}$ $-R_{s}\Delta_{f}^{2}\delta_{k-m+n-k^{\prime }+m^{\prime }-n^{\prime }}$
7	$|E\{a_{x}|a_{x}{|}^{2}{\}|}^{2}+E\{|a_{x}{|}^{2}a_{y}\}E\{a_{y}^{*}|a_{y}{|}^{2}\}$	$E\{a_{x}|a_{x}{|}^{2}\}E\{a_{x}^{*}|a_{y}{|}^{2}\}$	$R_{s}^{2}\Delta_{f}\delta_{k+n-n^{\prime }}\delta_{m+k^{\prime }-m^{\prime }}$ $-R_{s}\Delta_{f}^{2}\delta_{k-m+n-k^{\prime }+m^{\prime }-n^{\prime }}$
8	$|E\{a_{x}|a_{x}{|}^{2}{\}|}^{2}+E\{|a_{x}{|}^{2}a_{y}^{*}\}E\{a_{y}|a_{y}{|}^{2}\}$	$E\{a_{x}|a_{y}{|}^{2}\}E\{a_{x}^{*}|a_{x}{|}^{2}\}$	$R_{s}^{2}\Delta_{f}\delta_{k-k^{\prime }+m^{\prime }}\delta_{m-n+n^{\prime }}$ $-R_{s}\Delta_{f}^{2}\delta_{k-m+n-k^{\prime }+m^{\prime }-n^{\prime }}$
9	$|E\{a_{x}|a_{x}{|}^{2}{\}|}^{2}+|E\{a_{x}|a_{y}{|}^{2}{\}|}^{2}$	$\left|E\{\right|a_{x}{|}^{2}a_{y}{\}|}^{2}$	$R_{s}^{2}\Delta_{f}\delta_{k-k^{\prime }-n^{\prime }}\delta_{m-n-m^{\prime }}$ $-R_{s}\Delta_{f}^{2}\delta_{k-m+n-k^{\prime }+m^{\prime }-n^{\prime }}$
10	$|E\{a_{x}|a_{x}{|}^{2}{\}|}^{2}+{\left|E\left\{a_{x}^{*}a_{y}^{2}\right\}\right|}^{2}$	$\left|E\{\right|a_{x}{|}^{2}a_{y}{\}|}^{2}$	$R_{s}^{2}\Delta_{f}\delta_{k+m^{\prime }-n^{\prime }}\delta_{m-n+k^{\prime }}$ $-R_{s}\Delta_{f}^{2}\delta_{k-m+n-k^{\prime }+m^{\prime }-n^{\prime }}$

**Table 3:** List of contributions $M_{3}^{\left(h\right)}$ and $N_{3}^{\left(h\right)}$ for i=1,2,…,15.

*h*	Corr. Terms in $M_{3}^{\left(h\right)}$	Corr. Terms in $N_{3}^{\left(h\right)}$	Delta Products
1	$E\{|a_{x}{|}^{4}\}E\{|a_{x}{|}^{2}\}+E\{|a_{x}{|}^{2}|a_{y}{|}^{2}\}E\{|a_{y}{|}^{2}\}$	$E\{|a_{x}{|}^{2}\}E\{|a_{x}{|}^{2}|a_{y}{|}^{2}\}$	$R_{s}^{2}\Delta_{f}\delta_{k-m}\delta_{n-k^{\prime }+m^{\prime }-n^{\prime }}$ $-R_{s}\Delta_{f}^{2}\delta_{k-m+n-k^{\prime }+m^{\prime }-n^{\prime }}$
2	$E^{*}\{a_{x}^{2}|a_{x}{|}^{2}\}E\left\{a_{x}^{2}\right\}+E\left\{a_{x}a_{y}\right\}E^{*}\{a_{x}a_{y}|a_{y}{|}^{2}\}$	$E\left\{a_{x}^{2}\right\}E^{*}\{a_{x}^{2}|a_{y}{|}^{2}\}$	$R_{s}^{2}\Delta_{f}\delta_{k+n}\delta_{m+k^{\prime }-m^{\prime }+n^{\prime }}$ $-R_{s}\Delta_{f}^{2}\delta_{k-m+n-k^{\prime }+m^{\prime }-n^{\prime }}$
3	$E\{|a_{x}{|}^{4}\}E\{|a_{x}{|}^{2}\}+E\{|a_{x}{|}^{2}|a_{y}{|}^{2}\}E\{|a_{y}{|}^{2}\}$	$E\left\{a_{x}a_{y}^{*}\right\}E\{a_{x}^{*}a_{y}|a_{x}{|}^{2}\}$	$R_{s}^{2}\Delta_{f}\delta_{k-k^{\prime }}\delta_{m-n-m^{\prime }+n^{\prime }}$ $-R_{s}\Delta_{f}^{2}\delta_{k-m+n-k^{\prime }+m^{\prime }-n^{\prime }}$
4	$E^{*}\{a_{x}^{2}|a_{x}{|}^{2}\}E\left\{a_{x}^{2}\right\}+E^{*}\left\{\right|a_{x}{|}^{2}a_{y}^{2}\}E\left\{a_{y}^{2}\right\}$	$E\left\{a_{x}a_{y}\right\}E^{*}\{a_{x}a_{y}|a_{x}{|}^{2}\}$	$R_{s}^{2}\Delta_{f}\delta_{k+m^{\prime }}\delta_{m-n+k^{\prime }+n^{\prime }}$ $-R_{s}\Delta_{f}^{2}\delta_{k-m+n-k^{\prime }+m^{\prime }-n^{\prime }}$
5	$E\{|a_{x}{|}^{4}\}E\{|a_{x}{|}^{2}\}+E\left\{a_{x}^{*}a_{y}\right\}E\{a_{x}a_{y}^{*}|a_{y}{|}^{2}\}$	$E\{|a_{x}{|}^{2}\}E\{|a_{x}{|}^{2}|a_{y}{|}^{2}\}$	$R_{s}^{2}\Delta_{f}\delta_{k-n^{\prime }}\delta_{m-n+k^{\prime }-m^{\prime }}$ $-R_{s}\Delta_{f}^{2}\delta_{k-m+n-k^{\prime }+m^{\prime }-n^{\prime }}$
6	$E\{|a_{x}{|}^{4}\}E\{|a_{x}{|}^{2}\}+E\left\{a_{x}a_{y}^{*}\right\}E\{a_{x}^{*}a_{y}|a_{y}{|}^{2}\}$	$E\{|a_{x}{|}^{2}\}E\{|a_{x}{|}^{2}|a_{y}{|}^{2}\}$	$R_{s}^{2}\Delta_{f}\delta_{m-n}\delta_{k-k^{\prime }+m^{\prime }-n^{\prime }}$ $-R_{s}\Delta_{f}^{2}\delta_{k-m+n-k^{\prime }+m^{\prime }-n^{\prime }}$
7	$E\{a_{x}^{2}|a_{x}{|}^{2}\}E^{*}\left\{a_{x}^{2}\right\}+E\{|a_{x}{|}^{2}a_{y}^{2}\}E^{*}\left\{a_{y}^{2}\right\}$	$E^{*}\left\{a_{x}a_{y}\right\}E\{a_{x}a_{y}|a_{x}{|}^{2}\}$	$R_{s}^{2}\Delta_{f}\delta_{m+k^{\prime }}\delta_{k-n+m^{\prime }-n^{\prime }}$ $-R_{s}\Delta_{f}^{2}\delta_{k-m+n-k^{\prime }+m^{\prime }-n^{\prime }}$
8	$E\{|a_{x}{|}^{4}\}E\{|a_{x}{|}^{2}\}+E\{|a_{x}{|}^{2}|a_{y}{|}^{2}\}E\{|a_{y}{|}^{2}\}$	$E\left\{a_{x}^{*}a_{y}\right\}E\{a_{x}a_{y}^{*}|a_{x}{|}^{2}\}$	$R_{s}^{2}\Delta_{f}\delta_{m-m^{\prime }}\delta_{k+n-k^{\prime }-n^{\prime }}$ $-R_{s}\Delta_{f}^{2}\delta_{k-m+n-k^{\prime }+m^{\prime }-n^{\prime }}$
9	$E\{a_{x}^{2}|a_{x}{|}^{2}\}E^{*}\left\{a_{x}^{2}\right\}+E^{*}\left\{a_{x}a_{y}\right\}E\{a_{x}a_{y}|a_{y}{|}^{2}\}$	$E^{*}\left\{a_{x}^{2}\right\}E\{a_{x}^{2}|a_{y}{|}^{2}\}$	$R_{s}^{2}\Delta_{f}\delta_{m+n^{\prime }}\delta_{k+n-k^{\prime }+m^{\prime }}$ $-R_{s}\Delta_{f}^{2}\delta_{k-m+n-k^{\prime }+m^{\prime }-n^{\prime }}$
10	$E\{|a_{x}{|}^{4}\}E\{|a_{x}{|}^{2}\}+E\left\{a_{x}a_{y}^{*}\right\}E\{a_{x}^{*}a_{y}|a_{y}{|}^{2}\}$	$E\left\{a_{x}a_{y}^{*}\right\}E\{a_{x}^{*}a_{y}|a_{x}{|}^{2}\}$	$R_{s}^{2}\Delta_{f}\delta_{n-k^{\prime }}\delta_{k-m+m^{\prime }-n^{\prime }}$ $-R_{s}\Delta_{f}^{2}\delta_{k-m+n-k^{\prime }+m^{\prime }-n^{\prime }}$
11	$E^{*}\{a_{x}^{2}|a_{x}{|}^{2}\}E\left\{a_{x}^{2}\right\}+E\left\{a_{x}a_{y}\right\}E^{*}\{a_{x}a_{y}|a_{y}{|}^{2}\}$	$E\left\{a_{x}a_{y}\right\}E^{*}\{a_{x}a_{y}|a_{x}{|}^{2}\}$	$R_{s}^{2}\Delta_{f}\delta_{n+m^{\prime }}\delta_{k-m-k^{\prime }-n^{\prime }}$ $-R_{s}\Delta_{f}^{2}\delta_{k-m+n-k^{\prime }+m^{\prime }-n^{\prime }}$
12	$E\{|a_{x}{|}^{4}\}E\{|a_{x}{|}^{2}\}+E\{|a_{x}{|}^{2}\}E\{|a_{y}{|}^{4}\}$	$E\{|a_{x}{|}^{2}\}E\{|a_{x}{|}^{2}|a_{y}{|}^{2}\}$	$R_{s}^{2}\Delta_{f}\delta_{n-n^{\prime }}\delta_{k-m-k^{\prime }+m^{\prime }}$ $-R_{s}\Delta_{f}^{2}\delta_{k-m+n-k^{\prime }+m^{\prime }-n^{\prime }}$
13	$E\{|a_{x}{|}^{4}\}E\{|a_{x}{|}^{2}\}+E\{|a_{x}{|}^{2}|a_{y}{|}^{2}\}E\{|a_{y}{|}^{2}\}$	$E\{|a_{x}{|}^{4}\}E\{|a_{y}{|}^{2}\}$	$R_{s}^{2}\Delta_{f}\delta_{k^{\prime }-m^{\prime }}\delta_{k-m+n-n^{\prime }}$ $-R_{s}\Delta_{f}^{2}\delta_{k-m+n-k^{\prime }+m^{\prime }-n^{\prime }}$
14	$E\{a_{x}^{2}|a_{x}{|}^{2}\}E^{*}\left\{a_{x}^{2}\right\}+E^{*}\left\{a_{x}a_{y}\right\}E\{a_{x}a_{y}|a_{y}{|}^{2}\}$	$E^{*}\left\{a_{x}a_{y}\right\}E\{a_{x}a_{y}|a_{x}{|}^{2}\}$	$R_{s}^{2}\Delta_{f}\delta_{k^{\prime }+n^{\prime }}\delta_{k-m+n+m^{\prime }}$ $-R_{s}\Delta_{f}^{2}\delta_{k-m+n-k^{\prime }+m^{\prime }-n^{\prime }}$
15	$E\{|a_{x}{|}^{4}\}E\{|a_{x}{|}^{2}\}+E\left\{a_{x}^{*}a_{y}\right\}E\{a_{x}a_{y}^{*}|a_{y}{|}^{2}\}$	$E\left\{a_{x}^{*}a_{y}\right\}E\{a_{x}a_{y}^{*}{\left|a_{x}\right|}^{2}$	$R_{s}^{2}\Delta_{f}\delta_{m^{\prime }-n^{\prime }}\delta_{k-m+n-k^{\prime }}$ $-R_{s}\Delta_{f}^{2}\delta_{k-m+n-k^{\prime }+m^{\prime }-n^{\prime }}$

**Table 6:** Correlation coefficients in (40): the values of $a_{1},a_{1}^{\prime },b_{1},b_{1}^{\prime },\ldots d_{1}^{\prime }$ are given in Table A5.

Name	Value	Name	Value
$\Phi_{1}$	a1+2Re{a1′}	$\Lambda_{1}$	$c_{1}+c_{1}^{\prime }$
$\Phi_{2}$	a2+2Re{a2′}	$\Lambda_{2}$	$c_{6}^{\prime }$
$\Phi_{3}$	a3+2Re{a3′}	$\Lambda_{3}$	c2+2Re{c2′}
$\Psi_{1}$	b1+2Re{b1′}	$\Lambda_{4}$	$c_{3}+c_{3}^{\prime }$
$\Psi_{2}$	$b_{2}+b_{2}^{\prime }$	$\Lambda_{5}$	$c_{5}^{\prime }$
$\Psi_{3}$	$b_{3}^{\prime }$	$\Lambda_{6}$	c4+2Re{c4′}
$\Psi_{4}$	b4+2Re{b4′}	$\Xi_{1}$	d1+2Re{d1′}

**Table 7:** Delta products $D^{\left(l\right)}$ in the $Q_{l}$ terms, l=1,2,…,11, in (41) with their corresponding D set in Table 5.

*l*	$D^{\left(l\right)}$	Set D
1	$\delta_{k-k^{\prime }}\delta_{m-m^{\prime }}\delta_{n-n^{\prime }}$	$D_{1}$
2	$\delta_{k-k^{\prime }}\delta_{m+n^{\prime }}\delta_{n+m^{\prime }}$	$D_{2}$
3	$\delta_{k+n}\delta_{m-m^{\prime }}\delta_{k^{\prime }+n^{\prime }}$	$D_{3}$
4	$\delta_{k-m-k^{\prime }}\delta_{n+m^{\prime }-n^{\prime }}$	$D_{4}$
5	$\delta_{k-m+m^{\prime }}\delta_{n-k^{\prime }-n^{\prime }}$	$D_{5}$
6	$\delta_{k+n+m^{\prime }}\delta_{m+k^{\prime }+n^{\prime }}$	$D_{7}$
7	$\delta_{k+n}\delta_{m+k^{\prime }-m^{\prime }+n^{\prime }}$	$D_{8}$
8	$\delta_{k-k^{\prime }}\delta_{m-n-m^{\prime }+n^{\prime }}$	$D_{9}$
9	$\delta_{k+m^{\prime }}\delta_{m-n+k^{\prime }+n^{\prime }}$	$D_{10}$
10	$\delta_{m-m^{\prime }}\delta_{k+n-k^{\prime }-n^{\prime }}$	$D_{11}$
11	$\delta_{k-m+n-k^{\prime }+m^{\prime }-n^{\prime }}$	$D_{14}$

## Appendix A. Proof of Theorem 1

The Manakov Equation (8) can be written in the frequency domain as

(A1) $$\begin{array}{cc} \frac{\partial E(f,z)}{\partial z} & =-\frac{\alpha }{2}E\left(f,z\right)+j4\pi^{2}f^{2}\frac{\beta_{2}}{2}E\left(f,z\right)+j\gamma \frac{8}{9}F\{|\tilde{E}\left(t,z\right){|}^{2}\tilde{E}\left(t,z\right)\}\\ \; & =\left(-\frac{\alpha }{2}+j4\pi^{2}f^{2}\frac{\beta_{2}}{2}\right)E\left(f,z\right)+j\gamma \frac{8}{9}F\{|\tilde{E}\left(t,z\right){|}^{2}\}*E\left(f,z\right), \end{array}$$

where ∗ denotes a modified convolution operator between a scalar function and a vector function (For a scalar function α and a vector function $B={\left[B_{x},B_{y}\right]}^{T}$, the operator α∗B is defined here as $\alpha *B≜{\left[\alpha *B_{x},\alpha *B_{y}\right]}^{T}$). Expanding the nonlinear term in (A1), we have

$$F\{|\tilde{E}\left(t,z\right){|}^{2}\}*E\left(f,z\right)=\left(F\left\{{\tilde{E}}_{x}\left(t,z\right){\tilde{E}}_{x}^{*}\left(t,z\right)\right\}+F\left\{{\tilde{E}}_{y}\left(t,z\right){\tilde{E}}_{y}^{*}\left(t,z\right)\right\}\right)*{\left[E_{x}\left(f,z\right),E_{y}\left(f,z\right)\right]}^{T},$$

which, for instance, for the *x* component, becomes

(A2) $$E_{x}\left(f,z\right)*E_{x}^{*}\left(-f,z\right)*E_{x}\left(f,z\right)+E_{y}\left(f,z\right)*E_{y}^{*}\left(-f,z\right)*E_{x}\left(f,z\right).$$

Expanding the first term in (A2), we obtain

(A3) $$E_{x}\left(f,z\right)*E_{x}^{*}\left(-f,z\right)*E_{x}\left(f,z\right)=\int_{-\infty }^{\infty }\int_{-\infty }^{\infty }E_{x}\left(f_{1},z\right)E_{x}^{*}\left(f_{1}-f_{2},z\right)E_{x}\left(f-f_{2},z\right)df_{1}df_{2},$$

which by substitution $f_{1}-f_{2}={\tilde{f}}_{2}$ becomes (for notation’s simplicity, the integration variable ${\tilde{f}}_{2}$ is relabelled as $f_{2}$)

(A4) $$E_{x}\left(f,z\right)*E_{x}^{*}\left(-f,z\right)*E_{x}\left(f,z\right)=-\int_{-\infty }^{\infty }\int_{-\infty }^{\infty }E_{x}\left(f_{1},z\right)E_{x}^{*}\left(f_{2},z\right)E_{x}\left(f-f_{1}+f_{2},z\right)df_{1}df_{2}.$$

Similar to the steps in (A3) and (A4), the second term in (A2) can be found as

$$E_{y}\left(f,z\right)*E_{y}^{*}\left(-f,z\right)*E_{x}\left(f,z\right)=-\int_{-\infty }^{\infty }\int_{-\infty }^{\infty }E_{y}\left(f_{1},z\right)E_{y}^{*}\left(f_{2},z\right)E_{x}\left(f-f_{1}+f_{2},z\right)df_{1}df_{2}.$$

The *x* component in (A1) can be then rewritten as

(A5) $$\begin{array}{cc} \frac{\partial E_{x}\left(f,z\right)}{\partial z} & =\left(-\frac{\alpha }{2}+j2\pi^{2}f^{2}\beta_{2}\right)E_{x}\left(f,z\right)-j\frac{8}{9}\gamma \int_{-\infty }^{\infty }\int_{-\infty }^{\infty }\left(E_{x}\left(f_{1},z\right)E_{x}^{*}\left(f_{2},z\right)E_{x}\left(f-f_{1}+f_{2},z\right)\right.\\ \; & \left.+E_{y}\left(f_{1},z\right)E_{y}^{*}\left(f_{2},z\right)E_{x}\left(f-f_{1}+f_{2},z\right)\right)df_{1}df_{2}. \end{array}$$

Following the first-order RP approach to finding the solution to the Manakov equation [13], we replace the *x* component of the first-order expansion in (9) into (A5) and equate terms with the same power of γ. After some algebra and after substituting the $A_{n}$ terms with the corresponding $E_{n}$ using (10), we find the following set of differential equations

(A6) $$\begin{array}{cc} \frac{\partial E_{0,x}\left(f,z\right)}{\partial z} & =\left(-\frac{\alpha }{2}+j2\pi^{2}f^{2}\beta_{2}\right)E_{0,x}\left(f,z\right), \end{array}$$

(A7) $$\begin{array}{c} \begin{array}{cc} \frac{\partial E_{1,x}\left(f,z\right)}{\partial z} & =-j\frac{8}{9}\gamma \int_{-\infty }^{\infty }\int_{-\infty }^{\infty }\left(E_{0,x}\left(f_{1},z\right)E_{0,x}^{*}\left(f_{2},z\right)E_{0,x}\left(f-f_{1}+f_{2},z\right)\right.\\ \; & \left.+E_{0,y}\left(f_{1},z\right)E_{0,y}^{*}\left(f_{2},z\right)E_{0,x}\left(f-f_{1}+f_{2},z\right)\right)df_{1}df_{2}. \end{array} \end{array}$$

The zeroth-order term for a single fibre span of length *z* is given by

(A8) $$E_{0,x}\left(f,z\right)=E\left(f,0\right)e^{(-\alpha /2+j2\pi^{2}\beta_{2}f^{2})z}.$$

On the other hand, the first-order term (for the *x* component) $E_{1,x}\left(f,z\right)$, with initial conditions given by the transmitted signal E(f,0), can be found solving the following differential equation

(A9) $$\begin{array}{cc} \frac{\partial E_{1,x}\left(f,z\right)}{\partial z}= & \left(-\frac{\alpha }{2}+j2\pi^{2}\beta_{2}f^{2}\right)E_{1,x}\left(f,z\right)-j\frac{8}{9}\gamma \int_{-\infty }^{\infty }\int_{-\infty }^{\infty }\left(E_{0,x}\left(f_{1},z\right)E_{0,x}^{*}\left(f_{2},z\right)E_{0,x}\left(f-f_{1}+f_{2},z\right)\right.\\ \; & \left.+E_{0,y}\left(f_{1},z\right)E_{0,y}^{*}\left(f_{2},z\right)E_{0,y}\left(f-f_{1}+f_{2},z\right)\right)df_{1}df_{2}. \end{array}$$

The solution to (A9) with initial condition $E_{1,x}\left(f,0\right)=0$ is given by

(A10a) $$\begin{array}{c} \begin{array}{cc} E_{1,x}\left(f,z\right) & =-j\frac{8}{9}\gamma e^{\left(-\alpha z+j2\beta_{2}\pi^{2}f^{2}z\right)}\int_{0}^{z}e^{\left(\frac{\alpha }{2}-j2\beta_{2}\pi^{2}f^{2}\right)z^{\prime }}\int_{-\infty }^{\infty }\int_{-\infty }^{\infty }\left(E_{0,x}\left(f_{1},z^{\prime }\right)E_{0,x}^{*}\left(f_{2},z^{\prime }\right)E_{0,x}\left(f-f_{1}+f_{2},z^{\prime }\right)\right.\\ \; & \left.+E_{0,y}\left(f_{1},z^{\prime }\right)E_{0,y}^{*}\left(f_{2},z^{\prime }\right)E_{0,x}\left(f-f_{1}+f_{2},z^{\prime }\right)\right)df_{1}df_{2}dz^{\prime } \end{array} \end{array}$$

(A10b) $$\begin{array}{c} \begin{array}{cc} \; & =-j\frac{8}{9}\gamma e^{\left(-\alpha z+j2\beta_{2}\pi^{2}f^{2}z\right)}\int_{0}^{z}e^{\left(\frac{\alpha }{2}-j2\beta_{2}\pi^{2}f^{2}\right)z^{\prime }}\int_{-\infty }^{\infty }\int_{-\infty }^{\infty }\left(E_{x}\left(f_{1},0\right)E_{x}^{*}\left(f_{2},0\right)E_{x}\left(f-f_{1}+f_{2},0\right)\right.\\ \; & \left.+E_{y}\left(f_{1},0\right)E_{y}^{*}\left(f_{2},0\right)E_{x}\left(f-f_{1}+f_{2},0\right)\right)e^{-\frac{3}{2}\alpha z^{\prime }}e^{j4\pi^{2}\frac{\beta_{2}}{2}\left(f_{1}^{2}-f_{2}^{2}+{\left(f-f_{1}+f_{2}\right)}^{2}\right)z^{\prime }}df_{1}df_{2}dz^{\prime }, \end{array} \end{array}$$

where (A8) was used in the step from (A10a) to (A10b).

The power profile assumed in Section 3.1 for the multi-span optical link exponentially decays with a lumped amplification at the end of each span, which brings the power back to the transmitted level. This behavior leads to a discontinuity in the function α(z) across the interface where an amplifier is located. For such a power profile, we can solve the differential Equations (A6) and (A7) by exploiting the continuity of their coefficients within each span and by imposing the initial conditions at the input of each new fibre span $E_{0,x}\left(f,lL_{s}^{+}\right)=e^{\alpha L_{s}/2}E_{0,x}\left(f,lL_{s}^{-}\right)$ and $E_{1,x}\left(f,lL_{s}^{+}\right)=e^{\alpha L_{s}/2}E_{1,x}\left(f,lL_{s}^{-}\right)$, for $l=1,2,\ldots ,N_{s}$. Here, $z=lL_{s}^{-}$ and $z=lL_{s}^{+}$ indicate the sections at the input and at the output of the *l*th amplifier, respectively. Thus, we obtain that the zeroth and first-order term after $N_{s}$ fibre spans are given by

(A11) $$\begin{array}{c} \begin{array}{cc} E_{0,x}\left(f,N_{s},L_{s}\right) & =E\left(f,0\right)e^{j2\pi^{2}\beta_{2}f^{2}N_{s}L_{s}}, \end{array} \end{array}$$

(A12) $$\begin{array}{c} \begin{array}{cc} E_{1,x}\left(f,N_{s},L_{s}\right) & =-j\frac{8}{9}\gamma e^{j2\beta_{2}\pi^{2}f^{2}N_{s}L_{s}}\sum_{l=1}^{N_{s}}\int_{\left(l-1\right)L_{s}}^{lL_{s}}e^{\left(\frac{\alpha }{2}-j2\beta_{2}\pi^{2}f^{2}\right)z^{\prime }}\\ \; & \cdot \int_{-\infty }^{\infty }\int_{-\infty }^{\infty }\left(E_{0,x}\left(f_{1},z^{\prime }\right)E_{0,x}^{*}\left(f_{2},z^{\prime }\right)E_{0,x}\left(f-f_{1}+f_{2},z^{\prime }\right)\right.\\ \; & \left.+E_{0,y}\left(f_{1},z^{\prime }\right)E_{0,y}^{*}\left(f_{2},z^{\prime }\right)E_{0,x}\left(f-f_{1}+f_{2},z^{\prime }\right)\right)df_{1}df_{2}dz^{\prime }. \end{array} \end{array}$$

Using (A11) in (A12) and swapping the integral in $z^{\prime }$ with the double integral in $df_{1}df_{2}$, we obtain

$$\begin{array}{cc} E_{1,x}\left(f,N_{s},L_{s}\right) & =-j\frac{8}{9}\gamma e^{j2\beta_{2}\pi^{2}f^{2}N_{s}L_{s}}\int_{-\infty }^{\infty }\int_{-\infty }^{\infty }\left(E_{x}\left(f_{1},0\right)E_{x}^{*}\left(f_{2},0\right)E_{x}\left(f-f_{1}+f_{2},0\right)\right.\\ \; & \left.+E_{y}\left(f_{1},0\right)E_{y}^{*}\left(f_{2},0\right)E_{x}\left(f-f_{1}+f_{2},0\right)\right)\\ \; & \cdot \sum_{l=1}^{N_{s}}\int_{\left(l-1\right)L_{s}}^{lL_{s}}e^{\left[-\alpha +j\beta_{2}\left(f-f_{1}\right)\left(f_{2}-f_{1}\right)\right]z^{\prime }}dz^{\prime }df_{1}df_{2}\\ =-j\frac{8}{9}\gamma e^{j2\beta_{2}\pi^{2}f^{2}N_{s}L_{s}}\int_{-\infty }^{\infty } & \int_{-\infty }^{\infty }\left(E_{x}\left(f_{1},0\right)E_{x}^{*}\left(f_{2},0\right)E_{x}\left(f-f_{1}+f_{2},0\right)\right.\\ \; & \left.+E_{y}\left(f_{1},0\right)E_{y}^{*}\left(f_{2},0\right)E_{x}\left(f-f_{1}+f_{2},0\right)\right)\\ \; & \cdot \frac{1-e^{-\alpha z}e^{j\beta_{2}\left(f-f_{1}\right)\left(f_{2}-f_{1}\right)z}}{\alpha -j\beta_{2}\left(f-f_{1}\right)\left(f_{2}-f_{1}\right)}\sum_{l=1}^{N_{s}}e^{-j4\pi^{2}\beta_{2}\left(l-1\right)\left(f-f_{1}\right)\left(f_{2}-f_{1}\right)L_{s}}df_{1}df_{2}. \end{array}$$

The *y*-component of the zeroth-order term $E_{0,y}\left(f,z\right)$ and first-order term $E_{1,y}\left(f,z\right)$ can be found using the transformation x→y, y→x in (A11) and (A12), respectively. Finally, bringing together the *x* and *y* components, we have

$$\begin{array}{c} \begin{array}{cc} E_{0}\left(f,N_{s},L_{s}\right)= & E_{0}\left(f,0\right)e^{j2\pi^{2}f^{2}\beta_{2}N_{s}L_{s}}, \end{array}\\ \begin{array}{cc} E_{1}\left(f,N_{s},L_{s}\right)=-j\frac{8}{9}\gamma e^{j2\beta_{2}\pi^{2}f^{2}N_{s}L_{s}}\int_{-\infty }^{\infty }\int_{-\infty }^{\infty } & E^{T}\left(f_{1},0\right)E^{*}\left(f_{2},0\right)E\left(f-f_{1}+f_{2},0\right)\eta \left(f_{1},f_{2},f,z\right)df_{1}df_{2}, \end{array} \end{array}$$

where $\eta (f,f_{1},f_{2},z)$ is defined in (12), which proves the theorem.

## Appendix B. Proof of Proposition 1

Applying the variable transformation $\tilde{k}=k^{\prime },\tilde{m}=m^{\prime },\tilde{n}=n^{\prime },{\tilde{k}}^{\prime }=k,{\tilde{m}}^{\prime }=m,and{\tilde{n}}^{\prime }=n$ to the left-hand side of (23), we obtain

(A13a) $$\begin{array}{cc} \; & \sum_{\begin{array}{c} \left(k,m,n\right)\in S_{i}\\ \left(k^{\prime },m^{\prime },n^{\prime }\right)\in S_{i} \end{array}}P_{k,m,n,k^{\prime },m^{\prime },n^{\prime }}\nu_{x,k}\nu_{x,m}^{*}\nu_{x,n}\nu_{y,k^{\prime }}^{*}\nu_{y,m^{\prime }}\nu_{x,n^{\prime }}^{*}\eta_{k,n,m}\eta_{k^{\prime },n^{\prime },m^{\prime }}^{*} \end{array}$$

(A13b) $$\begin{array}{cc} \; & =\sum_{\begin{array}{c} \left({\tilde{k}}^{\prime },{\tilde{m}}^{\prime },{\tilde{n}}^{\prime }\right)\in S_{i}\\ \left(\tilde{k},\tilde{m},\tilde{n}\right)\in S_{i} \end{array}}P_{{\tilde{k}}^{\prime },{\tilde{m}}^{\prime },{\tilde{n}}^{\prime },\tilde{k},\tilde{m},\tilde{n}}\nu_{x,{\tilde{k}}^{\prime }}\nu_{x,{\tilde{m}}^{\prime }}^{*}\nu_{x,{\tilde{n}}^{\prime }}\nu_{y,\tilde{k}}^{*}\nu_{y,\tilde{m}}\nu_{x,\tilde{n}}^{*}\eta_{{\tilde{k}}^{\prime },{\tilde{m}}^{\prime },{\tilde{n}}^{\prime }}\eta_{\tilde{k},\tilde{m},\tilde{n}}^{*} \end{array}$$

(A13c) $$\begin{array}{cc} \; & =\sum_{\begin{array}{c} \left(\tilde{k},\tilde{m},\tilde{n}\right)\in S_{i}\\ \left({\tilde{k}}^{\prime },{\tilde{m}}^{\prime },{\tilde{n}}^{\prime }\right)\in S_{i} \end{array}}P_{\tilde{k},\tilde{m},\tilde{n},{\tilde{k}}^{\prime },{\tilde{m}}^{\prime },{\tilde{n}}^{\prime }}^{*}{\left(\nu_{y,\tilde{k}}^{*}\nu_{y,\tilde{m}}\nu_{x,\tilde{n}}^{*}\nu_{x,{\tilde{k}}^{\prime }}\nu_{x,{\tilde{m}}^{\prime }}^{*}\nu_{x,{\tilde{n}}^{\prime }}\eta_{\tilde{k},\tilde{m},\tilde{n}}\eta_{{\tilde{k}}^{\prime },{\tilde{m}}^{\prime },{\tilde{n}}^{\prime }}^{*}\right)}^{*} \end{array}$$

(A13d) $$\begin{array}{cc} \; & =(\sum_{\begin{array}{c} \left(\tilde{k},\tilde{m},\tilde{n}\right)\in S_{i}\\ \left({\tilde{k}}^{\prime },{\tilde{m}}^{\prime },{\tilde{n}}^{\prime }\right)\in S_{i} \end{array}}P_{\tilde{k},\tilde{m},\tilde{n},{\tilde{k}}^{\prime },{\tilde{m}}^{\prime },{\tilde{n}}^{\prime }}\nu_{y,\tilde{k}}\nu_{y,\tilde{m}}^{*}\nu_{x,\tilde{n}}\nu_{x,{\tilde{k}}^{\prime }}^{*}\nu_{x,{\tilde{m}}^{\prime }}\nu_{x,{\tilde{n}}^{\prime }}^{*}\eta_{\tilde{k},\tilde{m},\tilde{n}}^{*}\eta_{{\tilde{k}}^{\prime },{\tilde{m}}^{\prime },{\tilde{n}}^{\prime }}{)}^{*}, \end{array}$$

where in the step between (A13b) and (A13c), we have used the property

(A14) $$P_{{\tilde{k}}^{\prime },{\tilde{m}}^{\prime },{\tilde{n}}^{\prime },\tilde{k},\tilde{m},\tilde{n}}=P_{\tilde{k},\tilde{m},\tilde{n},{\tilde{k}}^{\prime },{\tilde{m}}^{\prime },{\tilde{n}}^{\prime }}^{*},$$

which can be easily verified based on definition (22). Using the relabelling $\tilde{k}\to k,\tilde{m}\to m,\tilde{n}\to n,{\tilde{k}}^{\prime }\to k^{\prime },{\tilde{m}}^{\prime }\to m^{\prime },{\tilde{n}}^{\prime }\to n^{\prime }$ for (A13d), the proposition is proven.

## Appendix C. Partition Tables for Sets $L_{1}$, $L_{2}$, and $L_{3}$

**Table A1:** List of all subsets in $L_{1}$: for each subset, the index subgroups identify the corresponding pairs of indices assuming the same value.

	Index Subgroup	1	2	3
Subset Label				
$C_{1}^{\left(1\right)}$		$i_{1},i_{2}$	$i_{3},i_{4}$	$i_{5},i_{6}$
$C_{1}^{\left(2\right)}$		$i_{1},i_{2}$	$i_{3},i_{5}$	$i_{4},i_{6}$
$C_{1}^{\left(3\right)}$		$i_{1},i_{2}$	$i_{3},i_{6}$	$i_{4},i_{5}$
$C_{1}^{\left(4\right)}$		$i_{1},i_{3}$	$i_{2},i_{4}$	$i_{5},i_{6}$
$C_{1}^{\left(5\right)}$		$i_{1},i_{3}$	$i_{2},i_{5}$	$i_{4},i_{6}$
$C_{1}^{\left(6\right)}$		$i_{1},i_{3}$	$i_{2},i_{6}$	$i_{4},i_{5}$
$C_{1}^{\left(7\right)}$		$i_{1},i_{4}$	$i_{2},i_{3}$	$i_{5},i_{6}$
$C_{1}^{\left(8\right)}$		$i_{1},i_{4}$	$i_{2},i_{5}$	$i_{3},i_{6}$
$C_{1}^{\left(9\right)}$		$i_{1},i_{4}$	$i_{2},i_{6}$	$i_{3},i_{5}$
$C_{1}^{\left(10\right)}$		$i_{1},i_{5}$	$i_{2},i_{3}$	$i_{4},i_{6}$
$C_{1}^{\left(11\right)}$		$i_{1},i_{5}$	$i_{2},i_{4}$	$i_{3},i_{6}$
$C_{1}^{\left(12\right)}$		$i_{1},i_{5}$	$i_{2},i_{6}$	$i_{3},i_{4}$
$C_{1}^{\left(13\right)}$		$i_{1},i_{6}$	$i_{2},i_{3}$	$i_{4},i_{5}$
$C_{1}^{\left(14\right)}$		$i_{1},i_{6}$	$i_{2},i_{4}$	$i_{3},i_{5}$
$C_{1}^{\left(15\right)}$		$i_{1},i_{6}$	$i_{2},i_{5}$	$i_{3},i_{4}$

**Table A2:** List of all subsets in $L_{2}$: for each subset, the index subgroups identify the corresponding triplets of indices assuming the same value.

	Subgroup Index	1	2
Subset Label			
$C_{2}^{\left(1\right)}$		$i_{1},i_{2},i_{3}$	$i_{4},i_{5},i_{6}$
$C_{2}^{\left(2\right)}$		$i_{1},i_{2},i_{4}$	$i_{3},i_{5},i_{6}$
$C_{2}^{\left(3\right)}$		$i_{1},i_{2},i_{5}$	$i_{3},i_{4},i_{6}$
$C_{2}^{\left(4\right)}$		$i_{1},i_{2},i_{6}$	$i_{3},i_{4},i_{5}$
$C_{2}^{\left(5\right)}$		$i_{1},i_{3},i_{4}$	$i_{2},i_{5},i_{6}$
$C_{2}^{\left(6\right)}$		$i_{1},i_{3},i_{5}$	$i_{2},i_{4},i_{6}$
$C_{2}^{\left(7\right)}$		$i_{1},i_{3},i_{6}$	$i_{2},i_{4},i_{5}$
$C_{2}^{\left(8\right)}$		$i_{1},i_{4},i_{5}$	$i_{2},i_{3},i_{6}$
$C_{2}^{\left(9\right)}$		$i_{1},i_{4},i_{6}$	$i_{2},i_{3},i_{5}$
$C_{2}^{\left(10\right)}$		$i_{1},i_{5},i_{6}$	$i_{2},i_{3},i_{4}$

**Table A3:** List of all subsets in $L_{3}$: for each subset, the index subgroups identify the corresponding pair and quadruple of indices assuming the same value. The set $C_{3}^{\left(1\right)}$ corresponds to the case discussed in Example 2.

	Subgroup Index	1	2
Subset Label			
$C_{3}^{\left(1\right)}$		$i_{1},i_{2}$	$i_{3},i_{4},i_{5},i_{6}$
$C_{3}^{\left(2\right)}$		$i_{1},i_{3}$	$i_{2},i_{4},i_{5},i_{6}$
$C_{3}^{\left(3\right)}$		$i_{1},i_{4}$	$i_{2},i_{3},i_{5},i_{6}$
$C_{3}^{\left(4\right)}$		$i_{1},i_{5}$	$i_{2},i_{3},i_{4},i_{6}$
$C_{3}^{\left(5\right)}$		$i_{1},i_{6}$	$i_{2},i_{3},i_{4},i_{5}$
$C_{3}^{\left(6\right)}$		$i_{2},i_{3}$	$i_{1},i_{4},i_{5},i_{6}$
$C_{3}^{\left(7\right)}$		$i_{2},i_{4}$	$i_{1},i_{3},i_{5},i_{6}$
$C_{3}^{\left(8\right)}$		$i_{2},i_{5}$	$i_{1},i_{3},i_{4},i_{6}$
$C_{3}^{\left(9\right)}$		$i_{2},i_{6}$	$i_{1},i_{3},i_{4},i_{5}$
$C_{3}^{\left(10\right)}$		$i_{3},i_{4}$	$i_{1},i_{2},i_{5},i_{6}$
$C_{3}^{\left(11\right)}$		$i_{3},i_{5}$	$i_{1},i_{2},i_{4},i_{6}$
$C_{3}^{\left(12\right)}$		$i_{3},i_{6}$	$i_{1},i_{2},i_{4},i_{5}$
$C_{3}^{\left(13\right)}$		$i_{4},i_{5}$	$i_{1},i_{2},i_{3},i_{6}$
$C_{3}^{\left(14\right)}$		$i_{4},i_{6}$	$i_{1},i_{2},i_{3},i_{5}$
$C_{3}^{\left(15\right)}$		$i_{5},i_{6}$	$i_{1},i_{2},i_{3},i_{4}$

## Appendix D. $M_{g}^{\left(h\right)}$ and $N_{g}^{\left(h\right)}$ Contributions

**Table A4:** List of $M_{g}^{\left(h\right)}$ and $N_{g}^{\left(h\right)}$ contributions computed in Section 5 without bias terms.

*g*	*h*	Corr. Terms in $M_{g}^{\left(h\right)}$	Corr. Terms in $N_{g}^{\left(h\right)}$	Delta Products
1	1	$E^{3}\left\{\right|a_{x}{|}^{2}\}+|E\left\{a_{x}a_{y}^{*}\right\}{|}^{2}E\{|a_{y}{|}^{2}\}$	$E\{|a_{x}{|}^{2}\}|E\left\{a_{x}a_{y}^{*}\right\}{|}^{2}$	$-R_{s}^{2}\Delta_{f}\delta_{n-k^{\prime }}\delta_{k-m+m^{\prime }-n^{\prime }}$ $+2R_{s}\Delta_{f}^{2}\delta_{k-m+n-k^{\prime }+m^{\prime }-n^{\prime }}$
	2	$E\{|a_{x}{|}^{2}\}|E\left\{a_{x}^{2}\right\}{|}^{2}+|E\left\{a_{x}a_{y}\right\}{|}^{2}E\{|a_{y}{|}^{2}\}$	$E\{|a_{x}{|}^{2}\}|E\left\{a_{x}a_{y}\right\}{|}^{2}$	$-R_{s}^{2}\Delta_{f}(\delta_{k^{\prime }+n^{\prime }}\delta_{k-m+n+m^{\prime }}$ $+\delta_{n+m^{\prime }}\delta_{k-m-k^{\prime }-n^{\prime }})$ $+2R_{s}\Delta_{f}^{2}\delta_{k-m+n-k^{\prime }+m^{\prime }-n^{\prime }}$
	3	$E^{3}\left\{\right|a_{x}{|}^{2}\}+E\{|a_{x}{|}^{2}\}E^{2}\left\{\right|a_{y}{|}^{2}\}$	$E^{2}\left\{\right|a_{x}{|}^{2}\}E\{|a_{y}{|}^{2}\}$	$-R_{s}^{2}\Delta_{f}\delta_{n-n^{\prime }}\delta_{k-m-k^{\prime }+m^{\prime }}$ $+2R_{s}\Delta_{f}^{2}\delta_{k-m+n-k^{\prime }+m^{\prime }-n^{\prime }}$
	4	$E\{|a_{x}{|}^{2}\}|E\left\{a_{x}^{2}\right\}{|}^{2}+E\left\{a_{x}a_{y}\right\}E\left\{a_{x}^{*}a_{y}\right\}E^{*}\left\{a_{y}^{2}\right\}$	$E\left\{a_{x}^{2}\right\}E^{*}\left\{a_{x}a_{y}\right\}E\left\{a_{x}^{*}a_{y}\right\}$	$-R_{s}^{2}\Delta_{f}(\delta_{m+k^{\prime }}\delta_{k+n+m^{\prime }-n^{\prime }}$ $+\delta_{k+n}\delta_{m+k^{\prime }-m^{\prime }+n^{\prime }})$ $+2R_{s}\Delta_{f}^{2}\delta_{k-m+n-k^{\prime }+m^{\prime }-n^{\prime }}$
	5	$E\{|a_{x}{|}^{2}\}|E\left\{a_{x}^{2}\right\}{|}^{2}+|E\left\{a_{x}a_{y}\right\}{|}^{2}E\{|a_{y}{|}^{2}\}$	$E\left\{a_{x}^{2}\right\}E^{*}\left\{a_{x}a_{y}\right\}E\left\{a_{x}^{*}a_{y}\right\}$	$R_{s}^{3}\delta_{k+n}\delta_{m-m^{\prime }}\delta_{k^{\prime }+n^{\prime }}$ $-R_{s}^{2}\Delta_{f}(\delta_{k^{\prime }+n^{\prime }}\delta_{k-m+n+m^{\prime }}$ $+\delta_{m-m^{\prime }}\delta_{k+n-k^{\prime }-n^{\prime }}$ $+\delta_{k+n}\delta_{m-m^{\prime }+k^{\prime }+n^{\prime }})$ $+2R_{s}\Delta_{f}^{2}\delta_{k-m+n-k^{\prime }+m^{\prime }-n^{\prime }}$
	6	$E\{|a_{x}{|}^{2}\}|E\left\{a_{x}^{2}\right\}{|}^{2}+|E\left\{a_{x}a_{y}\right\}{|}^{2}E\{|a_{y}{|}^{2}\}$	$|E\left\{a_{x}^{2}\right\}{|}^{2}E\{|a_{y}{|}^{2}\}$	$-R_{s}^{2}\Delta_{f}(\delta_{m+n^{\prime }}\delta_{k+n-k^{\prime }+m^{\prime }}$ $+\delta_{k+n}\delta_{m+k^{\prime }-m^{\prime }+n^{\prime }})$ $+2R_{s}\Delta_{f}^{2}\delta_{k-m+n-k^{\prime }+m^{\prime }-n^{\prime }}$
	7	$E^{3}\left\{\right|a_{x}{|}^{2}\}+|E\left\{a_{x}a_{y}^{*}\right\}{|}^{2}E\{|a_{y}{|}^{2}\}$	$E\{|a_{x}{|}^{2}\}|E\left\{a_{x}a_{y}^{*}\right\}{|}^{2}$	$-R_{s}^{2}\Delta_{f}\delta_{k-k^{\prime }}\delta_{m-n-m^{\prime }+n^{\prime }}$ $+2R_{s}\Delta_{f}^{2}\delta_{k-m+n-k^{\prime }+m^{\prime }-n^{\prime }}$
	8	$E^{3}\left\{\right|a_{x}{|}^{2}\}+E\{|a_{x}{|}^{2}\}E^{2}\left\{\right|a_{y}{|}^{2}\}$	$E\{|a_{x}{|}^{2}\}|E\left\{a_{x}a_{y}^{*}\right\}{|}^{2}$	$R_{s}^{3}\delta_{k-k^{\prime }}\delta_{m-m^{\prime }}\delta_{n-n^{\prime }}$ $-R_{s}^{2}\Delta_{f}(\delta_{n-n^{\prime }}\delta_{k-m-k^{\prime }+m^{\prime }}$ $+\delta_{m-m^{\prime }}\delta_{k+n-k^{\prime }-n^{\prime }}$ $+\delta_{k-k^{\prime }}\delta_{m-n-m^{\prime }+n^{\prime }})$ $+2R_{s}\Delta_{f}^{2}\delta_{k-m+n-k^{\prime }+m^{\prime }-n^{\prime }}$
	9	$E\{|a_{x}{|}^{2}\}|E\left\{a_{x}^{2}\right\}{|}^{2}+|E\left\{a_{x}a_{y}\right\}{|}^{2}E\{|a_{y}{|}^{2}\}$	$E^{*}\left\{a_{x}^{2}\right\}E\left\{a_{x}a_{y}\right\}E\left\{a_{x}a_{y}^{*}\right\}$	$R_{s}^{3}\delta_{k-k^{\prime }}\delta_{m+n^{\prime }}\delta_{n+m^{\prime }}$ $-R_{s}^{2}\Delta_{f}(\delta_{n+m^{\prime }}\delta_{k-m-k^{\prime }-n^{\prime }}$ $+\delta_{m+n^{\prime }}\delta_{k+n-k^{\prime }+m^{\prime }}$ $+\delta_{k-k^{\prime }}\delta_{m-n-m^{\prime }+n^{\prime }})$ $+2R_{s}\Delta_{f}^{2}\delta_{k-m+n-k^{\prime }+m^{\prime }-n^{\prime }}$
	10	$E\{|a_{x}{|}^{2}\}|E\left\{a_{x}^{2}\right\}{|}^{2}+E^{*}\left\{a_{x}a_{y}\right\}E\left\{a_{x}a_{y}^{*}\right\}E\left\{a_{y}^{2}\right\}$	$E\{|a_{x}{|}^{2}\}|E\left\{a_{x}a_{y}\right\}{|}^{2}$	$-R_{s}^{2}\Delta_{f}(\delta_{k^{\prime }+n^{\prime }}\delta_{k-m+n+m^{\prime }}$ $+\delta_{k+m^{\prime }}\delta_{m-n+k^{\prime }+n^{\prime }})$ $+2R_{s}\Delta_{f}^{2}\delta_{k-m+n-k^{\prime }+m^{\prime }-n^{\prime }}$
	11	$E\{|a_{x}{|}^{2}\}|E\left\{a_{x}^{2}\right\}{|}^{2}+E\{|a_{x}{|}^{2}\}|E\left\{a_{y}^{2}\right\}{|}^{2}$	$E\{|a_{x}{|}^{2}\}|E\left\{a_{x}a_{y}\right\}{|}^{2}$	$R_{s}^{3}\delta_{k+m^{\prime }}\delta_{m+k^{\prime }}\delta_{n-n^{\prime }}$ $-R_{s}^{2}\Delta_{f}(\delta_{n-n^{\prime }}\delta_{k-m-k^{\prime }+m^{\prime }}$ $+\delta_{m+k^{\prime }}\delta_{k+n+m^{\prime }-n^{\prime }}$ $+\delta_{k+m^{\prime }}\delta_{m-n+k^{\prime }+n^{\prime }})$ $+2R_{s}\Delta_{f}^{2}\delta_{k-m+n-k^{\prime }+m^{\prime }-n^{\prime }}$
	12	$E\{|a_{x}{|}^{2}\}|E\left\{a_{x}^{2}\right\}{|}^{2}+E^{*}\left\{a_{x}a_{y}\right\}E\left\{a_{x}a_{y}^{*}\right\}E\left\{a_{y}^{2}\right\}$	$E^{*}\left\{a_{x}^{2}\right\}E\left\{a_{x}a_{y}\right\}E\left\{a_{x}a_{y}^{*}\right\}$	$R_{s}^{3}\delta_{k+m^{\prime }}\delta_{m+n^{\prime }}\delta_{n-k^{\prime }}$ $-R_{s}^{2}\Delta_{f}(\delta_{n-k^{\prime }}\delta_{k-m+m^{\prime }-n^{\prime }}$ $+\delta_{m+n^{\prime }}\delta_{k+n-k^{\prime }+m^{\prime }}$ $+\delta_{k+m^{\prime }}\delta_{m-n+k^{\prime }+n^{\prime }})$ $+2R_{s}\Delta_{f}^{2}\delta_{k-m+n-k^{\prime }+m^{\prime }-n^{\prime }}$
	13	$E^{3}\left\{\right|a_{x}{|}^{2}\}+|E\left\{a_{x}a_{y}^{*}\right\}{|}^{2}E\{|a_{y}{|}^{2}\}$	$E^{2}\left\{\right|a_{x}{|}^{2}\}E\{|a_{y}{|}^{2}\}$	$-R_{s}^{2}\Delta_{f}\delta_{k-n^{\prime }}\delta_{m-n+k^{\prime }-m^{\prime }}$ $+2R_{s}\Delta_{f}^{2}\delta_{k-m+n-k^{\prime }+m^{\prime }-n^{\prime }}$
	14	$E\{|a_{x}{|}^{2}\}|E\left\{a_{x}^{2}\right\}{|}^{2}+E\left\{a_{x}a_{y}\right\}E\left\{a_{x}^{*}a_{y}\right\}E^{*}\left\{a_{y}^{2}\right\}$	$E\{|a_{x}{|}^{2}\}|E\left\{a_{x}a_{y}\right\}{|}^{2}$	$R_{s}^{3}\delta_{k-n^{\prime }}\delta_{m+k^{\prime }}\delta_{n+m^{\prime }}$ $-R_{s}^{2}\Delta_{f}(\delta_{n+m^{\prime }}\delta_{k-m-k^{\prime }-n^{\prime }}$ $+\delta_{m+k^{\prime }}\delta_{k+n+m^{\prime }-n^{\prime }}$ $+\delta_{k-n^{\prime }}\delta_{m-n+k^{\prime }-m^{\prime }})$ $+2R_{s}\Delta_{f}^{2}\delta_{k-m+n-k^{\prime }+m^{\prime }-n^{\prime }}$
	15	$E^{3}\left\{\right|a_{x}{|}^{2}\}+|E\left\{a_{x}a_{y}^{*}\right\}{|}^{2}E\{|a_{y}{|}^{2}\}$	$E\{|a_{x}{|}^{2}\}|E\left\{a_{x}a_{y}^{*}\right\}{|}^{2}$	$R_{s}^{3}\delta_{k-n^{\prime }}\delta_{m-m^{\prime }}\delta_{n-k^{\prime }}$ $-R_{s}^{2}\Delta_{f}(\delta_{n-k^{\prime }}\delta_{k-m+m^{\prime }-n^{\prime }}$ $+\delta_{m-m^{\prime }}\delta_{k+n-k^{\prime }-n^{\prime }}$ $+\delta_{k-n^{\prime }}\delta_{m-n+k^{\prime }-m^{\prime }})$ $+2R_{s}\Delta_{f}^{2}\delta_{k-m+n-k^{\prime }+m^{\prime }-n^{\prime }}$
2	1	$|E\{a_{x}|a_{x}{|}^{2}{\}|}^{2}+|E\{a_{x}|a_{y}{|}^{2}{\}|}^{2}$	$E\{a_{x}|a_{x}{|}^{2}\}E\{a_{x}|a_{y}{|}^{2}\}$	$-R_{s}\Delta_{f}^{2}\delta_{k-m+n-k^{\prime }+m^{\prime }-n^{\prime }}$
	2	$|E\{a_{x}|a_{x}{|}^{2}{\}|}^{2}+E\{a_{y}^{*}|a_{y}{|}^{2}\}E\{|a_{x}{|}^{2}a_{y}\}$	$\left|E\{\right|a_{x}{|}^{2}a_{y}{\}|}^{2}$	$R_{s}^{2}\Delta_{f}\delta_{k-m-k^{\prime }}\delta_{n+m^{\prime }-n^{\prime }}$ $-R_{s}\Delta_{f}^{2}\delta_{k-m+n-k^{\prime }+m^{\prime }-n^{\prime }}$
	3	$|E\{a_{x}|a_{x}{|}^{2}{\}|}^{2}+E\{|a_{x}{|}^{2}a_{y}^{*}\}E\{a_{y}|a_{y}{|}^{2}\}$	$\left|E\{\right|a_{x}{|}^{2}a_{y}{\}|}^{2}$	$R_{s}^{2}\Delta_{f}\delta_{k-m+m^{\prime }}\delta_{n-k^{\prime }-n^{\prime }}$ $-R_{s}\Delta_{f}^{2}\delta_{k-m+n-k^{\prime }+m^{\prime }-n^{\prime }}$
	4	$|E\{a_{x}|a_{x}{|}^{2}{\}|}^{2}+|E\{a_{x}|a_{y}{|}^{2}{\}|}^{2}$	$E\{a_{x}^{*}|a_{x}{|}^{2}\}E\{a_{x}|a_{y}{|}^{2}\}$	$R_{s}^{2}\Delta_{f}\delta_{k-m-n^{\prime }}\delta_{n-k^{\prime }+m^{\prime }}$ $-R_{s}\Delta_{f}^{2}\delta_{k-m+n-k^{\prime }+m^{\prime }-n^{\prime }}$
	5	$|E\{a_{x}|a_{x}{|}^{2}{\}|}^{2}+|E\{a_{x}|a_{y}{|}^{2}{\}|}^{2}$	$|E\left\{a_{x}^{2}a_{y}^{*}\right\}{|}^{2}$	$R_{s}^{2}\Delta_{f}\delta_{k+n-k^{\prime }}\delta_{m-m^{\prime }+n^{\prime }}$ $-R_{s}\Delta_{f}^{2}\delta_{k-m+n-k^{\prime }+m^{\prime }-n^{\prime }}$
	6	$|E\left\{a_{x}^{3}\right\}{|}^{2}+{\left|E\left\{a_{x}a_{y}^{2}\right\}\right|}^{2}$	$|E\left\{a_{x}^{2}a_{y}\right\}{|}^{2}$	$R_{s}^{2}\Delta_{f}\delta_{k+n+m^{\prime }}\delta_{m+k^{\prime }+n^{\prime }}$ $-R_{s}\Delta_{f}^{2}\delta_{k-m+n-k^{\prime }+m^{\prime }-n^{\prime }}$
	7	$|E\{a_{x}|a_{x}{|}^{2}{\}|}^{2}+E\{|a_{x}{|}^{2}a_{y}\}E\{a_{y}^{*}|a_{y}{|}^{2}\}$	$E\{a_{x}|a_{x}{|}^{2}\}E\{a_{x}^{*}|a_{y}{|}^{2}\}$	$R_{s}^{2}\Delta_{f}\delta_{k+n-n^{\prime }}\delta_{m+k^{\prime }-m^{\prime }}$ $-R_{s}\Delta_{f}^{2}\delta_{k-m+n-k^{\prime }+m^{\prime }-n^{\prime }}$
	8	$|E\{a_{x}|a_{x}{|}^{2}{\}|}^{2}+E\{|a_{x}{|}^{2}a_{y}^{*}\}E\{a_{y}|a_{y}{|}^{2}\}$	$E\{a_{x}|a_{y}{|}^{2}\}E\{a_{x}^{*}|a_{x}{|}^{2}\}$	$R_{s}^{2}\Delta_{f}\delta_{k-k^{\prime }+m^{\prime }}\delta_{m-n+n^{\prime }}$ $-R_{s}\Delta_{f}^{2}\delta_{k-m+n-k^{\prime }+m^{\prime }-n^{\prime }}$
	9	$|E\{a_{x}|a_{x}{|}^{2}{\}|}^{2}+|E\{a_{x}|a_{y}{|}^{2}{\}|}^{2}$	$\left|E\{\right|a_{x}{|}^{2}a_{y}{\}|}^{2}$	$R_{s}^{2}\Delta_{f}\delta_{k-k^{\prime }-n^{\prime }}\delta_{m-n-m^{\prime }}$ $-R_{s}\Delta_{f}^{2}\delta_{k-m+n-k^{\prime }+m^{\prime }-n^{\prime }}$
	10	$|E\{a_{x}|a_{x}{|}^{2}{\}|}^{2}+{\left|E\left\{a_{x}^{*}a_{y}^{2}\right\}\right|}^{2}$	$\left|E\{\right|a_{x}{|}^{2}a_{y}{\}|}^{2}$	$R_{s}^{2}\Delta_{f}\delta_{k+m^{\prime }-n^{\prime }}\delta_{m-n+k^{\prime }}$ $-R_{s}\Delta_{f}^{2}\delta_{k-m+n-k^{\prime }+m^{\prime }-n^{\prime }}$
3	1	$E\{|a_{x}{|}^{4}\}E\{|a_{x}{|}^{2}\}+E\{|a_{x}{|}^{2}|a_{y}{|}^{2}\}E\{|a_{y}{|}^{2}\}$	$E\{|a_{x}{|}^{2}\}E\{|a_{x}{|}^{2}|a_{y}{|}^{2}\}$	$-R_{s}\Delta_{f}^{2}\delta_{k-m+n-k^{\prime }+m^{\prime }-n^{\prime }}$
	2	$E^{*}\{a_{x}^{2}|a_{x}{|}^{2}\}E\left\{a_{x}^{2}\right\}+E\left\{a_{x}a_{y}\right\}E^{*}\{a_{x}a_{y}|a_{y}{|}^{2}\}$	$E\left\{a_{x}^{2}\right\}E^{*}\{a_{x}^{2}|a_{y}{|}^{2}\}$	$R_{s}^{2}\Delta_{f}\delta_{k+n}\delta_{m+k^{\prime }-m^{\prime }+n^{\prime }}$ $-R_{s}\Delta_{f}^{2}\delta_{k-m+n-k^{\prime }+m^{\prime }-n^{\prime }}$
	3	$E\{|a_{x}{|}^{4}\}E\{|a_{x}{|}^{2}\}+E\{|a_{x}{|}^{2}|a_{y}{|}^{2}\}E\{|a_{y}{|}^{2}\}$	$E\left\{a_{x}a_{y}^{*}\right\}E\{a_{x}^{*}a_{y}|a_{x}{|}^{2}\}$	$R_{s}^{2}\Delta_{f}\delta_{k-k^{\prime }}\delta_{m-n-m^{\prime }+n^{\prime }}$ $-R_{s}\Delta_{f}^{2}\delta_{k-m+n-k^{\prime }+m^{\prime }-n^{\prime }}$
	4	$E^{*}\{a_{x}^{2}|a_{x}{|}^{2}\}E\left\{a_{x}^{2}\right\}+E^{*}\left\{\right|a_{x}{|}^{2}a_{y}^{2}\}E\left\{a_{y}^{2}\right\}$	$E\left\{a_{x}a_{y}\right\}E^{*}\{a_{x}a_{y}|a_{x}{|}^{2}\}$	$R_{s}^{2}\Delta_{f}\delta_{k+m^{\prime }}\delta_{m-n+k^{\prime }+n^{\prime }}$ $-R_{s}\Delta_{f}^{2}\delta_{k-m+n-k^{\prime }+m^{\prime }-n^{\prime }}$
	5	$E\{|a_{x}{|}^{4}\}E\{|a_{x}{|}^{2}\}+E\left\{a_{x}^{*}a_{y}\right\}E\{a_{x}a_{y}^{*}|a_{y}{|}^{2}\}$	$E\{|a_{x}{|}^{2}\}E\{|a_{x}{|}^{2}|a_{y}{|}^{2}\}$	$R_{s}^{2}\Delta_{f}\delta_{k-n^{\prime }}\delta_{m-n+k^{\prime }-m^{\prime }}$ $-R_{s}\Delta_{f}^{2}\delta_{k-m+n-k^{\prime }+m^{\prime }-n^{\prime }}$
	6	$E\{|a_{x}{|}^{4}\}E\{|a_{x}{|}^{2}\}+E\left\{a_{x}a_{y}^{*}\right\}E\{a_{x}^{*}a_{y}|a_{y}{|}^{2}\}$	$E\{|a_{x}{|}^{2}\}E\{|a_{x}{|}^{2}|a_{y}{|}^{2}\}$	$-R_{s}\Delta_{f}^{2}\delta_{k-m+n-k^{\prime }+m^{\prime }-n^{\prime }}$
	7	$E\{a_{x}^{2}|a_{x}{|}^{2}\}E^{*}\left\{a_{x}^{2}\right\}+E\{|a_{x}{|}^{2}a_{y}^{2}\}E^{*}\left\{a_{y}^{2}\right\}$	$E^{*}\left\{a_{x}a_{y}\right\}E\{a_{x}a_{y}|a_{x}{|}^{2}\}$	$R_{s}^{2}\Delta_{f}\delta_{m+k^{\prime }}\delta_{k-n+m^{\prime }-n^{\prime }}$ $-R_{s}\Delta_{f}^{2}\delta_{k-m+n-k^{\prime }+m^{\prime }-n^{\prime }}$
	8	$E\{|a_{x}{|}^{4}\}E\{|a_{x}{|}^{2}\}+E\{|a_{x}{|}^{2}|a_{y}{|}^{2}\}E\{|a_{y}{|}^{2}\}$	$E\left\{a_{x}^{*}a_{y}\right\}E\{a_{x}a_{y}^{*}|a_{x}{|}^{2}\}$	$R_{s}^{2}\Delta_{f}\delta_{m-m^{\prime }}\delta_{k+n-k^{\prime }-n^{\prime }}$ $-R_{s}\Delta_{f}^{2}\delta_{k-m+n-k^{\prime }+m^{\prime }-n^{\prime }}$
	9	$E\{a_{x}^{2}|a_{x}{|}^{2}\}E^{*}\left\{a_{x}^{2}\right\}+E^{*}\left\{a_{x}a_{y}\right\}E\{a_{x}a_{y}|a_{y}{|}^{2}\}$	$E^{*}\left\{a_{x}^{2}\right\}E\{a_{x}^{2}|a_{y}{|}^{2}\}$	$R_{s}^{2}\Delta_{f}\delta_{m+n^{\prime }}\delta_{k+n-k^{\prime }+m^{\prime }}$ $-R_{s}\Delta_{f}^{2}\delta_{k-m+n-k^{\prime }+m^{\prime }-n^{\prime }}$
	10	$E\{|a_{x}{|}^{4}\}E\{|a_{x}{|}^{2}\}+E\left\{a_{x}a_{y}^{*}\right\}E\{a_{x}^{*}a_{y}|a_{y}{|}^{2}\}$	$E\left\{a_{x}a_{y}^{*}\right\}E\{a_{x}^{*}a_{y}|a_{x}{|}^{2}\}$	$R_{s}^{2}\Delta_{f}\delta_{n-k^{\prime }}\delta_{k-m+m^{\prime }-n^{\prime }}$ $-R_{s}\Delta_{f}^{2}\delta_{k-m+n-k^{\prime }+m^{\prime }-n^{\prime }}$
	11	$E^{*}\{a_{x}^{2}|a_{x}{|}^{2}\}E\left\{a_{x}^{2}\right\}+E\left\{a_{x}a_{y}\right\}E^{*}\{a_{x}a_{y}|a_{y}{|}^{2}\}$	$E\left\{a_{x}a_{y}\right\}E^{*}\{a_{x}a_{y}|a_{x}{|}^{2}\}$	$R_{s}^{2}\Delta_{f}\delta_{n+m^{\prime }}\delta_{k-m-k^{\prime }-n^{\prime }}$ $-R_{s}\Delta_{f}^{2}\delta_{k-m+n-k^{\prime }+m^{\prime }-n^{\prime }}$
	12	$E\{|a_{x}{|}^{4}\}E\{|a_{x}{|}^{2}\}+E\{|a_{x}{|}^{2}\}E\{|a_{y}{|}^{4}\}$	$E\{|a_{x}{|}^{2}\}E\{|a_{x}{|}^{2}|a_{y}{|}^{2}\}$	$R_{s}^{2}\Delta_{f}\delta_{n-n^{\prime }}\delta_{k-m-k^{\prime }+m^{\prime }}$ $-R_{s}\Delta_{f}^{2}\delta_{k-m+n-k^{\prime }+m^{\prime }-n^{\prime }}$
	13	$E\{|a_{x}{|}^{4}\}E\{|a_{x}{|}^{2}\}+E\{|a_{x}{|}^{2}|a_{y}{|}^{2}\}E\{|a_{y}{|}^{2}\}$	$E\{|a_{x}{|}^{4}\}E\{|a_{y}{|}^{2}\}$	$-R_{s}\Delta_{f}^{2}\delta_{k-m+n-k^{\prime }+m^{\prime }-n^{\prime }}$
	14	$E\{a_{x}^{2}|a_{x}{|}^{2}\}E^{*}\left\{a_{x}^{2}\right\}+E^{*}\left\{a_{x}a_{y}\right\}E\{a_{x}a_{y}|a_{y}{|}^{2}\}$	$E^{*}\left\{a_{x}a_{y}\right\}E\{a_{x}a_{y}|a_{x}{|}^{2}\}$	$R_{s}^{2}\Delta_{f}\delta_{k^{\prime }+n^{\prime }}\delta_{k-m+n+m^{\prime }}$ $-R_{s}\Delta_{f}^{2}\delta_{k-m+n-k^{\prime }+m^{\prime }-n^{\prime }}$
	15	$E\{|a_{x}{|}^{4}\}E\{|a_{x}{|}^{2}\}+E\left\{a_{x}^{*}a_{y}\right\}E\{a_{x}a_{y}^{*}|a_{y}{|}^{2}\}$	$E\left\{a_{x}^{*}a_{y}\right\}E\{a_{x}a_{y}^{*}{\left|a_{x}\right|}^{2}$	$-R_{s}\Delta_{f}^{2}\delta_{k-m+n-k^{\prime }+m^{\prime }-n^{\prime }}$
4	1	$E\{|a_{x}{|}^{6}\}+E\{|a_{x}{|}^{2}|a_{y}{|}^{4}\}$	$E\{|a_{x}{|}^{4}|a_{y}{|}^{2}\}$	$R_{s}\Delta_{f}^{2}\delta_{k-m+n-k^{\prime }+m^{\prime }-n^{\prime }}$

## Appendix E. Proof of Proposition 2

Applying the variable transformation $\tilde{k}=n$, $\tilde{m}=m$, $\tilde{n}=k$, ${\tilde{k}}^{\prime }=n^{\prime }$, ${\tilde{m}}^{\prime }=m^{\prime }$, and ${\tilde{n}}^{\prime }=k^{\prime }$ to the right-hand side of (37), we have

(A15) $$\begin{array}{c} \sum_{\begin{array}{c} \left(k,m,n\right)\in S_{i}\\ \left(k^{\prime },m^{\prime },n^{\prime }\right)\in S_{i} \end{array}}P_{k,m,n,k^{\prime },m^{\prime },n^{\prime }}\eta_{k,m,n}\eta_{k^{\prime },m^{\prime },n^{\prime }}^{*}D_{2}\left(k,m,n,k^{\prime },m^{\prime },n^{\prime }\right)\\ =\sum_{\begin{array}{c} \left(\tilde{n},\tilde{m},\tilde{k}\right)\in S_{i}\\ \left({\tilde{n}}^{\prime },{\tilde{m}}^{\prime },{\tilde{k}}^{\prime }\right)\in S_{i} \end{array}}P_{\tilde{n},\tilde{m},\tilde{k},{\tilde{n}}^{\prime },{\tilde{m}}^{\prime },{\tilde{k}}^{\prime }}\eta_{\tilde{n},\tilde{m},\tilde{k}}\eta_{{\tilde{n}}^{\prime },{\tilde{m}}^{\prime },{\tilde{k}}^{\prime }}^{*}D_{2}\left(\tilde{n},\tilde{m},\tilde{k},{\tilde{n}}^{\prime },{\tilde{m}}^{\prime },{\tilde{k}}^{\prime }\right). \end{array}$$

From definitions (16) and (22), it can be easily verified that $P_{n,m,k,n^{\prime },m^{\prime },k^{\prime }}=P_{k,m,n,k^{\prime },m^{\prime },n^{\prime }}$ and $\eta_{n,m,k}=\eta_{k,m,n}$. Moreover, based on the definition of the set $S_{i}$ in (19), it can be observed that the condition $\left(k,m,n\right)\in S_{i}$ is equivalent to $\left(n,m,k\right)\in S_{i}$, i.e., generates the same set of triplets (k,m,n). We can thus write (A15) as

$$\begin{array}{cc} \; & \sum_{\begin{array}{c} \left(k,m,n\right)\in S_{i}\\ \left(k^{\prime },m^{\prime },n^{\prime }\right)\in S_{i} \end{array}}P_{k,m,n,k^{\prime },m^{\prime },n^{\prime }}\eta_{k,m,n}\eta_{k^{\prime },m^{\prime },n^{\prime }}^{*}D_{2}\left(k,m,n,k^{\prime },m^{\prime },n^{\prime }\right)\\ \; & =\sum_{\begin{array}{c} \left(\tilde{k},\tilde{m},\tilde{n}\right)\in S_{i}\\ \left({\tilde{k}}^{\prime },{\tilde{m}}^{\prime },{\tilde{n}}^{\prime }\right)\in S_{i} \end{array}}P_{\tilde{k},\tilde{m},\tilde{n},{\tilde{k}}^{\prime },{\tilde{m}}^{\prime },{\tilde{n}}^{\prime }}\eta_{\tilde{k},\tilde{m},\tilde{n}}\eta_{{\tilde{k}}^{\prime },{\tilde{m}}^{\prime },{\tilde{n}}^{\prime }}^{*}D_{2}\left(\tilde{n},\tilde{m},\tilde{k},{\tilde{n}}^{\prime },{\tilde{m}}^{\prime },{\tilde{k}}^{\prime }\right)\\ \; & =\sum_{\begin{array}{c} \left(\tilde{k},\tilde{m},\tilde{n}\right)\in S_{i}\\ \left({\tilde{k}}^{\prime },{\tilde{m}}^{\prime },{\tilde{n}}^{\prime }\right)\in S_{i} \end{array}}P_{\tilde{k},\tilde{m},\tilde{n},{\tilde{k}}^{\prime },{\tilde{m}}^{\prime },{\tilde{n}}^{\prime }}\eta_{\tilde{k},\tilde{m},\tilde{n}}\eta_{{\tilde{k}}^{\prime },{\tilde{m}}^{\prime },{\tilde{n}}^{\prime }}^{*}D_{1}\left(\tilde{k},\tilde{m},\tilde{n},{\tilde{k}}^{\prime },{\tilde{m}}^{\prime },{\tilde{n}}^{\prime }\right), \end{array}$$

which proves the proposition.

## Appendix F. Coefficients in (38)

**Table A5:** Expressions for the coefficients in (38).

Name	Value	Name	Value
$a_{1}$	$2E^{3}\left\{\right|a_{x}{|}^{2}\}+E\{|a_{x}{|}^{2}\}E^{2}\left\{\right|a_{y}{|}^{2}\}+|E\left\{a_{x}a_{y}^{*}\right\}{|}^{2}E\{|a_{y}{|}^{2}\}$	$a_{1}^{\prime }$	$2E\{|a_{x}{|}^{2}\}|E\left\{a_{x}a_{y}^{*}\right\}{|}^{2}$
$a_{2}$	$4E\{|a_{x}{|}^{2}\}|E\left\{a_{x}^{2}\right\}{|}^{2}+E\{|a_{x}{|}^{2}\}|E\left\{a_{y}^{2}\right\}{|}^{2}$ +|E{axay}|2E{|ay|2}+2Re{E{axay}E{ax∗ay}E∗{ay2}}	$a_{2}^{\prime }$	$2E\{|a_{x}{|}^{2}\}|E\left\{a_{x}a_{y}\right\}{|}^{2}$ $+2E^{*}\left\{a_{x}^{2}\right\}E\left\{a_{x}a_{y}\right\}E\left\{a_{x}a_{y}^{*}\right\}$
$a_{3}$	$E\{|a_{x}{|}^{2}\}|E\left\{a_{x}^{2}\right\}{|}^{2}+|E\left\{a_{x}a_{y}\right\}{|}^{2}E\{|a_{y}{|}^{2}\}$	$a_{3}^{\prime }$	$E\left\{a_{x}^{2}\right\}E^{*}\left\{a_{x}a_{y}\right\}E\left\{a_{x}^{*}a_{y}\right\}$
$b_{1}$	$4|E\{a_{x}|a_{x}{|}^{2}{\}|}^{2}+E\{|a_{x}{|}^{2}a_{y}\}E\{a_{y}^{*}|a_{y}{|}^{2}\}$ $+E\{|a_{x}{|}^{2}a_{y}^{*}\}E\{a_{y}|a_{y}{|}^{2}\}+|E\{a_{x}|a_{y}{|}^{2}{\}|}^{2}+{\left|E\left\{a_{x}^{*}a_{y}^{2}\right\}\right|}^{2}$	$b_{1}^{\prime }$	$E\{a_{x}^{*}|a_{x}{|}^{2}\}E\{a_{x}|a_{y}{|}^{2}\}$ $+2|E\{|a_{x}{|}^{2}a_{y}{\}|}^{2}$
$b_{2}$	$2|E\{a_{x}|a_{x}{|}^{2}{\}|}^{2}+E\{|a_{x}{|}^{2}a_{y}^{*}\}E\{a_{y}|a_{y}{|}^{2}\}+|E\{a_{x}|a_{y}{|}^{2}{\}|}^{2}$	$b_{2}^{\prime }$	$2|E\{|a_{x}{|}^{2}a_{y}{\}|}^{2}$
		$b_{3}^{\prime }$	$E\{a_{x}^{*}|a_{x}{|}^{2}\}E\{a_{x}|a_{y}{|}^{2}\}+|E\left\{a_{x}^{2}a_{y}^{*}\right\}{|}^{2}$
$b_{4}$	$|E\left\{a_{x}^{3}\right\}{|}^{2}+{\left|E\left\{a_{x}a_{y}^{2}\right\}\right|}^{2}$	$b_{4}^{\prime }$	$|E\left\{a_{x}^{2}a_{y}\right\}{|}^{2}$
$c_{1}$	$-3E\{|a_{x}{|}^{2}\}|E\left\{a_{x}^{2}\right\}{|}^{2}+E^{*}\{a_{x}^{2}|a_{x}{|}^{2}\}E\left\{a_{x}^{2}\right\}$ $-E\left\{a_{x}a_{y}\right\}E\left\{a_{x}^{*}a_{y}\right\}E^{*}\left\{a_{y}^{2}\right\}-2|E\left\{a_{x}a_{y}\right\}{|}^{2}E\{|a_{y}{|}^{2}\}$ $+E\left\{a_{x}a_{y}\right\}E^{*}\{a_{x}a_{y}|a_{y}{|}^{2}\}$	$c_{1}^{\prime }$	$-2E\left\{a_{x}^{2}\right\}E^{*}\left\{a_{x}a_{y}\right\}E\left\{a_{x}^{*}a_{y}\right\}$ $-|E\left\{a_{x}^{2}\right\}{|}^{2}E\{|a_{y}{|}^{2}\}$ $+E\left\{a_{x}^{2}\right\}E^{*}\{a_{x}^{2}|a_{y}{|}^{2}\}$
$c_{2}$	$-8E^{3}\left\{\right|a_{x}{|}^{2}\}-4E\{|a_{x}{|}^{2}\}|E\left\{a_{x}^{2}\right\}{|}^{2}+4E\{|a_{x}{|}^{4}\}E\{|a_{x}{|}^{2}\}$ $-3E\{|a_{x}{|}^{2}\}E^{2}\left\{\right|a_{y}{|}^{2}\}-E\{|a_{x}{|}^{2}\}|E\left\{a_{y}^{2}\right\}{|}^{2}$ $+E\{|a_{x}{|}^{2}\}E\{|a_{y}{|}^{4}\}+E\{|a_{x}{|}^{2}|a_{y}{|}^{2}\}E\{|a_{y}{|}^{2}\}$ $-5|E\left\{a_{x}a_{y}^{*}\right\}{|}^{2}E\{|a_{y}{|}^{2}\}-|E\left\{a_{x}a_{y}\right\}{|}^{2}E\{|a_{y}{|}^{2}\}$ +2Re{E{ax∗ay}E{axay∗|ay|2}−E{axay}E{ax∗ay}E∗{ay2}}	$c_{2}^{\prime }$	$-6E\{|a_{x}{|}^{2}\}|E\left\{a_{x}a_{y}^{*}\right\}{|}^{2}$ $-2E\{|a_{x}{|}^{2}\}|E\left\{a_{x}a_{y}\right\}{|}^{2}$ $-2E^{2}\left\{\right|a_{x}{|}^{2}\}E\{|a_{y}{|}^{2}\}$ $+2E\{|a_{x}{|}^{2}\}E\{|a_{x}{|}^{2}|a_{y}{|}^{2}\}$ $+2E\left\{a_{x}a_{y}^{*}\right\}E\{a_{x}^{*}a_{y}|a_{x}{|}^{2}\}$ $-2E^{*}\left\{a_{x}^{2}\right\}E\left\{a_{x}a_{y}\right\}E\left\{a_{x}a_{y}^{*}\right\}$
$c_{3}$	$-6E\{|a_{x}{|}^{2}\}|E\left\{a_{x}^{2}\right\}{|}^{2}+2E^{*}\{a_{x}^{2}|a_{x}{|}^{2}\}E\left\{a_{x}^{2}\right\}$ $-E\{|a_{x}{|}^{2}\}|E\left\{a_{y}^{2}\right\}{|}^{2}+E^{*}\left\{\right|a_{x}{|}^{2}a_{y}^{2}\}E\left\{a_{y}^{2}\right\}$ $-E^{*}\left\{a_{x}a_{y}\right\}E\left\{a_{x}a_{y}^{*}\right\}E\left\{a_{y}^{2}\right\}-2|E\left\{a_{x}a_{y}\right\}{|}^{2}E\{|a_{y}{|}^{2}\}$ +E{axay}E∗{axay|ay|2}−2Re{E∗{axay}E{axay∗}E{ay2}}	$c_{3}^{\prime }$	$4E\{|a_{x}{|}^{2}\}|E\left\{a_{x}a_{y}\right\}{|}^{2}$ $+2E\left\{a_{x}a_{y}\right\}E^{*}\{a_{x}|a_{x}{|}^{2}a_{y}\}$ $-2E^{*}\left\{a_{x}^{2}\right\}E\left\{a_{x}a_{y}\right\}E\left\{a_{x}a_{y}^{*}\right\}$
$c_{4}$	$-2E^{3}\left\{\right|a_{x}{|}^{2}\}-E\{|a_{x}{|}^{2}\}|E\left\{a_{x}^{2}\right\}{|}^{2}-E\{|a_{x}{|}^{2}\}E^{2}\left\{\right|a_{y}{|}^{2}\}$ $+E\{|a_{x}{|}^{2}|a_{y}{|}^{2}\}E\{|a_{y}{|}^{2}\}-|E\left\{a_{x}a_{y}^{*}\right\}{|}^{2}E\{|a_{y}{|}^{2}\}$ $-|E\left\{a_{x}a_{y}\right\}{|}^{2}E\{|a_{y}{|}^{2}\}+E\{|a_{x}{|}^{4}\}E\{|a_{x}{|}^{2}\}$	$c_{4}^{\prime }$	$-2E\{|a_{x}{|}^{2}\}|E\left\{a_{x}a_{y}^{*}\right\}{|}^{2}$ $+E\left\{a_{x}a_{y}^{*}\right\}E\{a_{x}^{*}a_{y}|a_{x}{|}^{2}\}$ $-E\left\{a_{x}^{2}\right\}E^{*}\left\{a_{x}a_{y}\right\}E\left\{a_{x}^{*}a_{y}\right\}$
		$c_{5}^{\prime }$	$-2E\{|a_{x}{|}^{2}\}|E\left\{a_{x}a_{y}\right\}{|}^{2}$ $+E\left\{a_{x}a_{y}\right\}E^{*}\{a_{x}a_{y}|a_{x}{|}^{2}\}$ $-E^{*}\left\{a_{x}^{2}\right\}E\left\{a_{x}a_{y}\right\}E\left\{a_{x}a_{y}^{*}\right\}$ $-|E\left\{a_{x}^{2}\right\}{|}^{2}E\{|a_{y}{|}^{2}\}$ −2Re{E{ax2}E∗{axay}E{ax∗ay}}
		$c_{6}^{\prime }$	$-2E\{|a_{x}{|}^{2}\}|E\left\{a_{x}a_{y}\right\}{|}^{2}$ $+E\left\{a_{x}a_{y}\right\}E^{*}\{a_{x}a_{y}|a_{x}{|}^{2}\}$ $-E\left\{a_{x}^{2}\right\}E^{*}\left\{a_{x}a_{y}\right\}E\left\{a_{x}^{*}a_{y}\right\}$
$d_{1}$	$12E^{3}\left\{\right|a_{x}{|}^{2}\}+18E\{|a_{x}{|}^{2}\}|E\left\{a_{x}^{2}\right\}{|}^{2}-{\left|E\left\{a_{x}^{3}\right\}\right|}^{2}$ $-9|E\{a_{x}|a_{x}{|}^{2}{\}|}^{2}-9E\{|a_{x}{|}^{4}\}E\{|a_{x}{|}^{2}$} $+E\{|a_{x}{|}^{6}\}+4E\{|a_{x}{|}^{2}\}E^{2}\left\{\right|a_{y}{|}^{2}\}+2E\{|a_{x}{|}^{2}\}|E\left\{a_{y}^{2}\right\}{|}^{2}$ $-E\{|a_{x}{|}^{2}\}E\{|a_{y}{|}^{4}\}-4E\{|a_{x}{|}^{2}|a_{y}{|}^{2}\}E\{|a_{y}{|}^{2}\}$ $-4|E\{a_{x}|a_{y}{|}^{2}{\}|}^{2}+8|E\left\{a_{x}a_{y}^{*}\right\}{|}^{2}E\{|a_{y}{|}^{2}\}$ $+8|E\left\{a_{x}a_{y}\right\}{|}^{2}E\{|a_{y}{|}^{2}\}-|E\left\{a_{x}a_{y}^{2}\right\}{|}^{2}-{\left|E\left\{a_{x}^{*}a_{y}^{2}\right\}\right|}^{2}$ +E{|ax|2|ay|4}+2Re{4E{axay}E{ax∗ay}E∗{ay2} $-E\left\{a_{x}a_{y}^{*}\right\}E\{a_{x}^{*}a_{y}|a_{y}{|}^{2}\}-3E\{a_{x}^{2}|a_{x}{|}^{2}\}E^{*}\left\{a_{x}^{2}\right\}$ $-2E\left\{a_{x}a_{y}\right\}E^{*}\{a_{x}a_{y}|a_{y}{|}^{2}\}-2E\{|a_{x}{|}^{2}a_{y}\}E\{a_{y}^{*}|a_{y}{|}^{2}\}$ $-E\{|a_{x}{|}^{2}a_{y}^{2}\}E^{*}\left\{a_{y}^{2}\right\}\}$	$d_{1}^{\prime }$	$8E\{|a_{x}{|}^{2}\}|E\left\{a_{x}a_{y}^{*}\right\}{|}^{2}-{\left|E\left\{a_{x}^{2}a_{y}^{*}\right\}\right|}^{2}$ $+8E\{|a_{x}{|}^{2}\}|E\left\{a_{x}a_{y}\right\}{|}^{2}+4E^{2}\left\{\right|a_{x}{|}^{2}\}E\{|a_{y}{|}^{2}\}$ $-4E\{|a_{x}{|}^{2}\}E\{|a_{x}{|}^{2}|a_{y}{|}^{2}\}-E\{|a_{x}{|}^{4}\}E\{|a_{y}{|}^{2}\}$ $-2E\{a_{x}^{*}|a_{x}{|}^{2}\}E\{a_{x}|a_{y}{|}^{2}\}$ $-2E\left\{a_{x}^{2}\right\}E^{*}\{a_{x}^{2}|a_{y}{|}^{2}\}-E\{a_{x}|a_{x}{|}^{2}\}E\{a_{x}|a_{y}{|}^{2}\}$ $-4|E\{|a_{x}{|}^{2}a_{y}{\}|}^{2}+E\{|a_{x}{|}^{4}|a_{y}{|}^{2}\}$ $-4E\left\{a_{x}a_{y}^{*}\right\}E\{a_{x}^{*}a_{y}|a_{x}{|}^{2}\}$ $-4E\left\{a_{x}a_{y}\right\}E^{*}\{a_{x}a_{y}|a_{x}{|}^{2}\}$ $+2|E\left\{a_{x}^{2}\right\}{|}^{2}E\{|a_{y}{|}^{2}\}-|E\left\{a_{x}^{2}a_{y}\right\}{|}^{2}$ +8Re{E{ax2}E∗{axay}E{ax∗ay}}

## Appendix G. Proof of Proposition 3

Since $D_{1}\left(k,m,n,k^{\prime },m^{\prime },n^{\prime }\right)=D_{2}\left(k^{\prime },m^{\prime },n^{\prime },k,m,n\right)$, the left-hand side of (39) can be written as

(A16) $$\begin{array}{c} \begin{array}{cc} \; & \sum_{\begin{array}{c} \left(k,m,n\right)\in S_{i}\\ \left(k^{\prime },m^{\prime },n^{\prime }\right)\in S_{i} \end{array}}P_{k,m,n,k^{\prime },m^{\prime },n^{\prime }}\eta_{k,m,n}\eta_{k^{\prime },m^{\prime },n^{\prime }}^{*}D_{1}\left(k,m,n,k^{\prime },m^{\prime },n^{\prime }\right)\\ \; & =\sum_{\begin{array}{c} \left(k,m,n\right)\in S_{i}\\ \left(k^{\prime },m^{\prime },n^{\prime }\right)\in S_{i} \end{array}}P_{k,m,n,k^{\prime },m^{\prime },n^{\prime }}\eta_{k,m,n}\eta_{k^{\prime },m^{\prime },n^{\prime }}^{*}D_{2}\left(k^{\prime },m^{\prime },n^{\prime },k,m,n\right). \end{array} \end{array}$$

Using the change of variables $\tilde{k}=k^{\prime }$, $\tilde{m}=m^{\prime }$, $\tilde{n}=n^{\prime }$, ${\tilde{k}}^{\prime }=k$, ${\tilde{m}}^{\prime }=m$, and ${\tilde{n}}^{\prime }=n$, the right-hand side of (A16) can be equivalently expressed as

(A17a) $$\begin{array}{cc} \; & \sum_{\begin{array}{c} \left(k,m,n\right)\in S_{i}\\ \left(k^{\prime },m^{\prime },n^{\prime }\right)\in S_{i} \end{array}}P_{k,m,n,k^{\prime },m^{\prime },n^{\prime }}\eta_{k,m,n}\eta_{k^{\prime },m^{\prime },n^{\prime }}^{*}D_{2}\left(k^{\prime },m^{\prime },n^{\prime },k,m,n\right) \end{array}$$

(A17b) $$\begin{array}{cc} \; & =\sum_{\begin{array}{c} \left({\tilde{k}}^{\prime },{\tilde{m}}^{\prime },{\tilde{n}}^{\prime }\right)\in S_{i}\\ \left(\tilde{k},\tilde{m},\tilde{n}\right)\in S_{i} \end{array}}P_{{\tilde{k}}^{\prime },{\tilde{m}}^{\prime },{\tilde{n}}^{\prime },\tilde{k},\tilde{m},\tilde{n}}\eta_{\tilde{k},\tilde{m},\tilde{n}}^{*}\eta_{{\tilde{k}}^{\prime },{\tilde{m}}^{\prime },{\tilde{n}}^{\prime }}D_{2}\left(\tilde{k},\tilde{m},\tilde{n},{\tilde{k}}^{\prime },{\tilde{m}}^{\prime },{\tilde{n}}^{\prime }\right) \end{array}$$

(A17c) $$\begin{array}{cc} \; & =\sum_{\begin{array}{c} \left(\tilde{k},\tilde{m},\tilde{n}\right)\in S_{i}\\ \left({\tilde{k}}^{\prime },{\tilde{m}}^{\prime },{\tilde{n}}^{\prime }\right)\in S_{i} \end{array}}P_{\tilde{k},\tilde{m},\tilde{n},{\tilde{k}}^{\prime },{\tilde{m}}^{\prime },{\tilde{n}}^{\prime }}^{*}\eta_{\tilde{k},\tilde{m},\tilde{n}}^{*}\eta_{{\tilde{k}}^{\prime },{\tilde{m}}^{\prime },{\tilde{n}}^{\prime }}D_{2}\left(\tilde{k},\tilde{m},\tilde{n},{\tilde{k}}^{\prime },{\tilde{m}}^{\prime },{\tilde{n}}^{\prime }\right) \end{array}$$

(A17d) $$\begin{array}{cc} \; & =(\sum_{\begin{array}{c} \left(\tilde{k},\tilde{m},\tilde{n}\right)\in S_{i}\\ \left({\tilde{k}}^{\prime },{\tilde{m}}^{\prime },{\tilde{n}}^{\prime }\right)\in S_{i} \end{array}}P_{\tilde{k},\tilde{m},\tilde{n},{\tilde{k}}^{\prime },{\tilde{m}}^{\prime },{\tilde{n}}^{\prime }}\eta_{\tilde{k},\tilde{m},\tilde{n}}\eta_{{\tilde{k}}^{\prime },{\tilde{m}}^{\prime },{\tilde{n}}^{\prime }}^{*}D_{2}\left(\tilde{k},\tilde{m},\tilde{n},{\tilde{k}}^{\prime },{\tilde{m}}^{\prime },{\tilde{n}}^{\prime }\right){)}^{*}, \end{array}$$

where in the step from (A17b) to (A17c), we have used (A14). Equation (A17d) is identical to the right-hand side of (39) up to the variable relabelling $\tilde{k}\to k,\tilde{m}\to m,\tilde{n}\to n,{\tilde{k}}^{\prime }\to k^{\prime },{\tilde{m}}^{\prime }\to m^{\prime },{\tilde{n}}^{\prime }\to n^{\prime }$, which proves the proposition.

## Appendix H. Proof of Lemma 1

To prove the statement about dimensionality of the sets $T_{l,i}$, we take as an example the cases for l=1,2,3. In these instances, the sets $T_{l,i}$∀i∈Z are identified by 5 linear constraints on the set of variables $\left(k,m,n,k^{\prime },m^{\prime },n^{\prime }\right)\in {\left\{0,1,\ldots ,W-1\right\}}^{6}$ given by (i) the 2 linearly independent constraints, $\left(k,m,n\right)\in S_{i}$ and $\left(k^{\prime },m^{\prime },n^{\prime }\right)\in S_{i}$ and (ii) the 3 linearly independent constraints induced by the condition $D^{\left(l\right)}=1$ for l=1,2,3 (see Table 7). Then, let $A_{l}≜\left[a_{1}^{T};a_{2}^{T};\ldots ;a_{5}^{T}\right]$ be a 5×6 matrix in which the rows $a_{k}$, k=1,2,…,5, describe each of these 5 linear combinations, $x≜[k,m,n,k^{\prime },m^{\prime },n^{\prime }]$, and $y_{i}=\left[i,i,0,0,0\right]$. Thus, the set $T_{l,i}$ can be equivalently defined as

(A18) $$T_{l,i}=\left\{x\in {\left\{0,1,\ldots ,W-1\right\}}^{6}:A_{l}x=y_{i}\right\}.$$

From (A18), it can be seen that $T_{l,i}$ is a vector space for which the number of dimensions is given by

(A19) $$\dim\left\{T_{l,i}\right\}=6-\mathrm{rank}\left(A_{l}\right).$$

Due to the construction of the delta products $D^{\left(l\right)},\;l\in \left\{1,2,3\right\}$, it can be shown that the rows of $A_{l}$ are linearly dependent under the relationship $a_{1}-a_{2}=\pm a_{3}\pm a_{4}\pm a_{5}$. Hence, ∀l∈{1,2,3} and i∈Z, we have rank$(A_{l})=4$. As a result, from (A19), $\dim\left\{T_{l,i}\right\}=2,\;\forall \;l\in \left\{1,2,3\right\}$, and i∈Z.

For l∈{4,5,…,10}, we have that $T_{l,i}$ is identified by 4 linear constraints, 2 of them related to the $S_{i}$ set and 2 related to the condition $D^{\left(l\right)}=1$. Furthermore, it can be seen that $a_{1}-a_{2}=\pm a_{3}\pm a_{4}$, hence leading to rank $\left(A_{l}\right)=3,\;\forall \;l\in \left\{4,5,\ldots ,10\right\}$ and $\dim\{T_{l,i}\}=3$. Finally, based on similar arguments, one can show that rank $(A_{l})=2$ for l=11 and dim$\{T_{l,i}\}=4$, which proves the lemma.

## Appendix I. Proof of Theorem 2

The limit of a sequence of distributions $f(\Delta_{f})$ is defined as the distribution $\tilde{f}$ such that [18] (Section 2.2)

(A20) $$\langle \tilde{f},\psi \rangle =\lim_{\Delta_{f}\to 0}\langle f\left(\Delta_{f}\right),\psi \rangle ,\;\forall \;\psi ,$$

where

(A21) $$\langle f,\psi \rangle ≜\int_{-\infty }^{\infty }f\left(f\right)\psi \left(f\right)\;df$$

denotes the functional corresponding to the distribution f applied to a generic test function ψ [18] (Section 1.1). In particular, the delta distribution centered in $f_{0}$ is defined as

(A22) $$\langle \delta_{f_{0}},\psi \rangle ≜\int_{-\infty }^{\infty }\delta \left(f-f_{0}\right)\psi \left(f\right)df=\psi \left(f_{0}\right).$$

Based on (A21), we have for the distribution $S_{x}\left(f,N_{s},L_{s}\right)$ in (40),

(A23a) $$\begin{array}{cc} \langle S_{x}\left(f,N_{s},L_{s}\right),\psi \rangle & ={\left(\frac{8}{9}\right)}^{2}\gamma^{2}\Delta_{f}[R_{s}^{3}\Delta_{f}^{2}(\Phi_{1}\int_{-\infty }^{\infty }\sum_{i=-\infty }^{\infty }\sum_{T_{1,i}}P\delta \left(f-i\Delta_{f}\right)\psi \left(f\right)df+\ldots \\ +\Phi_{3}\int_{-\infty }^{\infty }\sum_{i=-\infty }^{\infty }\sum_{T_{3,i}} & P\delta \left(f-i\Delta_{f}\right)\psi \left(f\right)df)+R_{s}^{2}\Delta_{f}^{3}(\Psi_{1}\int_{-\infty }^{\infty }\sum_{i=-\infty }^{\infty }\sum_{T_{4,i}}P\delta \left(f-i\Delta_{f}\right)\psi \left(f\right)df+\ldots \\ +\Lambda_{6}\int_{-\infty }^{\infty }\sum_{i=-\infty }^{\infty }\sum_{T_{10,i}} & P\delta \left(f-i\Delta_{f}\right)\psi \left(f\right)df)+R_{s}\Delta_{f}^{4}\Xi_{1}\int_{-\infty }^{\infty }\sum_{i=-\infty }^{\infty }\sum_{T_{11,i}}P\delta \left(f-i\Delta_{f}\right)\psi \left(f\right)df] \end{array}$$

(A23b) $$\begin{array}{c} \begin{array}{cc} \; & ={\left(\frac{8}{9}\right)}^{2}\gamma^{2}[R_{s}^{3}\Delta_{f}^{3}(\Phi_{1}\sum_{i=-\infty }^{\infty }\sum_{T_{1,i}}P\psi \left(i\Delta_{f}\right)+\ldots +\Phi_{3}\sum_{i=-\infty }^{\infty }\sum_{T_{3,i}}P\psi \left(i\Delta_{f}\right))\\ +R_{s}^{2}\Delta_{f}^{4}(\Psi_{1}\sum_{i=-\infty }^{\infty }\sum_{T_{4,i}} & P\psi \left(i\Delta_{f}\right)+\ldots +\Lambda_{6}\sum_{i=-\infty }^{\infty }\sum_{T_{10,i}}P\psi \left(i\Delta_{f}\right))+R_{s}\Delta_{f}^{5}\Xi_{1}\sum_{i=-\infty }^{\infty }\sum_{T_{11,i}}P\psi \left(i\Delta_{f}\right)], \end{array} \end{array}$$

where we have used (A22) in the step between (A23a) and (A23b).

Now, we want to show that all the terms in (A23b) are multidimensional Riemann sums, which then will converge to multidimensional integrals in the limit for $\Delta_{f}\to 0$. From (16), (22), and (35), it can be seen that the terms $P\psi (i\Delta_{f})$ are samples on a multidimensional grid of step $\Delta_{f}$ of the multivariate function

(A24) $$\begin{array}{c} \begin{array}{cc} \tilde{P}\left(f_{1},f_{2},f_{3},f_{1}^{\prime },f_{2}^{\prime },f_{3}^{\prime }\right)≜P\left(f_{1}\right)P^{*}\left(f_{2}\right)P\left(f_{3}\right)P^{*}\left(f_{1}^{\prime }\right)P\left(f_{2}^{\prime }\right)P^{*}\left(f_{3}^{\prime }\right)\eta (f_{1},f_{2},f_{3}, & N_{s},L_{s})\eta^{*}\left(f_{1}^{\prime },f_{2}^{\prime },f_{3}^{\prime },N_{s},L_{s}\right),\\ \; & f_{1},f_{2},f_{3},f_{1}^{\prime },f_{2}^{\prime },f_{3}^{\prime }\in R. \end{array} \end{array}$$

Moreover, $\Delta_{f}^{t(l)}$, which represents the power of $\Delta_{f}$ multiplying the *l*th element in (A23b), where

(A25) $$t\left(l\right)≜\left\{\begin{array}{cc} 3 & \mathrm{for}\;\;l=1,2,3;\\ 4 & \mathrm{for}\;\;l=4,\ldots ,10;\\ 5 & \mathrm{for}\;\;l=11, \end{array}\right.$$

is a measure of the t(l)th dimensional hypercube in $R^{t(l)}$ for which the side measures $\Delta_{f}$. Hence, to prove that each term in (A23b) converges to a sum of multiple integrals of the multivariate functions $\tilde{P_{l}}\psi \left(f\right)$, we simply need to show that the dimensionality of the summation sets, i.e., $Z\times T_{l,i}$, is equal to t(l), i.e., $\dim\left\{T_{l,i}\right\}=t\left(l\right)-1$, for l=1,…,11, and ∀i∈Z. This can be easily verified comparing Lemma 1 to (A25). Defining the subspaces of $R^{6}$

(A26) $$Q_{l}\left(f\right)≜\left\{\left(f_{1},f_{2},f_{3},f_{1}^{\prime },f_{2}^{\prime },f_{3}^{\prime }\right)\in R^{6}\cap G_{l}:f_{1}-f_{2}+f_{3}=f,\;f_{1}^{\prime }-f_{2}^{\prime }+f_{3}^{\prime }=f\right\},$$

where $G_{l}$ is the set defined by the condition $D^{\left(l\right)}=1$, and by replacing the variables $\left(k,m,n,k^{\prime },m^{\prime },n^{\prime }\right)\to \left(f_{1},f_{2},f_{3},f_{1}^{\prime },f_{2}^{\prime },f_{3}^{\prime }\right),$ we have

(A27a) $$\begin{array}{cc} \lim_{\Delta_{f}\to 0}\langle S_{x}\left(f,N_{s},L_{s}\right),\psi \rangle ={\left(\frac{8}{9}\right)}^{2}\gamma^{2} & [R_{s}^{3}(\Phi_{1}\int_{-\infty }^{\infty }\underset{Q_{1}\left(f\right)}{\int ...\int }\tilde{P}\left(f_{1},\ldots ,f_{3}^{\prime }\right)\psi \left(f\right)df_{1}\;\ldots \;df_{3}^{\prime }\;df+\ldots \\ +\Phi_{3}\int_{-\infty }^{\infty }\underset{Q_{3}\left(f\right)}{\int ...\int }\tilde{P}\left(f_{1},\ldots ,f_{3}^{\prime }\right)\psi \left(f\right) & df_{1}\;\ldots df_{3}^{\prime }\;df)+R_{s}^{2}(\Psi_{1}\int_{-\infty }^{\infty }\underset{Q_{4}\left(f\right)}{\int ...\int }\tilde{P}\left(f_{1},\ldots ,f_{3}^{\prime }\right)\psi \left(f\right)df_{1}\;\ldots df_{3}^{\prime }\;df+\ldots \\ +\Lambda_{6}\int_{-\infty }^{\infty }\underset{Q_{10}\left(f\right)}{\int ...\int }\tilde{P}\left(f_{1},\ldots ,f_{3}^{\prime }\right)\psi \left(f\right) & df_{1}\;\ldots df_{3}^{\prime }\;df)+R_{s}\Xi_{1}\int_{-\infty }^{\infty }\underset{Q_{11}\left(f\right)}{\int ...\int }\tilde{P}\left(f_{1},\ldots ,f_{3}^{\prime }\right)\psi \left(f\right)df_{1}\;\ldots df_{3}^{\prime }\;df] \end{array}$$

(A27b) $$\begin{array}{cc} ={\left(\frac{8}{9}\right)}^{2}\gamma^{2}[R_{s}^{3} & (\Phi_{1}\int_{-\infty }^{\infty }\int_{-\infty }^{\infty }\int_{-\infty }^{\infty }{\tilde{P}}_{1}\left(f_{1},f_{2},f\right)\psi \left(f\right)df_{1}\;df_{2}\;df+\ldots \\ +\Phi_{3}\int_{-\infty }^{\infty }\int_{-\infty }^{\infty }\int_{-\infty }^{\infty }{\tilde{P}}_{3}\left(f_{1},f_{2},f\right)\psi \left(f\right) & df_{1}\;df_{2}\;df)+R_{s}^{2}(\Psi_{1}\underset{R^{4}}{\int ...\int }{\tilde{P}}_{4}\left(f_{1},\ldots ,f_{3},f\right)\psi \left(f\right)df_{1}\;\ldots \;df_{3}\;df+\ldots \\ +\Lambda_{6}\underset{R^{4}}{\int ...\int }{\tilde{P}}_{10}\left(f_{1},\ldots ,f_{3},f\right)\psi \left(f\right) & df_{1}\;\ldots \;df_{3}\;df)+R_{s}\Xi_{1}\underset{R^{5}}{\int ...\int }{\tilde{P}}_{11}\left(f_{1},\ldots ,f_{4},f\right)\psi \left(f\right)df_{1}\;\ldots \;df_{4}\;df]. \end{array}$$

In the steps from (A27a) to (A27b), we have replaced in each integral the function $\tilde{P}$ with its constrained instance over $Q_{l}\left(f\right)$

$${\tilde{P}}_{l}≜\tilde{P}\left(f_{1},f_{2},f_{3},f_{1}^{\prime },f_{2}^{\prime },f_{3}^{\prime }\right){|}_{\left(f_{1},f_{2},f_{3},f_{1}^{\prime },f_{2}^{\prime },f_{3}^{\prime }\right)\in Q_{l}\left(f\right)},$$

and explicitly expressed the dimensionality of the integrals based on the dimension of their corresponding integration domains $Q_{l}\left(f\right)$. By construction (see (A26)), $\dim\{Q_{l}\left(f\right)\}=t\left(l\right)-1$, for l=1,…,11.

Finally, using (A20) and comparing definition (A21) with (A27b), we obtain

$$\begin{array}{cc} {\bar{S}}_{x}\left(f,N_{s},L_{s}\right) & =\lim_{\Delta_{f}\to 0}S_{x}\left(f,N_{s},L_{s}\right)={\left(\frac{8}{9}\right)}^{2}\gamma^{2}[R_{s}^{3}(\Phi_{1}\int_{-\infty }^{\infty }\int_{-\infty }^{\infty }{\tilde{P}}_{1}\left(f_{1},f_{2},f\right)df_{1}\;df_{2}+\ldots \\ +\Phi_{3} & \int_{-\infty }^{\infty }\int_{-\infty }^{\infty }{\tilde{P}}_{3}\left(f_{1},f_{2},f\right)df_{1}\;df_{2})+R_{s}^{2}(\Psi_{1}\int_{-\infty }^{\infty }\int_{-\infty }^{\infty }\int_{-\infty }^{\infty }{\tilde{P}}_{4}\left(f_{1},f_{2},f_{3},f\right)df_{1}\;df_{2}\;df_{3}\;+\ldots \\ +\Lambda_{6} & \int_{-\infty }^{\infty }\int_{-\infty }^{\infty }\int_{-\infty }^{\infty }{\tilde{P}}_{10}\left(f_{1},f_{2},f_{3},f\right)df_{1}\;df_{2}\;df_{3})+R_{s}\Xi_{1}\underset{R^{4}}{\int ...\int }{\tilde{P}}_{11}\left(f_{1},\ldots ,f_{4},f\right)df_{1}\ldots \;df_{4}], \end{array}$$

which, defining

(A28) $$\chi_{l}\left(f\right)≜\left\{\;\begin{array}{c} \int_{R_{2}}{\tilde{P}}_{l}\left(f_{1},f_{2},f\right)df_{1}\;df_{2}=\int_{-\frac{R_{s}}{2}}^{\frac{R_{s}}{2}}\int_{-\frac{R_{s}}{2}}^{\frac{R_{s}}{2}}{\tilde{P}}_{l}\left(f_{1},f_{2},f\right)df_{1}\;df_{2},\;\;\;\;\;\;\;\;\;l=1,2,3;\\ \int_{R_{3}}{\tilde{P}}_{l}\left(f_{1},f_{2},f_{3},f\right)df_{1}\;df_{2}\;df_{3}=\int_{-\frac{R_{s}}{2}}^{\frac{R_{s}}{2}}\int_{-\frac{R_{s}}{2}}^{\frac{R_{s}}{2}}\int_{-\frac{R_{s}}{2}}^{\frac{R_{s}}{2}}{\tilde{P}}_{l}\left(f_{1},f_{2},f_{3},f\right)df_{1}\;df_{2}\;df_{3},\;\;\;\;\;l=4,\ldots ,10;\\ \int_{R_{4}}{\tilde{P}}_{11}\left(f_{1},\ldots ,f_{4},f\right)df_{1}\ldots df_{4}=\underset{R_{4}}{\int ...\int }{\tilde{P}}_{11}\left(f_{1},\ldots ,f_{4},f\right)df_{1}\ldots df_{4},\;\;\;\;\;\;\;\;l=11, \end{array}\right.$$

with $R_{4}≜{\left[-R_{s}/2,R_{s}/2\right]}^{4}$, proves the theorem. The second equalities in (A28) are justified by the form of the functions ${\tilde{P}}_{l}$ (see Table 8) which, due to the assumption of strictly bandlimited pulses, have limited support within the hybercube ${\left[-R_{s}/2,R_{s}/2\right]}^{t(l)-1}$. To derive the explicit expressions for $\chi_{l}\left(f\right)$ in Table 8, we used (A28) and the property $P\left(-f\right)=P^{*}\left(f\right)$, which stems from the fact that p(t) is assumed to be a real-valued function (see Section 3.1).
